# Endothelial KSR2 regulated by genetic variation protects against atherosclerosis through AMPKα1 stabilization

**DOI:** 10.7150/thno.122864

**Published:** 2026-01-01

**Authors:** Ming Liu, Xiangrui Fu, Hui Zhang, Jinyu pan, Qiufeng Jia, Chengrui Zhang, Fengshuang An

**Affiliations:** 1 National Key Laboratory for Innovation and Transformation of Luobing Theory; The Key Laboratory of Cardiovascular Remodeling and Function Research, Chinese Ministry of Education, Chinese National Health Commission and Chinese Academy of Medical Sciences; Department of Cardiology, Qilu Hospital of Shandong University, Jinan, China.; 2 Department of Cardiology, The First Affiliated Hospital of Shandong First Medical University, Jinan, China.; 3 Shandong University of Traditional Chinese Medicine, Jinan, China.

**Keywords:** single nucleotide polymorphism, coronary artery disease, endothelial dysfunction, glycolysis, ubiquitin-proteasome system

## Abstract

**Rationale:** The single nucleotide polymorphism (SNP) rs11830157 within the scaffold protein kinase suppressor of Ras 2 (*KSR2*) locus is strongly associated with the incidence of coronary artery disease (CAD), yet its functional role remains undefined. This study aimed to investigate the potential impact of rs11830157 polymorphism on atherosclerosis and to elucidate the underlying molecular mechanisms.

Methods**:** Dual-luciferase reporter assays, chromatin immunoprecipitation (ChIP), electrophoretic mobility shift assays (EMSA), and CRISPR/Cas9 gene-editing techniques were used to investigate the regulatory role of the SNP rs11830157. To assess the role of *KSR2* in atherosclerosis, we utilized global *KSR2* knockout mice fed a high-fat diet ad libitum, pair-fed global *KSR2* and *Apoe* (Apolipoprotein E) double knockout mice, and mice with endothelial-specific *KSR2* overexpression mediated by AAV9-*ICAM2*.

Results**:** Genetic analyses identified SNP rs12822146, in linkage disequilibrium with rs11830157 and located within an endothelial enhancer, as a regulator of *KSR2* expression via differential binding of the transcriptional repressor *XBP1s*. *KSR2* expression was significantly reduced in endothelial cells within atherosclerotic plaques in both humans and mice. Using multiple *KSR2* gene-edited mouse models, we demonstrated that endothelial *KSR2* protects against atherosclerosis by suppressing inflammation and apoptosis. Mechanistic studies revealed that *KSR2* competes with *CRBN* for binding to the K52 site of *AMPKα1*, inhibiting *CRL4A^CRBN^* E3 ubiquitin ligase complex-mediated K48-linked polyubiquitination and proteasomal degradation of *AMPKα1*. The subsequently activated *AMPK* signaling pathway maintains glycolytic balance in endothelial cells, ultimately exerting anti-inflammatory and anti-apoptotic effects.

Conclusions**:** Our findings provide the first comprehensive molecular explanation of the rs12822146-*KSR2*-atherosclerosis axis, with important implications for both primary prevention and secondary treatment of CAD.

## Introduction

Atherosclerotic cardiovascular disease (ASCVD), a pathological condition manifesting as cardiovascular and cerebrovascular complications including myocardial infarction and ischemic stroke, remains the leading cause of worldwide morbidity and mortality 1. The vascular endothelium, a continuous cellular layer lining the cardiovascular system, serves as a dynamic interface​ and plays a critical role as a central hub for numerous regulatory processes within this homeostatic network 2. Under physiological conditions, this extensive organ maintains systemic tissue and organ homeostasis; yet under pathological conditions, endothelial dysfunction drives both local and systemic features of ASCVD 3. Despite substantial progress in the study of endothelial dysfunction over the past few decades, our understanding of endothelial dysfunction remains incomplete.

Genetic factors substantially influence susceptibility to coronary artery disease (CAD), and over the past decade, more than 400 independent loci have been identified by genome-wide association studies (GWAS) as contributing to CAD and related clinical outcomes 4-8. However, linking these GWAS variants to the underlying mechanisms of disease remains a significant challenge. The high-frequency genetic variant rs11830157 (T > G, with G as the risk allele), characterized by a minor allele frequency (MAF) of 29% in general populations, has been consistently identified in multiple Mendelian randomization studies across diverse ethnic cohorts as a risk locus for CAD, with carriers of the risk allele G demonstrating a significantly elevated disease incidence 6, 9. Single nucleotide polymorphism (SNP) rs11830157 is situated within the second intron of *KSR2* (kinase suppressor of Ras 2), a gene mapped to the human chromosomal locus 12q24. This genomic region has been genetically associated with metabolic disturbances including dyslipidemia and dysglycemia, as well as cardiovascular pathologies encompassing hypertension, coronary artery disease, and myocardial infarction 10-14. Similar to Kinase Suppressor of Ras 1 (*KSR1*) 15-17, *KSR2* acts as a key scaffold in the Raf/MEK/ERK signaling cascade, controlling both the magnitude and persistence of ERK signaling. The molecular divergence between *KSR2* and its paralog *KSR1* is functionally substantiated by the identification of a unique interdomain sequence spanning CA2-CA3 motifs in *KSR2*. This evolutionarily conserved region has been mechanistically demonstrated to engage in direct physical interaction with AMP-activated protein kinase (*AMPK*) - the principal regulator of cellular energy balance - thereby potentiating *AMPK* signaling activation 18, 19. *KSR2*^-/-^ mice exhibit decreased *AMPK* signaling, resulting in defective fatty acid oxidation and triglyceride accumulation, thereby promoting obesity and insulin resistance. Similarly, specific *KSR2* mutations in humans with early-onset obesity disrupt ERK pathway activation or hinder *AMPK* binding 13. These findings establish *KSR2* as a central regulator of systemic metabolism in both mice and humans. Atherosclerosis, the pathological foundation of coronary artery disease, is closely linked to metabolic dysfunction, yet the functional role of *KSR2* in this disease process remains unknown.

In this study, we identified that it is not the SNP rs11830157, but rather the allele polymorphism of rs12822146, which is in linkage disequilibrium with rs11830157, that modulates *KSR2* expression in endothelial cells via distinct interactions with the transcriptional repressor *XBP1s*. Using *KSR2*-modified mice subjected to both ad libitum and pair-feeding regimens, along with adeno-associated virus-mediated endothelial-specific *KSR2* overexpression, we demonstrate that endothelial *KSR2* confers cell-autonomous atheroprotection. *In vitro*, we found that *KSR2* activates the *AMPK* signaling pathway through a non-canonical mechanism, maintaining glycolytic balance in endothelial cells and thereby mitigating endothelial inflammation and apoptosis. Mechanistically, *KSR2* competitively binds to the K52 site of *AMPKα1* with *CRBN*, inhibiting the *CRL4A^CRBN^* E3 ubiquitin ligase complex-mediated K48-linked polyubiquitination and proteasomal degradation of *AMPKα1*. Furthermore, endothelial-specific *CRBN* overexpression or selective activation of endothelial *AMPK* signalling *in vivo* established that *KSR2* slows plaque progression through a *CRBN*-*AMPK*-dependent axis. In addition, endothelial-specific *CRBN* knockdown similarly reduced inflammation and apoptosis, delaying plaque development in *Apoe*^-/-^ mice. In summary, we identified a regulatory role of the CAD-associated SNP rs12822146 in controlling endothelial *KSR2* expression and uncovered a novel function of the endothelial *KSR2*-*CRL4A^CRBN^*-*AMPK* axis in vascular inflammation, apoptosis, and atherosclerosis.

## Methods

### Human Samples

Human coronary artery specimens were obtained from patients undergoing heart transplantation at The First Affiliated Hospital of Shandong First Medical University. The study protocol received approval from the institutional ethics committee (Approval No. S047) and complied with the principles of the Declaration of Helsinki. Written informed consent was provided by all participants or their legally authorized representatives. All specimens were obtained voluntarily, without coercion, and no organs or tissues were procured from executed prisoners or individuals detained for their political or religious beliefs. Detailed clinical information is provided in Table S1.

### Animal Studies

All animal experiments were approved by the Institutional Animal Care and Use Committee of Shandong University (Approval No. QLYY-2024-266) and conducted in accordance with the NIH Guide for the Care and Use of Laboratory Animals (NIH publication No. 86-23, revised 1985). Male mice were maintained in a specific pathogen-free (SPF) environment with controlled conditions, including a temperature of 23°C, 60% relative humidity, and a 12-hour light/12-hour dark cycle.

*KSR2* knockout (*KSR2*^-/-^) mice were generated by CRISPR/Cas9-mediated deletion of exon 3 of the *Ksr2* gene (Shanghai Model Organisms Center, Shanghai, China), and bred from *Ksr2*⁺/⁻ heterozygotes. *Apoe*^-/-^ mice on a C57BL/6J background were obtained from GemPharmatech (Nanjing, China). Double knockout *Apoe*^-/-^*Ksr2*^-/-^ mice were generated by crossing *Apoe*^-/-^*Ksr2*^+/-^ mice. Mouse genotyping was carried out by PCR using tail-derived genomic DNA (see Table S2 for primers). Throughout the 12-week study, all mice received a high-fat diet (40% fat, 1.25% cholesterol; TP28521, Trophic Animal Feed High-tech Co., Ltd., Nantong, China). Pair-feeding was conducted as previously described 20.

For endothelial-specific *KSR2* overexpression, an AAV9 vector carrying the *Ksr2* coding sequence under the *ICAM2* promoter (BioSune Biotechnology, Shanghai, China) was injected via tail vein into 8-week-old *Apoe*^-/-^ mice (5 × 10^11^ vg/mouse). Control mice received AAV9-*ICAM2*-mock. To activate endothelial *AMPK* signaling, AAV9-*ICAM2*-constitutively active *AMPKα1* (AAV9-*AMPKα1*) or control AAV9-mock was injected into *Apoe*^-/-^ or *Apoe*^-/-^*Ksr2*^-/-^ mice under the same dosing regimen. Endothelial-specific *CRBN* knockdown was achieved by tail vein injection of AAV9-*ICAM2*-sh*CRBN* or control AAV9-*ICAM2*-shNC (BioSune Biotechnology; 5 × 10^11^ vg/mouse) into 8-week-old *Apoe*^-/-^ mice. To simultaneously overexpress *KSR2* and *CRBN* in the endothelium, a mixture of AAV9-*ICAM2*-*KSR2* and AAV9-*ICAM2*-*CRBN* (BioSune Biotechnology; 5 × 10¹¹ vg of each virus per mouse) was delivered via tail vein injection to 8-week-old *Apoe*^-/-^ mice. Following viral administration, all treated mice were subsequently fed a high-fat diet for 8 weeks.

Mice were anesthetized with pentobarbital sodium (50 mg/kg; intraperitoneal injection; once prior to procedures; Sigma-Aldrich, St. Louis, MO, USA). Mice were euthanized with an overdose of pentobarbital sodium (150 mg/kg, i.p.) and subsequent cervical dislocation, following institutional and NIH animal care standards.

### Cell Culture

HUVECs (CRL-1370) and HEK293T cells (CRL-11268) were obtained from ATCC (Rockville, USA). Primary VSMCs, MAECs, and peritoneal macrophages were isolated from mice as described 21, 22. Cells were cultured under the following conditions: HUVECs and MAECs in ECM (Sciencell) with 15% FBS, 1% P/S, and 1% growth supplement; HEK293T and macrophages in DMEM (Gibco; 5.5 mM glucose) with 10% FBS and 1% P/S; VSMCs in SMCM (Sciencell; 5.5 mM glucose) with 2% FBS, 1% P/S, and 1% growth supplement. All cells were kept at 37°C with 5% CO₂.

### Luciferase Assay

Genomic fragments flanking rs11830157 or rs12822146 (both alleles) were synthesized (Integrated DNA Technologies) and cloned into the pGL4.26 luciferase reporter vector (BioSune Biotechnology, Shanghai, China), upstream of a minimal promoter. Constructs were co-transfected with PRL-TK Renilla luciferase control vector (BioSune) into MAECs, VSMCs, or peritoneal macrophages using Lipofectamine 2000 (Thermo Fisher Scientific, Cat. No. 11668019) at ~80% confluency. After 48 h, luciferase activity was measured using the Dual-Luciferase Reporter Assay Kit (MeilunBio, Cat. No. MA0518).

### Chromatin Immunoprecipitation (ChIP) Assay

ChIP was carried out in HUVECs, VSMCs and macrophages employing the SimpleChIP® Enzymatic Chromatin IP Kit (#9005, Cell Signaling Technology) in strict accordance with the manufacturer's instructions. Crosslinked chromatin was sheared and immunoprecipitated with anti- *H3K27ac* (Abcam, ab4729), anti-*H3K4me1* (Abcam, ab176877), anti-*XBP1s* (Proteintech, 24168-1-AP), anti-*MEIS1* (Cell Signaling Technology, #67260), anti-*GATA2* (Proteintech, 11103-1-AP) or control rabbit *IgG*. Immunoprecipitated DNA was purified and analyzed by qPCR. Primer sequences are listed in Table S3.

### Electrophoretic Mobility Shift Assay (EMSA)

Biotin-labeled DNA probes were prepared using the Biotin-Labeling Kit (GS008, Beyotime), EMSA was conducted using the Chemiluminescent EMSA Kit (GS009, Beyotime). Nuclear extracts from HUVECs (5 μg) were incubated with 0.2 μM biotin-labeled probe for 20 minutes at room temperature. For competition assays, a 50-fold molar excess of unlabeled probe was included. For supershift assays, nuclear extracts were pre-incubated with 1 μg anti-*XBP1s* antibody on ice for 30 min. To assess allele-specific binding at rs12822146, increasing concentrations (10× to 100×) of unlabeled alt probe or 100× non-specific competitor was added with biotin-labeled ref probe. Complexes were resolved on 6% native polyacrylamide gels (0.5× TBE, 100 V, 90 min), blotted onto nylon membranes, and visualized by chemiluminescence. All steps were carried out on ice unless otherwise specified.

### CRISPR-Cas9 Genome Editing

To generate Δrs12822146 HUVECs, two sgRNAs upstream (sgRNA1: TGGCCCCTAGTGAACGGCAG; sgRNA2: TCTTGGACACCATACGACCA) and two downstream (sgRNA3: CTGGTATCCGGTTACAAAGC; sgRNA4: TCTGAAGGTCACTCCTATAT) of the rs12822146 locus were designed using an online tool. Plasmids encoding each sgRNA were transfected into HEK293T cells, followed by puromycin selection 48 h post-transfection. Genomic DNA sequencing confirmed editing activity by the presence of double peaks at all sgRNA target sites.

sgRNA1 and sgRNA3 were selected for lentiviral packaging. Lentivirus was produced and transduced into HUVECs using Lipofectamine 2000 (Thermo Fisher Scientific). After puromycin selection, single-cell clones were expanded, and genomic DNA sequencing confirmed deletion at the rs12822146 locus.

To introduce the homozygous rs12822146 T allele in HUVECs, CRISPR/Cas9-mediated base editing was performed. A sgRNA (sgRNA-A1: GGTGGAGGAACTGAGCGTCAGGG) was designed using CRISPOR and complexed with Cas9 protein to form a ribonucleoprotein (RNP) complex. A single-stranded DNA donor template (sequence: AAGGCATCCCAGTGTCTACGGCCAAGCAGCTACGTGCCCCTAACTTTTTCAAGCAAGGAGCCTAGGATTGAAAGCAACAATGCAGCCCTGATGCTCAGTTCCTCCACCCTACGTGATCATGATGGCA) containing the C-to-T substitution was co-electroporated with the RNP into cells using the Neon™ Transfection System. After transfection, single-cell clones were isolated and expanded. Genomic DNA was extracted from candidate clones, and the target region was amplified by PCR with primers (F: CGAGGTTCCTGCCAGTTTCT; R: GAGTTCTTTGGGGAGTGGGG). Successful introduction of the homozygous mutation was confirmed by Sanger sequencing and alignment with the wild-type sequence using SnapGene software.

### Atherosclerotic Plaque Analysis

After euthanizing the mice with pentobarbital sodium, perfusion was carried out using PBS, followed by tissue fixation with 4% paraformaldehyde. Aortas were dissected, adventitia removed, and stained *en face* with Oil Red O (O1516, Sigma-Aldrich). Lesion area was quantified as percentage of total aortic surface using ImageJ (NIH, USA).

Aortic root analysis was performed using hearts cryoprotected in 30% sucrose, embedded in OCT (Sakura Finetek), and sectioned into 6-7 µm slices. Staining was carried out with Oil Red O, H&E (G1120, Solarbio), or Masson's trichrome (G1340, Solarbio).

Immunofluorescence staining was performed on aortic root sections using primary antibodies: anti-*CD31* (Proteintech, Rosemont, IL, USA; 1:5000), anti-*α-SMA* (Abcam, Cambridge, UK; 1:300), anti-*CD68* (Abclonal, Wuhan, China; 1:100), anti-*KSR2* (Santa Cruz Biotechnology, Dallas, TX, USA; 1:100) followed by fluorophore-conjugated secondary antibodies and DAPI counterstaining.

Immunohistochemistry involved endogenous peroxidase blocking and incubation with primary antibodies against *MOMA-2* (Abcam; 1:200), *α-SMA* (Abcam; 1:200), *VCAM1* (Abcam; 1:50), *ICAM1* (Abcam; 1:100), *IL-1β* (Proteintech; 1:200), *TNF-α* (Proteintech; 1:400), and *KSR2* (Santa Cruz; 1:100), followed by HRP-conjugated secondary antibodies and DAB chromogenic detection (ZSGB-Bio, Beijing, China).

All histologically stained sections were imaged utilizing a Pannoramic digital slide scanner (3D HISTECH, Budapest, Hungary). Subsequent quantitative and morphological analyses were performed with Pannoramic Viewer software (3D HISTECH) and ImageJ (NIH, USA).

### *En Face* Aorta Staining

*En face* immunostaining of mouse aortas was performed as previously described 14. Briefly, isolated aortas were fixed with 4% paraformaldehyde (Sigma-Aldrich, St. Louis, MO, USA) and blocked with 5% bovine serum albumin (BSA; Sigma-Aldrich). Samples were incubated overnight at 4 °C with primary antibodies: anti-*CD31* (Proteintech, Rosemont, IL, USA; 1:5000), anti-*KSR2* (Santa Cruz Biotechnology, Dallas, TX, USA; 1:100), anti-*PFKFB3* (Proteintech; 1:200), and anti-*HK2* (Abcam, Cambridge, UK; 1:100). After PBS washes, Alexa Fluor-conjugated secondary antibodies (Thermo Fisher Scientific, Waltham, MA, USA) were applied for 1 h at RT, followed by DAPI nuclear staining (Abcam).

We assessed apoptosis with TMR Red TUNEL staining (Roche, IN) following kit instructions. After 1-h incubation at 37°C in darkness, aortas were DAPI-counterstained and mounted for imaging. Fluorescence imaging employed a Zeiss LSM 710 confocal microscope. The ratio of TUNEL-positive to *CD31*-positive cells was calculated using ImageJ (NIH) to assess endothelial apoptosis.

### Transfection and Infection

Full-length or 373T mutant cDNAs of human *KSR2* and *CRBN* were amplified by PCR and subcloned into pcDNA3.1-Myc vectors. Full-length, truncated, and site-directed mutant constructs of human *AMPKα1* and *CUL4A* were cloned into pcDNA3.1-Flag, and wild-type, K48, or K63 mutant ubiquitin constructs were cloned into pcDNA3.1-HA. Empty pcDNA3.1-Myc, -Flag, or -HA vectors served as controls. Transient transfection of all plasmids was performed using Lipofectamine 2000 (Invitrogen, Carlsbad, CA) following the manufacturer's recommended protocols.

Small interfering RNAs (siRNAs) targeting *XBP1s*, *KSR2*, *CUL4A*, *CRBN*, *MKRN1*, *RMND5A*, *TRIM28*, *PEDF*, *FBXo48*, *RNF44*, and *AMPKα1* were synthesized by BioSune Biotechnology (Shanghai, China). siRNA sequences are provided in Table S3. Transfections were performed using Lipofectamine RNAiMAX (Invitrogen, USA) following the manufacturer's protocol.

### THP-1 Adhesion Assay

We labeled THP-1 monocytes with 5 μM Calcein-AM (Beyotime, C2012) and co-cultured them with HUVEC monolayers (37°C, 30 min) for adhesion quantification. After removal of non-adherent cells by PBS washing, adherent THP-1 cells were visualized using fluorescence microscopy (Calcein, green). Quantification was performed in five random fields per well using ImageJ.

### Nuclear and Cytoplasmic Protein Extraction

Nuclear and cytoplasmic compartments of HUVECs were separated with a commercial extraction kit (Beyotime P0028) following modified protocols. Extracted proteins were stored at -20 °C until analysis.

### Total Protein Extraction and Western Blotting

Protein lysates were isolated from HUVECs, human atherosclerotic plaques, and murine tissues using RIPA buffer (R0010, Solarbio, China) containing protease (CW2200, CWBIO) and phosphatase inhibitors (CW2383, CWBIO). Protein concentrations were measured via BCA assay (P0012, Beyotime, China). Equal protein aliquots (20-30 μg) underwent electrophoretic separation on 10% SDS-polyacrylamide gels and electroblotting onto PVDF membranes (Millipore). Post-blocking (5% milk), membranes were incubated with primary antibodies (4°C, overnight) and HRP-secondaries. Signals were detected by ECL (Millipore WBULS0500) on an Amersham Imager 680RGB (GE Healthcare), with band intensities quantified in ImageJ relative to housekeeping proteins.

### RNA Extraction and RT-qPCR

Total RNA was isolated from HUVECs, human atherosclerotic plaques, and murine tissues using TRIzol™ Reagent (Invitrogen, 15596018). cDNA synthesis was performed with HiScript III RT SuperMix (Vazyme, R323-01), incorporating genomic DNA removal. Quantitative PCR amplification utilized ChamQ Universal SYBR qPCR Master Mix (Vazyme, Q711) on a LightCycler® 96 System (Roche, Switzerland). Relative mRNA expression levels were calculated via the comparative ΔΔCt method normalized to endogenous controls. Primer sequences are listed in Table S2. All experiments were conducted according to the reagent manufacturer's instructions.

### Co-Immunoprecipitation (Co-IP)

Co-IP assays were conducted to investigate protein-protein interactions in HUVECs, HEK293T cells, human atherosclerotic plaques, and murine tissues. Samples were lysed in native buffer (Beyotime P0013, China) supplemented with protease inhibitors. Following centrifugation to clarify lysates, supernatants were incubated with immunoprecipitation-grade primary antibodies or species-matched *IgG* controls at 4°C for 1 h. Protein complexes were subsequently captured via overnight incubation with protein A/G magnetic beads (MedChemExpress HY-K0202, USA). Immunoprecipitates underwent stringent washing with PBST (PBS containing 0.5% Tween-20), eluted in SDS loading buffer by boiling, and subjected to immunoblot analysis.

### Metabolic Measurements

Glycolytic flux in HUVECs was quantified via extracellular acidification rate (ECAR) measurements using the Seahorse XF96 Analyzer (Agilent Technologies). Cells (1.5 × 10⁴/well) pretreated with 100 μM oxLDL for 24 h were analyzed in XF Base Medium containing 2 mM glutamine (pH 7.4). Sequential injections of glucose (10 mM), oligomycin (1 µM), and 2-deoxyglucose (2-DG, 50 mM) were applied to evaluate basal glycolysis, glycolytic reserve, and glycolytic capacity, respectively.

Lactate production was quantified as an additional indicator of glycolytic activity using the Lactate Assay Kit (BC2230, Solarbio, Beijing, China) according to the manufacturer's instructions.

The ATP/ADP ratio in cultured cells was measured with a commercial chemiluminescence assay kit (E-BC-F004, Elabscience) following the manufacturer's protocol. Cell lysates were prepared and reacted with a luciferase-based working solution. Luminescence was recorded (L1) immediately to reflect ATP content. After converting endogenous ADP to ATP in the same sample, total luminescence (L2) was measured. The ATP/ADP ratio was derived as (L2 - L1)/L1.

### Statistical Analysis

Statistical analyses were conducted using GraphPad Prism 9.5 (GraphPad Software, San Diego, CA, USA). Data represent mean ± SEM, with biological replicate numbers (N) specified in figure legends; technical replicates were neither averaged nor used as the statistical unit. Exact n values are provided in the corresponding figure legends. Normality was assessed via Shapiro-Wilk testing. Variance homogeneity was determined by F-test (two groups) or Brown-Forsythe test (≥3 groups).

- Two-group comparisons: means were compared with an unpaired, two-tailed Student's t-test when data were normally distributed and variances were equal; otherwise, the Mann-Whitney U test was applied (including all cases where n ≤ 5).
- Multi-group comparisons (> 2 groups): one-way ANOVA followed by Tukey's post-hoc test was used when normality and equal variance were satisfied; if either assumption was violated or n ≤ 5 per group, the Kruskal-Wallis test with Dunn's multiple-comparison correction was employed.
- Two-factor designs: two-way ANOVA followed by Tukey's post-hoc test.
- For the multiple t-tests (e.g., RT-qPCR results), to ensure the robustness of the analysis results, nonparametric Mann-Whitney U tests (for two groups) were employed for all between-group comparisons. The P values were adjusted using the Bonferroni method to control the family-wise error rate.

Statistical significance was defined as P < 0.05. Effect sizes for key pairwise comparisons were quantified with Cohen's d (mean difference / pooled SD) and interpreted as small (0.2), medium (0.5), or large (0.8).

## Results

### Functional SNP rs12822146 regulates endothelial *KSR2* expression

**Figure 1:**
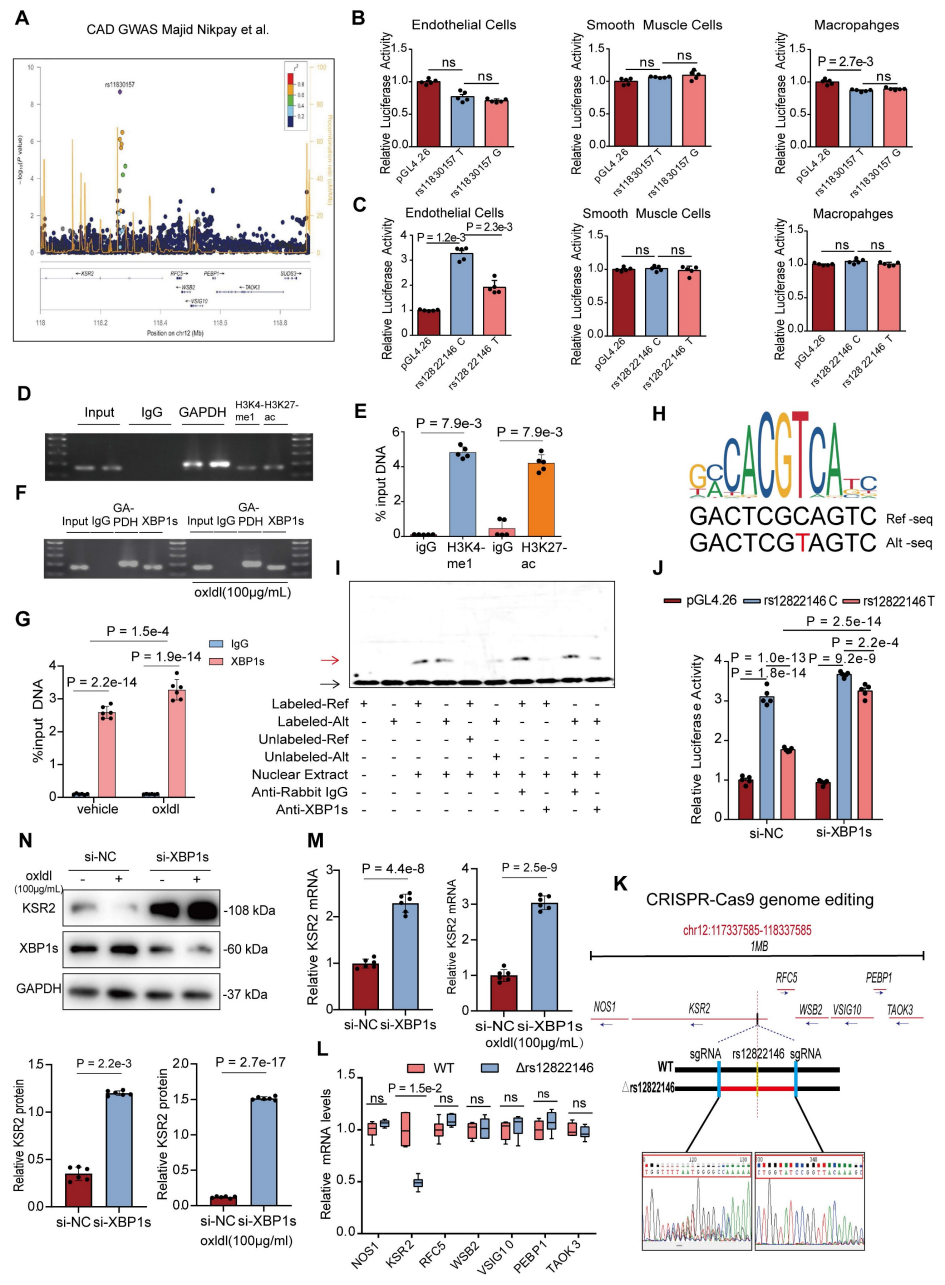
** Coronary artery disease (CAD) related single nucleotide polymorphism (SNP) rs12822146 allele polymorphism is associated with the expression of the KSR2 gene in endothelial cells. A,** Regional association plots illustrating the association between the rs11830157 locus and CAD in the GWAS meta-analysis (OR = 1.12, P = 2.12 × 10⁻⁹). **B** and** C,** Luciferase data showing allele-specific activities for rs11830157(T, reference allele; G, alternative allele) and rs12822146(C, reference allele; T, alternative allele) in Mouse Aortic Endothelial Cells (MAECs), primary vascular smooth muscle cells (VSMCs), peritoneal macrophages. n = 5, Kruskal-Wallis test with Dunn's multiple comparisons test due to small simple sizes. **D** and** E**, Chromatin immunoprecipitation (ChIP) analyses showing enhancer activity in the region surrounding rs12822146 in HUVECs. Pulldown of *H3K4me1* (mono-methyl-histone H3 lysine 4), *H3K27ac* (acetyl-histone H3 lysine 27) was performed to assess the enrichment of chromatin fragments containing rs12822146. n = 5, 2-tailed unpaired Mann-Whitney U test due to small simple sizes. **F** and** G**, ChIP analyses showing specific binding of *XBP1s* to the region surrounding rs12822146 in HUVECs, with enhanced enrichment under oxLDL stimulation. n = 6, 2-way ANOVA with Tukey post hoc test. **H,** JASPAR database predictions indicating allele-specific binding of the transcription factor *XBP1* at the rs12822146 locus. **I,** Electrophoretic mobility shift assays (EMSA) demonstrating allele-specific binding of nuclear proteins from endothelial cells to the rs12822146 C and T alleles. Competitive EMSA confirms the specificity of these interactions, while supershift EMSA with *XBP1s* antibody validates direct binding of *XBP1s* to both alleles. Black arrow indicates the free biotin-labeled probe; red arrow denotes the protein-probe complex. **J,** HUVECs were transfected with either CTR siRNA (si-NC) or *XBP1s* siRNA (si-*XBP1s*), along with pGL4.26 or luciferase constructs containing rs12822146 alleles, for 48 hours. Luciferase activity was then measured. n = 5, 2-way ANOVA with Tukey post hoc test. **K and L,** CRISPR-Cas9-mediated deletion of the genomic region surrounding rs12822146 in HUVECs reduces *KSR2* expression. **(K)** Schematic illustrating the CRISPR-Cas9 editing strategy targeting the rs12822146 locus. **(L)** Real-time quantitative PCR (RT-qPCR) analysis showing the expression of protein-coding genes within ±1 Mb of rs12822146 in edited HUVECs. n = 6 per group, genes differential expression were evaluated using a family of 2-tailed Mann-Whitney U tests (multiple t-test framework, one per gene) with Bonferroni adjustment to control the family-wise error rate. **M** and** N,** HUVECs were transfected with control siRNA (si-NC) or *XBP1s* siRNA (si-*XBP1s*) for 48 h, followed by treatment with or without oxLDL (100 μg/mL) for 24 h. **(M)** RT-qPCR and **(N)** western blot showing changes in *KSR2* expression. n = 6, except for the left panel of N, which was analyzed using a 2-tailed unpaired Mann-Whitney U test due to non-normal distribution, all other results were analyzed using a 2-tailed unpaired Student's t-test. Ref-seq, Reference Sequence; Alt-seq, alternative sequence; ns, not significant.

A GWAS meta-analysis of multi-ethnic cohorts (77% European, 13% South Asian, 6% East Asian) demonstrated a recessive-model association between rs11830157 and coronary artery disease risk (OR = 1.12, p = 2.12×10^-9^) (Figure 1A) 6. Subsequent analyses revealed no pleiotropic associations between rs11830157 and established cardiovascular risk factors 11. Corroborating this, a GWAS in type 1 diabetes populations demonstrated persistent association of rs11830157 with CAD incidence following multivariable adjustment for conventional risk factors 9. Collectively, these findings implicate rs11830157 in CAD pathogenesis through direct vascular mechanisms, independent of traditional risk pathways. While the absence of cis-eQTL associations in vascular tissues (GTEx v8) 23 suggests non-canonical regulatory mechanisms, the genomic context of rs11830157 warrants functional investigation. To determine whether the rs11830157 locus resides within functional genomic elements of vascular wall cells, we cloned 500-1000 bp regions flanking the SNP—containing either the risk or non-risk allele—upstream of a minimal promoter driving firefly luciferase, and assessed its regulatory activity in endothelial cells, smooth muscle cells, and macrophages. No allele-specific differences in enhancer activity were observed for rs11830157 (Figure 1B). Thus, it is more like a genetic marker than a functional variant. Based on the population distribution in the GWAS study 6, we screened highly linkage disequilibrium SNPs to rs11830157 in the CHB, GIH, and CEU populations on the 1000 Genomes Project website using a threshold of r^2^ ≥ 0.8 (Table S4), all eight SNPs are located within a ≈18kb region of intron 2 of *KSR2* (chromosome 12: 117827344-117845711). Analysis of publicly available ENCODE datasets on the UCSC genome browser [Expanded Encyclopaedias of DNA Elements in the Human and Mouse Genomes] reveals DnaseI, CTCF, *H3K4me1*(mono-methyl-histone H3 lysine 4), and *H3K27ac*(acetyl-histone H3 lysine 27) hypersensitivity signals near rs12822146, suggesting this SNP resides in an open chromatin region (Figure S1A). The dual-luciferase assay results indicated that in endothelial cells, rs12822146 exhibited differential activity based on genotype, with the non-risk C allele being significantly more active than the risk T allele (3.27 ± 0.26 vs 1.92 ± 0.27; Cohen's D = 5.09). In contrast, luciferase plasmids containing both allelic sites did not show transcriptional activity in macrophages or smooth muscle cells (Figure 1C). Chromatin immunoprecipitation (ChIP) analyses further revealed significant enrichment of *H3K27ac* and *H3K4me1* at DNA around rs12822146 in human endothelial cells compared to *IgG* controls (Figure 1D-E), whereas no significant enrichment was observed in macrophages or vascular smooth muscle cells (Figure S1B-C). These findings collectively indicate that rs12822146 resides within an endothelial cell-specific active enhancer region.

*In silico* screening with the PROMO and JASPAR databases predicted that *XBP1*, *MEIS1*, and *GATA2* differentially bind to the chromatin region encompassing rs12822146. Chromatin immunoprecipitation assays showed that only antibodies against *XBP1s*—the transcriptionally active spliced isoform of *XBP1*—selectively enriched rs12822146-flanking fragments over *IgG* controls in human endothelial cells; this enrichment was further amplified by oxLDL stimulation (Figure 1F-G, Figure S1D-E). No significant binding was detected in macrophages or vascular smooth-muscle cells (Figure S1F-G). *In silico* motif analysis indicated that *XBP1* preferentially recognizes the risk T allele at rs12822146 (Figure 1H). We next experimentally validated this allelic bias in *XBP1s* binding. Electrophoretic mobility shift assays (EMSA) revealed binding of nuclear proteins from endothelial cells to rs12822146 alleles C and T, with competitive EMSA confirming allele-specific displacement using excess unlabeled probes. Crucially, supershift EMSA results demonstrated a marked reduction in the DNA-protein complex band upon addition of the *XBP1s* antibody, indicating a direct interaction between nuclear *XBP1s* and the DNA probe (Figure 1I, Figure S1H). Furthermore, silencing *XBP1s* expression in HUVECs using si-*XBP1s* significantly reduced the allele-specific differences in transcriptional activity at rs12822146, as shown by dual-luciferase assays (Figure 1J). Together, these findings indicate that rs12822146 resides within an active endothelial enhancer, and its allelic variants modulate enhancer activity via differential recruitment of *XBP1s*.

To explore whether the alleles of rs12822146 regulate gene expression in endothelial cells, we used CRISPR-Cas9 to delete a 565bp region encompassing rs12822146 in HUVECs (Figure 1K). Within a 1-Mb region flanking the SNP, seven protein-coding genes were identified: *NOS1*, *KSR2*, *RFC5*, *WSB2*, *VSIG10*, *PEBP1*, and *TAOK3*. Subsequent quantitative real-time PCR (RT-qPCR) analysis revealed that deletion of the rs12822146-containing region significantly reduced *KSR2* expression in HUVECs (1 ± 0.17 vs 0.49 ± 0.06, Cohen's D = 4.00), while expression of the other six genes remained unchanged (Figure 1L). Moreover, knockdown of *XBP1s* led to a marked upregulation of *KSR2* in endothelial cells, regardless of the presence of the atherosclerosis-related stimulus oxLDL, suggesting that *XBP1s* functions as a transcriptional repressor of *KSR2* (Figure 1M-N). Since *XBP1s* is a marker of endoplasmic reticulum (ER) stress 24, we further validated this using the ER stress inhibitor TUDCA. The results, similar to those observed with si-*XBP1s*, showed that TUDCA significantly decreased *XBP1s* expression in endothelial cells and notably increased *KSR2* gene expression, both in the presence and absence of oxLDL stimulation (Figure S1I-J). Given that the pathological basis of coronary artery disease is atherosclerosis, we hypothesize that endothelial *KSR2* may contribute to the progression of atherosclerosis.

### Endothelial *KSR2* expression is selectively downregulated during atherosclerosis progression

**Figure 2:**
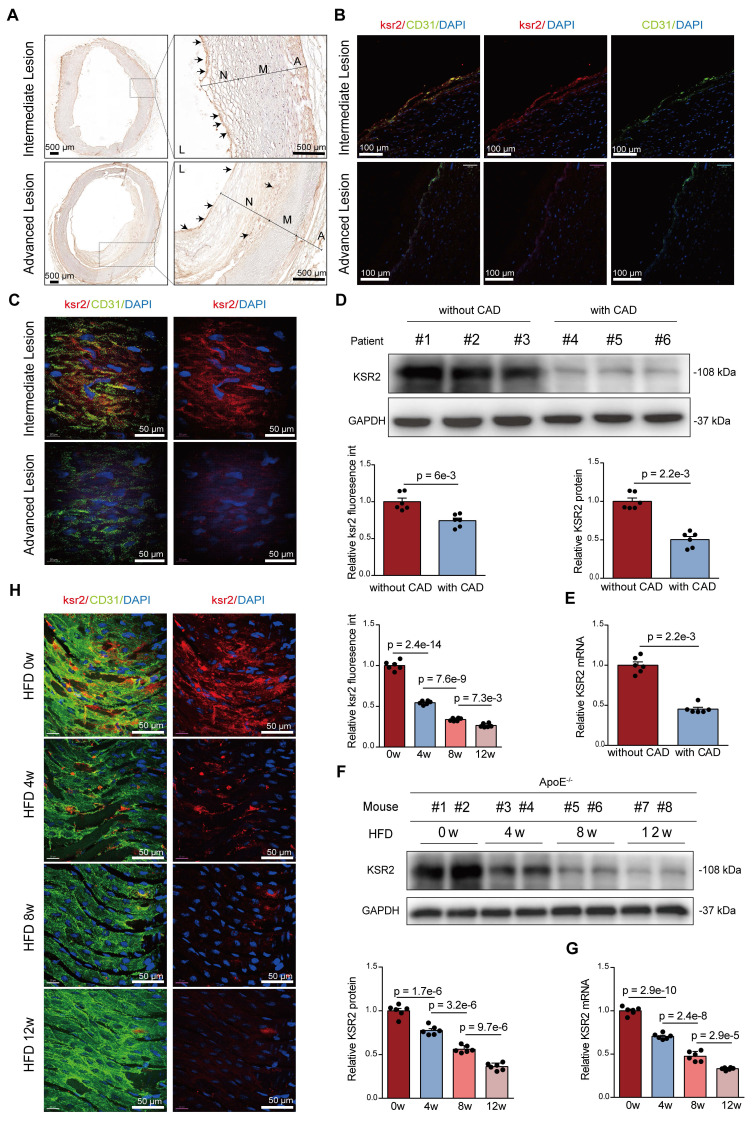
Expression of endothelial *KSR2* is reduced in plaques of both mice and humans. **A,** Immunohistochemical analysis of human coronary artery cryosections from intermediate and advanced lesions showing *KSR2* distribution within atherosclerotic plaques. Scale bars = 500 μm. **B,** Immunofluorescence staining of cryosections from intermediate and advanced coronary lesions showing endothelial *KSR2* localization. *KSR2* is shown in red, *CD31* in green, and nuclei (DAPI) in blue. Scale bars = 100 μm. **C,** *En face* immunofluorescence staining of intermediate and advanced coronary lesions showing quantitative changes in endothelial *KSR2* expression. *KSR2* is shown in red, *CD31* in green, and nuclei (DAPI) in blue. Scale bars = 50 μm. *n =* 6, 2-tailed unpaired Student's *t*-test. D and E, Western blot** (D)** and RT-qPCR **(E)** analyses of *KSR2* expression in coronary artery tissues from patients with coronary artery disease (CAD) and non-CAD controls. 2-tailed unpaired Mann-Whitney U test due to non-normal distribution. F and G, Western blot **(F)** and RT-qPCR **(G)** analyses showing temporal changes in *KSR2* expression in aortic tissues from mice fed a high-fat diet (HFD) for 0, 4, 8, and 12 weeks. *n =* 6, ordinary one-way ANOVA. **H,** *En face* immunofluorescence staining of the aortic endothelium showing dynamic changes in *KSR2* expression in mice fed a high-fat diet for 0, 4, 8, and 12 weeks. *KSR2* is shown in red, *CD31* in green, and nuclei (DAPI) in blue. Scale bars = 50 μm. *n =* 6, ordinary one-way ANOVA. L, lumen; N, neointima; M, media; A, adventitia.

We next investigated *KSR2* expression patterns in atherosclerotic plaques. Immunohistochemistry and immunofluorescence co-localization analyses of frozen sections from human coronary arteries revealed that *KSR2* was predominantly expressed in macrophages and endothelial cells, with minimal expression observed in smooth muscle cells (Figure 2A-B, Figure S2A-B). Notably, endothelial *KSR2* expression was significantly decreased in severely atherosclerotic coronary arteries compared to those with mild or moderate lesions, whereas *KSR2* levels in smooth muscle cells and macrophages remained largely unchanged. Consistently, *en face* immunofluorescence staining confirmed the downregulation of endothelial *KSR2* in advanced plaques (Figure 2C). In addition, western blot and RT-qPCR analyses demonstrated significantly reduced *KSR2* protein and mRNA levels in coronary artery tissues from clinically diagnosed CAD patients compared to non-CAD controls (Figure 2D-E), with baseline characteristics of the patient cohorts summarized in Table S1.

To assess whether similar *KSR2* expression patterns occur in murine atherosclerosis, we established a progressive atherosclerosis model in *Apoe*^-/-^ mice through high-fat diet (HFD) feeding for 0, 4, 8, and 12 weeks. Immunofluorescence co-localization analysis revealed that, consistent with human coronary specimens, *KSR2* was primarily localized in macrophages and endothelial cells within murine atherosclerotic plaques, with low expression detected in smooth muscle cells (Figure S2C). Western blot and RT-qPCR results further showed a time-dependent decrease in both *KSR2* protein and mRNA levels in the aorta with prolonged HFD feeding (Figure 2F-G). Importantly, primary aortic endothelial cells, smooth muscle cells, and peritoneal macrophages were isolated from each group of mice. Western blot analysis demonstrated a progressive reduction of *KSR2* expression in endothelial cells, while its levels remained relatively unchanged in smooth muscle cells and macrophages (Figure S2D-F). Notably, *en face* immunofluorescence staining confirmed the gradual decline of endothelial *KSR2* expression with increasing plaque severity (Figure 2H). Collectively, these findings suggest that vascular *KSR2*—particularly within endothelial cells—may play a critical role in the pathogenesis and progression of atherosclerosis.

### *KSR2* mitigates atherosclerosis progression via endothelial cell-autonomous manner

**Figure 3:**
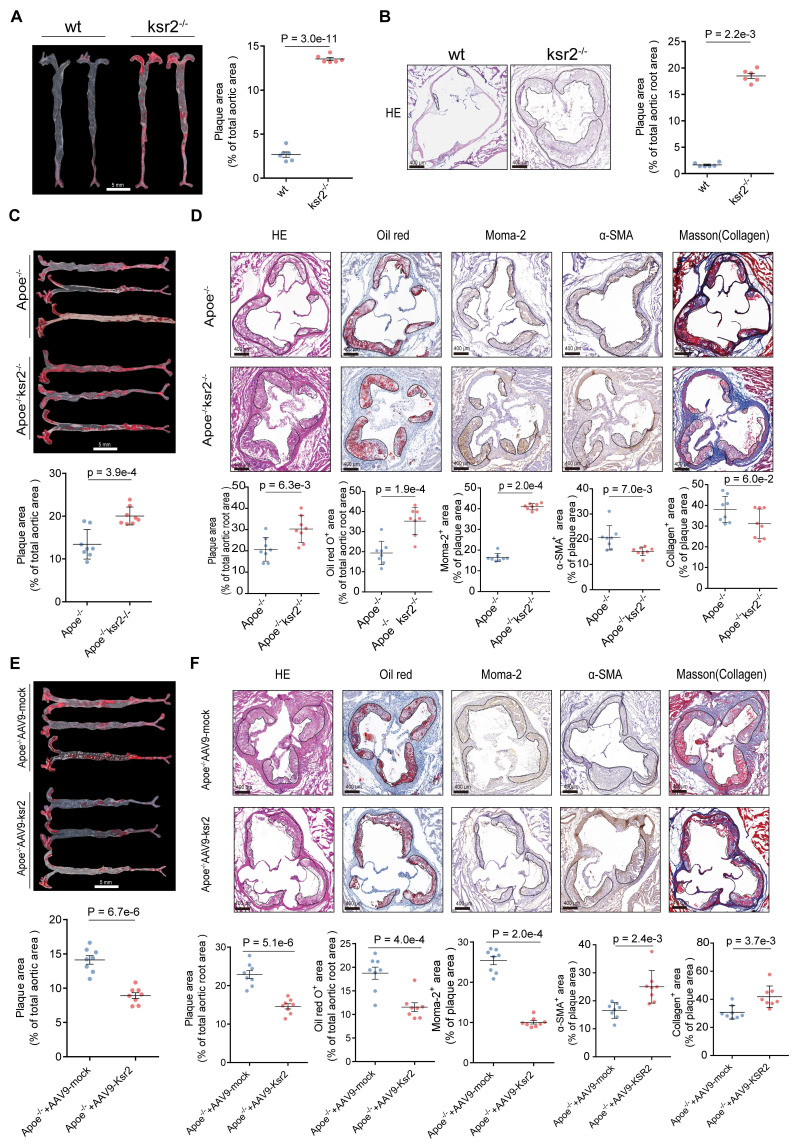
** Endothelial *KSR2* mitigates atherosclerosis progression. A.** Representative *en face* images of Oil Red O-stained aortas from wild-type and *KSR2*⁻/⁻ mice fed a high-fat diet ad libitum. Quantification of aortic lesion areas is shown. Scale bar = 5 mm. 2-tailed unpaired Mann-Whitney U test due to non-normally distributed data. **B.** Cross-sectional analysis of aortic roots from wild-type and *KSR2*⁻/⁻ mice stained with hematoxylin and eosin (H&E). Quantification of aortic lesion areas is shown. Scale bar = 400 μm. n = 6, 2-tailed unpaired Student's t-test.** C.** Representative *en face* images of Oil Red O-stained aortas from pair-fed *Apoe*⁻/⁻ and *Apoe*⁻/⁻*KSR2*⁻/⁻ mice on a high-fat diet. Quantification of aortic lesion areas is shown. Scale bar = 5 mm. n = 8, 2-tailed unpaired Student's t-test. **D.** Cross-sectional analysis of aortic roots from *Apoe*^-/-^ and *Apoe*^-/-^*KSR2*^-/-^ mice stained with hematoxylin and eosin (H&E), Oil Red O, *MOMA-2* and *α-SMA* immunohistochemistry (IHC), and Masson's trichrome staining. Scale bar = 400 μm. n = 8. Statistical analyses were performed using a 2-tailed unpaired Student's t-test for all results, except for *α-SMA* IHC staining, which was analyzed using a 2-tailed unpaired Mann-Whitney U-test due to non-normally distributed data. **E,** Representative *en face* images of Oil Red O-stained aortas from *Apoe*^-/-^ + AAV9-mock and *Apoe*^-/-^ + AAV9-*KSR2* mice. Scale bar = 5 mm. n = 8, 2-tailed unpaired Student's t-test.** F,** Cross-sectional analysis of aortic roots from *Apoe*^-/-^ + AAV9-mock and *Apoe*^-/-^ + AAV9-*KSR2* mice stained with H&E, Oil Red O, *MOMA-2* and *α-SMA* immunohistochemistry, and Masson's trichrome staining. Scale bar = 400 μm. n = 8, 2-tailed unpaired Student's t-test, except for Masson's trichrome staining, which was analyzed using the 2-tailed unpaired Mann-Whitney U test due to non-normally distributed data.

While previous studies using global *KSR2* knockout mice primarily focused on obesity and systemic metabolic phenotypes, the vascular implications remained unexplored. To address this, 8-week-old wild-type (WT) and *KSR2*^-/-^ mice were fed high-fat diet ad libitum for 12 weeks, the knockout strategy and efficiency of *KSR2* deletion are shown in Figure S3A-C. Consistent with prior reports, global *KSR2* deletion induced weight gain, and systemic metabolic dysregulation (Figure S3D-K). Oil Red O and H&E staining of the aorta revealed markedly increased plaque size in *KSR2*^-/-^ mice compared with wild-type controls following HFD feeding (Figure 3A-B).

Previous studies have demonstrated that the metabolic disorders observed in *KSR2*^-/-^ mice are primarily caused by hyperphagia 25. To further investigate whether *KSR2* contributes to atherosclerosis development in a cell-autonomous manner, we generated *KSR2* knockout mice on an *Apoe*^-/-^ background, then controlled the food intake of *Apoe*^-/-^*KSR2*^-/-^ mice to match that of the *Apoe*^-/-^ mice through pair feeding (PF). After 12 weeks of HFD feeding, no significant differences in serum lipid profiles (triglycerides, total cholesterol, LDL-C, and HDL-C), glucose levels, insulin levels or HOMA-IR index were observed between *Apoe*^-/-^*KSR2*^-/-^ and *Apoe*^-/-^ mice, except for body weight. *Apoe*^-/-^*KSR2*^-/-^ mice exhibited a slight increase in body weight compared to *Apoe*^-/-^ mice (Figure S4A-H). Notably, Oil Red O staining of the entire aorta revealed a notable increase in atherosclerotic lesion area in *Apoe*^-/-^*KSR2*^-/-^ mice compared to *Apoe*^-/-^ mice (Figure 3C). Cross-sectional analysis of the aortic roots further confirmed that *KSR2* knockout exacerbated plaque burden, as evidenced by increased lipid deposition, macrophage infiltration, and reduced vascular smooth muscle cell content and collagen accumulation, as assessed by H&E staining, Oil Red O staining, immunohistochemistry for *MOMA-2* and *α-SMA*, and Masson's trichrome staining (Figure 3D). Collectively, these results indicate that *KSR2* deficiency accelerates plaque progression independent of serum lipid and glucose levels.

To further investigate the role of endothelial *KSR2* in atherosclerosis, we administered AAV9-*ICAM2*-*KSR2* (AAV9-*KSR2*) via tail vein injection into *Apoe*^-/-^ mice, resulting in endothelial-specific overexpression of *KSR2*. *In vivo* imaging and aortic RT-qPCR confirmed successful *KSR2* overexpression in the endothelial cells of the mice (Figure S5A-B). Notably, endothelial-specific overexpression of *KSR2* had no significant effects on body weight, blood glucose, lipid profiles, serum insulin or HOMA-IR index (Figure S5C-J). Compared to *Apoe*^-/-^ + AAV9-*ICAM2*-mock (AAV9-mock) mice, *Apoe*^-/-^ + AAV9-*KSR2* mice exhibited significantly smaller atherosclerotic plaques, accompanied by reduced lipid deposition and macrophage infiltration, as well as increased vascular smooth muscle cell and collagen content (Figure 3E-F). These findings suggest that endothelial-specific *KSR2* overexpression reduces plaque burden and enhances plaque stability, highlighting a protective role for endothelial *KSR2* in atherosclerosis progression.

### *KSR2* mitigates atherosclerosis by inhibiting endothelial cell inflammation and apoptosis

**Figure 4:**
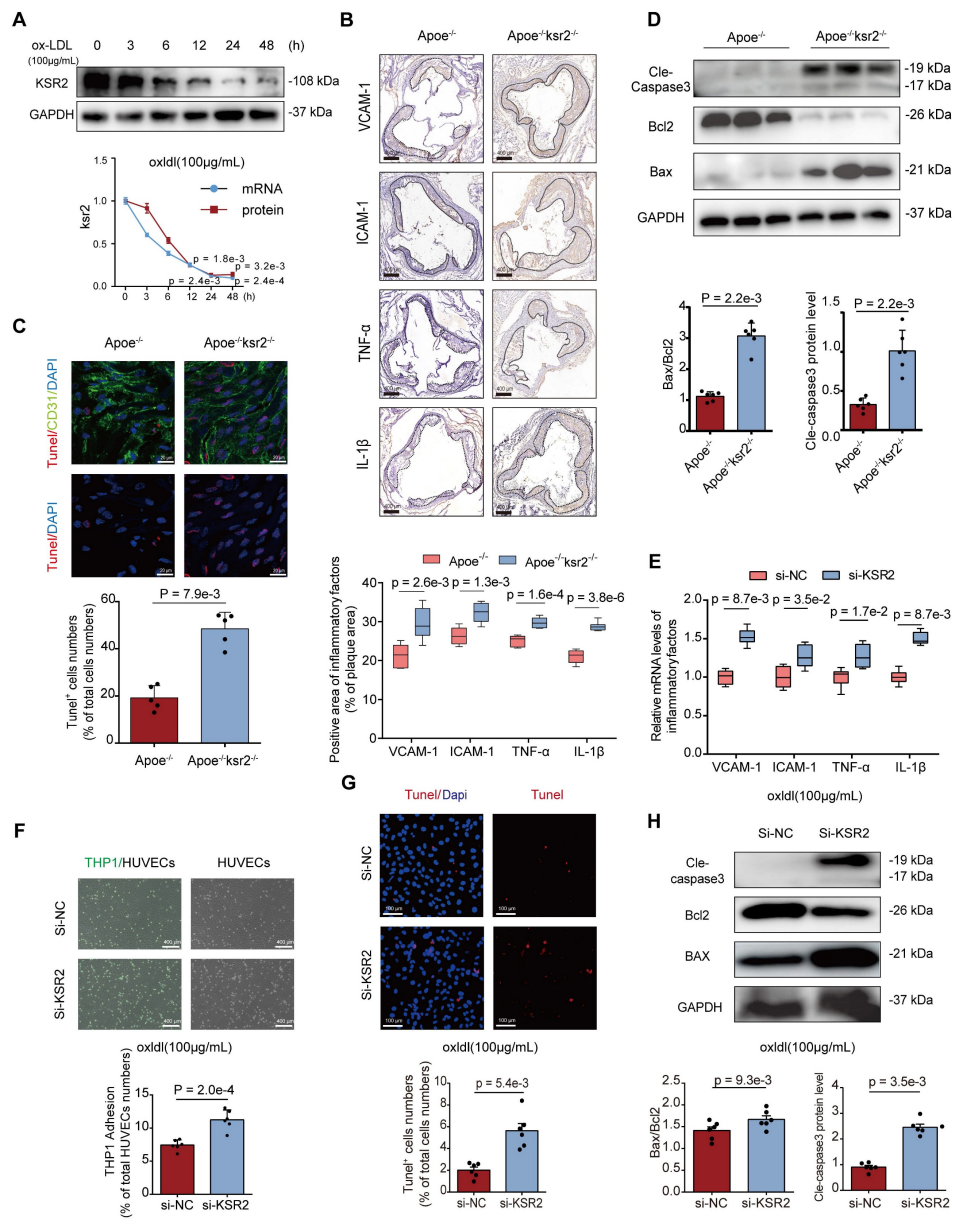
***KSR2* deficiency exacerbates atherosclerosis by promoting endothelial inflammation and apoptosis. A,** Western blot (WB) and RT-qPCR analyses were performed to assess changes in *KSR2* expression in HUVECs after time gradient stimulation with oxLDL (100 μg/mL). Relative values are compared to the 0h control group. n = 5, Kruskal-Wallis test with Dunn's multiple comparisons test due to small sample sizes. **B,** Immunohistochemical staining of *VCAM-1*,* ICAM-1*, *TNF-α*, and *IL-1β* in the aortic roots of *Apoe*^-/-^ and *Apoe*^-/-^*KSR2*^-/-^ mice. Scale bar = 400 μm. n = 6, 2-tailed unpaired Student's t-test. **C,** Representative *en face* TUNEL staining of endothelial cells in the aorta of *Apoe*^-/-^ and *Apoe*^-/-^*Ksr2*^-/-^ mice. TUNEL-positive cells are shown in red, *CD31* in green, and nuclei (DAPI) in blue. Scale bar = 20 μm. n = 5, 2-tailed unpaired Mann-Whitney U-test due to small sample sizes. **D,** Western blot analysis of cleaved caspase-3, *Bcl-2*, and *Bax* protein levels in aortic tissues from *Apoe*^-/-^ and *Apoe*^-/-^*Ksr2*^-/-^ mice. n = 6, 2-tailed unpaired Mann-Whitney U-test due to non-normally distributed data. **E-H,** HUVECs were transfected with control siRNA (si-NC) or *KSR2* siRNA (si-*KSR2*) for 48 h, followed by treatment with oxLDL (100 μg/mL) for 24 h. **(E)** RT-qPCR was used to measure the mRNA levels of *VCAM-1*,* ICAM-1*, *TNF-α*, and *IL-1β*. n = 6, 2-tailed Mann-Whitney U tests (multiple t-test framework, one per gene) with Bonferroni adjustment to control the family-wise error rate. **(F)** THP-1 adhesion assay assessing the adhesive capacity of HUVECs following *KSR2* knockdown. Results are presented as the percentage of endothelial cells with adherent THP-1 cells. Scale bar = 400 μm. n = 6, 2-tailed unpaired Student's t-test.** (G)** TUNEL staining analysis of apoptosis in HUVECs following *KSR2* knockdown. Results are presented as the percentage of TUNEL-positive cells. Scale bar = 100 μm. n = 6, 2-tailed unpaired Mann-Whitney U test due to non-normally distributed data. **(H)** WB analysis was used to assess the protein levels of cleaved caspase-3, *Bcl-2*, and *Bax*. n = 6, 2-tailed unpaired Student's t-test.

To validate the function of *KSR2 in vitro*, we initially subjected HUVECs to time-gradient stimulations with oxLDL and palmitic acid (PA). RT-qPCR and WB analysis revealed that both oxLDL and PA treatments significantly reduced the mRNA and protein levels of *KSR2* in HUVECs (Figure 4A, Figure S6). Stable *KSR2*-overexpressing HUVEC lines were generated via lentiviral transduction (Figure S7A). To investigate the underlying mechanisms of *KSR2* action, RNA sequencing was performed on *KSR2*-overexpressing and control HUVECs, identifying 535 upregulated and 444 downregulated genes (fold change ≥1.2; P < 0.05). Gene set enrichment analysis revealed that the differentially expressed genes were significantly enriched in pathways related to metabolism, apoptosis, inflammation, and cell adhesion (Figure S7B-D). Given that endothelial inflammation and apoptosis play pivotal roles in the initiation and progression of atherosclerosis 26, we next investigated the effects of *KSR2* on endothelial inflammation and apoptosis both *in vivo* and *in vitro*.

To assess the inflammatory response in mouse plaques, we performed immunohistochemical staining analysis. Compared to *Apoe*^-/-^ mice, *Apoe*^-/-^*KSR2*^-/-^ mice exhibited significantly higher expression levels of* ICAM-1*, *VCAM-1*, *TNF-α*, and *IL-1β* in atherosclerotic plaques (Figure 4B). To investigate the impact of *KSR2* on endothelial cell apoptosis, *en face* immunofluorescence staining for *CD31* and TUNEL was performed on the aortic endothelium. The results revealed a significantly higher proportion of TUNEL-positive cells in the aortic endothelium of *Apoe*^-/-^*KSR2*^-/-^ mice compared to *Apoe*^-/-^ mice (Figure 4C). Furthermore, western blot analysis showed a marked increase in *cleaved-caspase3* and *Bax* protein levels, while *Bcl-2* expression was significantly reduced in *Apoe*^-/-^*KSR2*^-/-^ mice compared to *Apoe*^-/-^ mice (Figure 4D).

To further investigate the role of *KSR2* in endothelial function, we used *KSR2*-specific siRNA to knock down its expression in HUVECs, with knockdown efficiency confirmed by RT-qPCR (Figure S8). For subsequent experiments, a combination of siRNA-2 and siRNA-3 was used to enhance knockdown efficiency. Under oxLDL stimulation, *KSR2* knockdown significantly upregulated the mRNA expression of *VCAM1*, *ICAM1*, *TNF-α*, and *IL-1β* in HUVECs (Figure 4E). Consistently, THP-1 adhesion assays demonstrated increased monocyte adhesion to HUVECs following *KSR2* knockdown (Figure 4F). In parallel, knockdown of *KSR2* led to a higher proportion of TUNEL-positive endothelial cells (Figure 4G), accompanied by elevated expression of *Bax* and *cleaved caspase-3*, and decreased *Bcl-2* protein levels (Figure 4H).

Similarly, immunohistochemistry, TUNEL staining, and WB analyses demonstrated that *Apoe*^-/-^ + AAV9-*KSR2* mice exhibited significantly reduced intra-plaque inflammation and endothelial cell apoptosis compared to *Apoe*^-/-^ + AAV9-mock controls (Figure S9A-C). Consistent with these findings, *in vitro* RT-qPCR, THP-1 adhesion assays, TUNEL staining, and WB further confirmed that *KSR2* overexpression markedly attenuated inflammation and apoptosis in HUVECs (Figure S9D-G). Taken together, these findings suggest that *KSR2* mitigates the progression of atherosclerosis by suppressing endothelial inflammation and apoptosis.

### *KSR2* suppresses endothelial cell inflammation and apoptosis by maintaining glycolytic balance

We next sought to further elucidate how *KSR2* regulates endothelial inflammation and apoptosis. Transcriptomic analysis revealed that *KSR2* significantly influences endothelial cell metabolism, consistent with previous studies identifying *KSR2* as a gene closely associated with systemic metabolic regulation. Based on these findings, we hypothesize that *KSR2* exerts its anti-inflammatory and anti-apoptotic effects by modulating endothelial metabolism. Endothelial metabolism plays a critical role in the pathogenesis of various cardiovascular diseases, with aerobic glycolysis contributing to 75-85% of the total ATP production in endothelial cells 27. Therefore, we further investigated the relationship between *KSR2* and endothelial glycolysis.

**Figure 5:**
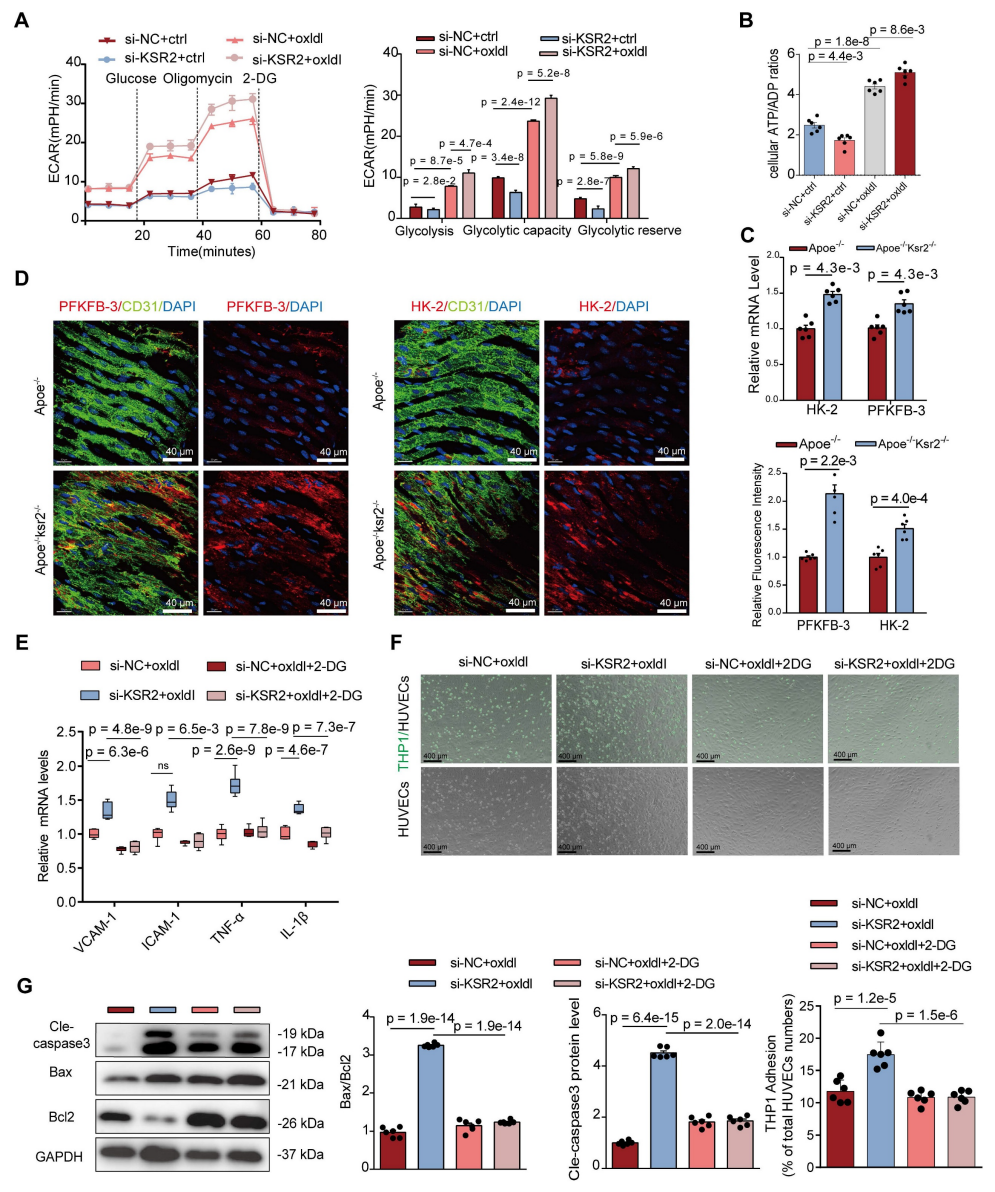
***KSR2* suppresses endothelial inflammation and apoptosis by maintaining glycolytic homeostasis. A and B,** HUVECs transfected with si-NC or si-*KSR2* for 48 h were treated with oxLDL (100 μg/mL) or control for 24 h.** (A)** The extracellular acidification rate (ECAR) profile was used to evaluate the glycolytic function of HUVECs. Vertical lines indicate the addition of glucose (10 mM), oligomycin (1 μM), and 2-DG (50 mM). n = 10 for each treatment group, ordinary one-way ANOVA. **(B)** The cellular ATP/ADP ratio was determined to evaluate energy metabolic shifts in HUVECs. n = 6, ordinary one-way ANOVA. **C,** RT-qPCR analysis of *HK2* and *PFKFB3* mRNA levels in aortic tissues from *Apoe*⁻^/^⁻ and *Apoe*⁻^/^⁻*KSR2*⁻^/^⁻ mice. n = 6, 2-tailed Mann-Whitney U tests (multiple t-test framework, one per gene) with Bonferroni adjustment to control the family-wise error rate. **D,** Representative *en face* immunofluorescence staining of *PFKFB3* and *HK-2* in the endothelial cells of the aorta from *Apoe*^-/-^ and *Apoe*^-/-^*Ksr2*^-/-^ mice. *PFKFB3* or *HK-2* is shown in red, *CD31* in green, and DAPI in blue. Scale bar = 40 μm. n = 6. *PFKFB3* results were analyzed using the 2-tailed unpaired Mann-Whitney U test due to non-normally distributed data. *HK-2* results were analyzed using the 2-tailed unpaired Student's t-test. **E, F** and** G,** HUVECs transfected with si-NC or si-*KSR2* for 48 h were treated with oxLDL (100 μg/mL) + 2-DG (10 mM) or vehicle for 24 h.** (E)** RT-qPCR was used to measure the mRNA levels of *VCAM-1*,* ICAM-1*, *TNF-α*, and *IL-1β*. n = 6, ordinary one-way ANOVA. except for* ICAM-1*, which was analyzed using the Kruskal-Wallis test with Dunn's multiple comparisons test due to non-normally distributed data. **(F)** THP-1 adhesion assay assessing the adhesive capacity of HUVECs. Results are presented as the percentage of endothelial cells with adherent THP-1 cells. Scale bar = 400 μm. n = 6, ordinary one-way ANOVA. **(G)** WB analysis was performed to measure the protein levels of cleaved caspase-3, *Bcl-2*, and *Bax*. n = 6, ordinary one-way ANOVA.

First, we assessed the relative expression of glycolysis-related genes in HUVECs. Interestingly, *KSR2* overexpression modestly increased the mRNA levels of *HK2* and *PFKFB3* under resting conditions, yet significantly suppressed the oxLDL-induced upregulation of these genes (Figure S10A). To further investigate glycolytic metabolism, we performed Seahorse Extracellular Flux analysis to measure the extracellular acidification rate (ECAR) in HUVECs. Under resting conditions, the results showed a slight increase in glycolysis in *KSR2*-overexpressing HUVECs compared to controls. However, *KSR2* overexpression prominently inhibited the glycolytic increase induced by oxLDL stimulation (Figure S10B). In contrast, when *KSR2* expression was inhibited in HUVECs, the ECAR results showed a mild decrease in glycolysis under resting conditions, while oxLDL stimulation led to a further increase in endothelial glycolysis compared to controls (Figure 5A). Given that endothelial cells are largely glycolytic, we next quantified the intracellular ATP/ADP ratio. This functional assessment further corroborates the conclusion that *KSR2* is a key regulator of endothelial cell glycolysis (Figure S10C, Figure 5B).

Next, we examined the impact of *KSR2* on endothelial cell glycolysis *in vivo*. In *Apoe*^-/-^*KSR2*^-/-^ mice, the mRNA levels of *HK2* and *PFKFB3* in the aorta were significantly elevated compared to *Apoe*^-/-^ mice (Figure 5C). *En face* staining of the aortic endothelium further revealed increased protein levels of *HK2* and *PFKFB3* in the *Apoe*^-/-^*KSR2*^-/-^ mice (Figure 5D). In contrast, in *Apoe*^-/-^ + AAV9-*KSR2* mice, the mRNA levels of *HK2* and *PFKFB3* were significantly reduced compared to *Apoe*^-/-^ + AAV9-mock mice (Figure S10D), and *en face* staining also showed corresponding decreases in the protein levels of *HK2* and *PFKFB3* in the endothelial cells of *Apoe*^-/-^ + AAV9-*KSR2* mice (Figure S10E). Together, these findings strongly suggest that *KSR2* plays a crucial role in regulating the balance of endothelial cell glycolysis.

Previous studies have suggested that enhanced glycolysis in endothelial cells can promote the development of atherosclerosis by activating inflammation 28, 29. Therefore, we investigated whether the protective effect of endothelial *KSR2* against atherosclerosis is mediated through glycolysis. To suppress *KSR2* expression and glycolytic capacity in HUVECs, we used si-*KSR2* and 2-DG, respectively. RT-qPCR results revealed that under oxLDL stimulation, 2-DG effectively counteracted the increase in mRNA levels of *VCAM1*, *ICAM1*, *IL-1β*, and *TNF-α* induced by si-*KSR2* (Figure 5E). Furthermore, THP-1 adhesion assays revealed that under oxLDL stimulation, the inhibition of endothelial cell glycolysis by 2-DG significantly reduced the increased THP-1 adhesion induced by si-*KSR2* (Figure 5F). Additionally, western blot analysis demonstrated that 2-DG could restore the elevated protein levels of *Bax* and cleaved caspase-3, as well as the reduced *Bcl-2* levels induced by si-*KSR2* (Figure 5G). These results suggest that the protective role of *KSR2* in endothelial cells, particularly against inflammation and apoptosis, is mediated through the glycolytic pathway.

### *KSR2* inhibits K48-ubiquitin-proteasomal degradation of *AMPKα1*, activating the *AMPK* signaling pathway

*KSR2* plays a pivotal role in maintaining glycolytic homeostasis in endothelial cells, which prompted us to consider the involvement of the *AMPK* signaling pathway. As a crucial energy sensor, *AMPK* monitors cellular energy status and maintains metabolic balance 30, 31. Its involvement in metabolic regulation is multifaceted, with studies reporting both inhibitory and stimulatory effects on aerobic glycolysis 32, 33. Previous researches have demonstrated that *KSR2* interacts with *AMPK*, activating the *AMPK* signaling pathway and modulating downstream metabolic processes 13, 18. We therefore further investigated whether the protective effects of *KSR2* on endothelial cells are mediated through the *AMPK* signaling pathway.

**Figure 6:**
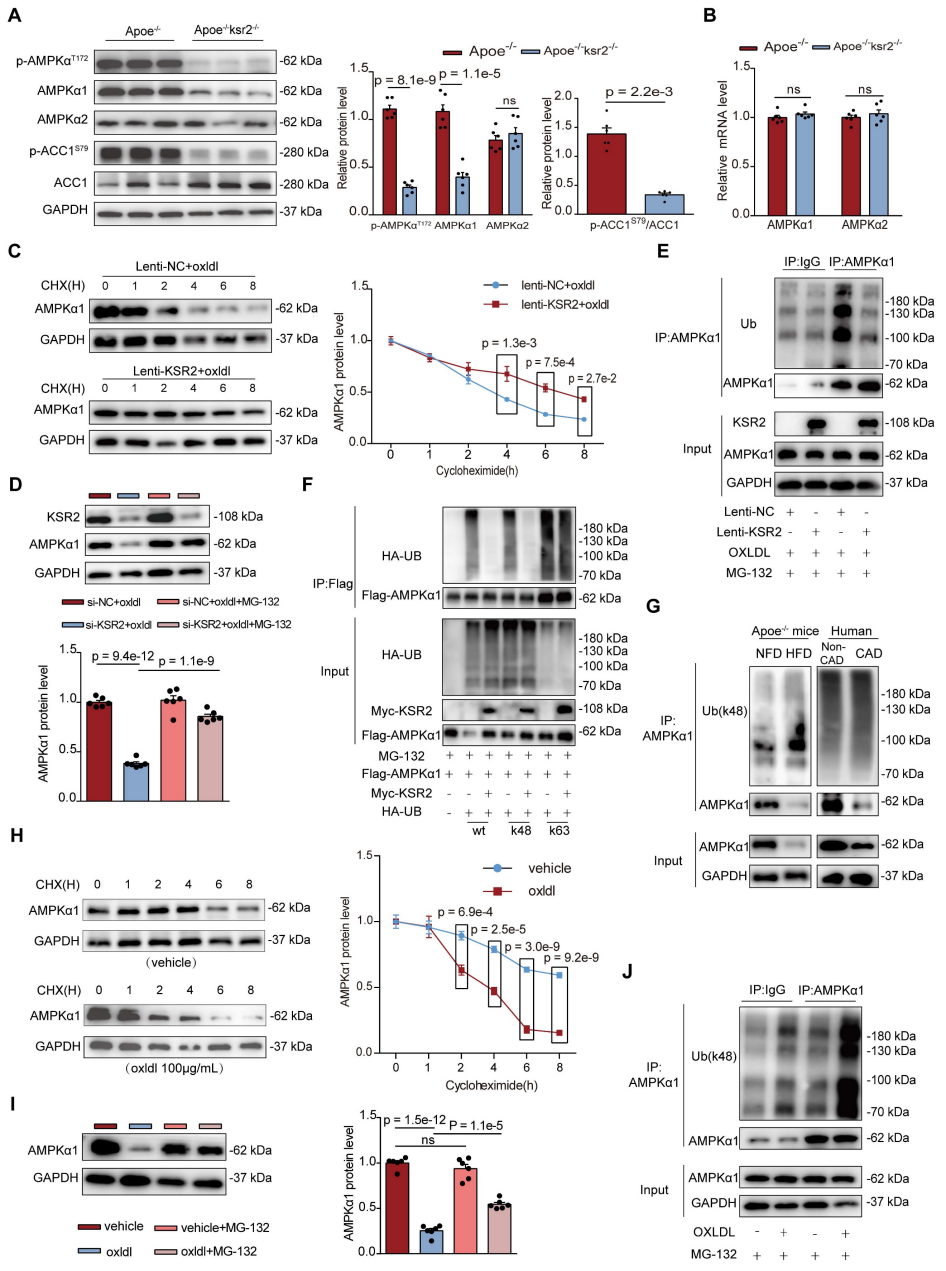
***KSR2* inhibits K48-linked ubiquitin-proteasomal degradation of *AMPKα1*, thereby activating the *AMPK* signaling pathway. A,** Western blot (WB) was used to assess the levels of p-*AMPK*α^T172^, *AMPKα1*,* AMPKα2*, p-*ACC1*^S79^, and *ACC1* proteins in the aortic tissue of *Apoe*^-/-^ and *Apoe*^-/-^*Ksr2*^-/-^ mice. n = 6. All results were analyzed using the 2-tailed unpaired Student's t-test, except for p-*ACC1*^S79^/*ACC1*, which was analyzed using the 2-tailed unpaired Mann-Whitney U test due to non-normally distributed data. **B,** RT-qPCR was used to investigate the mRNA levels of *AMPKα1* and* AMPKα2* in the aortic tissue of *Apoe*^-/-^ and *Apoe*^-/-^*Ksr2*^-/-^ mice. n = 6, 2-tailed unpaired Student's t-test. **C,** Lentiviral stable transfection of HUVECs with lenti-NC or lenti-*KSR2* was followed by treatment with oxLDL (100 μg/mL) for 24 h, then cycloheximide (CHX, 50 μg/mL) treatment for 0, 1, 2, 4, 6, and 8 h. WB analysis was performed to assess *AMPKα1* protein levels. n = 5, 2-way ANOVA.** D,** HUVECs were transfected with control siRNA (si-NC) or *KSR2* siRNA (si-*KSR2*) for 48 h, followed by pre-treatment with MG132 (10 μM) and subsequent oxLDL (100 μg/mL) stimulation for 24 h. WB analysis was performed to measure *AMPKα1* protein levels. n = 6, ordinary one-way ANOVA.** E,** Lentiviral stable transfection of HUVECs with lenti-NC or lenti-*KSR2* was followed by treatment with MG132 (10 μM) and oxLDL (100 μg/mL) for 24 h. Co-immunoprecipitation (Co-IP) and immunoblotting were performed to examine the total ubiquitination levels of endogenous *AMPKα1* via immunoprecipitation of *AMPKα1* or control *IgG* antibodies.** F,** HEK293T cells were transfected with myc-*KSR2*, Flag-*AMPKα1*, HA-ubiquitin, HA-UB(K48) (lysine 48-specific mutant), HA-UB(K63) (lysine 63-specific mutant), and appropriate control plasmids for 48 h, then treated with MG132 (10 μM) for 24 h. Co-IP and immunoblotting were performed to test the total, K48-linked, and K63-linked ubiquitination of exogenous *AMPKα1* via immunoprecipitation of Flag-tagged *AMPKα1*. **G,** Co-immunoprecipitation (Co-IP) and immunoblotting of endogenous *AMPKα1* ubiquitination (K48) levels were performed in the aortic tissue of *Apoe*^-/-^ mice fed a normal fat diet (NFD) or high-fat diet (HFD) (left), as well as in coronary artery tissues from non-CAD (non-coronary artery disease) and CAD (coronary artery disease) patients. **H,** HUVECs were treated with oxLDL (100 μg/mL) or vehicle for 24 h, then treated with CHX (50 μg/mL) for 0, 1, 2, 4, 6, and 8 h. WB analysis was performed to assess the changes in *AMPKα1* protein levels. n = 6, 2-way ANOVA.** I,** HUVECs were pre-treated with or without MG132 (10 μM), then stimulated with oxLDL (100 μg/mL) for 24 h. WB analysis was performed to measure *AMPKα1* protein levels. n = 6, ordinary one-way ANOVA.** J,** HUVECs were pre-treated with MG132 (10 μM) and then stimulated with oxLDL (100 μg/mL) or vehicle for 24 h. Co-IP and immunoblotting were performed to test the K48-linked ubiquitination levels of endogenous *AMPKα1* in HUVECs via immunoprecipitation of *AMPKα1* or control *IgG* antibodies. ns, not significant; UB: ubiquitination; *IgG*: immunoglobulin G.

In *Apoe*^-/-^*KSR2*^-/-^ mice, the protein levels p-*AMPKα*^T172^, p-*ACC1*^s79^ in the aorta were significantly reduced compared to *Apoe*^-/-^ mice (p-*AMPKα*^T172^, 1.11 ± 0.10 vs 0.29 ± 0.06; cohen's D = 9.94). Surprisingly, the protein level of *AMPKα1* was markedly reduced (1.09 ± 0.17 vs 0.40 ± 0.12; cohen's D = 4.69, Figure 6A), while no significant difference in *AMPKα1* mRNA expression was observed between the two groups. Moreover, both the mRNA and protein levels of* AMPKα2* remained unchanged (Figure 6B). Similarly, compared to *Apoe*^-/-^ + AAV9-mock mice, there was a significant increase in protein levels of *AMPKα1*(0.62 ± 0.25 vs 1.00 ± 0.11; Cohen's D = -1.97) and p-*AMPK*α*^T172^* (0.36 ± 0.09 vs 1.07 ± 0.21; Cohen's D = -4.37), along with elevated levels of p-*ACC1*^s79^ in *Apoe*^-/-^ + AAV9-*KSR2* mice (Figure S11A). However,* AMPKα2* protein levels and *AMPKα1*,* AMPKα2* mRNA levels remained unchanged (Figure S11B). Western blot analysis revealed that the increase in p-*AMPK*α^T172^ was more pronounced than that of total *AMPKα1*, suggesting that *KSR2* not only enhances *AMPK*α phosphorylation, as previously reported 18, 19, 34, but also directly regulates *AMPKα1* protein abundance—a mechanism not described in earlier studies. Therefore, we next sought to elucidate the precise mechanism by which *KSR2* regulates *AMPKα1* protein abundance. Cycloheximide (CHX) chase assays demonstrated that *KSR2* overexpression markedly reduced the degradation rate of *AMPKα1* protein (Figure 6C), indicating that *KSR2* inhibits *AMPKα1* protein turnover.

Further mechanistic insights revealed that the proteasome inhibitor MG132 restored *AMPKα1* protein levels in *KSR2*-deficient HUVECs, whereas the autophagy inhibitor 3-MA and lysosome inhibitor chloroquine (CQ) had no effect (Figure 6D, Figure S12A-B). These results indicate that *KSR2* prevents *AMPKα1* degradation via the ubiquitin-proteasomal pathway. We next investigated the effects of *KSR2* on endogenous *AMPKα1* ubiquitination in HUVECs. Co-IP assays revealed that *KSR2* overexpression significantly reduced the overall ubiquitination levels of *AMPKα1* in HUVECs (Figure 6E). To confirm these findings, HEK293T cells were co-transfected with exogenous *AMPKα1*, *KSR2*, and ubiquitin plasmids specific for K48, K63, M0, K6, K11, K27, K29 or K33 linkages, the result showed that *KSR2* significantly reduced K48-linked ubiquitination of *AMPKα1* in HEK293T cells, without affecting any other linkage type (Figure 6F, Figure S13). In summary, these results demonstrate that *KSR2* stabilizes *AMPKα1* protein levels by suppressing K48-linked ubiquitin-proteasomal degradation, thereby activating the *AMPK* signaling pathway.

Previous studies on the regulation of the *AMPK* signaling pathway have primarily focused on mechanisms related to its phosphorylation. In contrast, the ubiquitin-proteasome-mediated degradation of *AMPK* has been rarely reported. Peng Jiang *et al.* 35 reported that MG53 mediates K48-linked ubiquitin-proteasome degradation of* AMPKα2* in skeletal muscle of type 2 diabetes and obesity models, suppressing *AMPK* signaling pathway activation. Whether a similar regulatory mechanism exists in atherosclerosis models remains unclear. To investigate this, we performed a series of *in vivo* and *in vitro* experiments. Co-IP results revealed a marked increase in K48-linked ubiquitination of endogenous *AMPKα1* protein in coronary artery tissues from patients clinically diagnosed with CAD compared to those without CAD. This observation was further validated in HFD-fed *Apoe*^-/-^ mice (Figure 6G). In HUVECs, CHX chase assays demonstrated that oxLDL treatment accelerated the degradation of *AMPKα1* protein (Figure 6H), while MG132, a proteasome inhibitor, partially restored *AMPKα1* protein levels under oxLDL stimulation, as confirmed by WB analysis. Notably, MG132 treatment under basal conditions did not further increase *AMPKα1* protein levels, suggesting that *AMPKα1* degradation is largely independent of the ubiquitin-proteasome pathway under basal conditions (Figure 6I). Furthermore, Co-IP assays revealed that oxLDL stimulation markedly increased endogenous K48-linked ubiquitination of *AMPKα1* in HUVECs, whereas under basal conditions, K48-linked ubiquitination of *AMPKα1* was comparable to *IgG* controls, consistent with the MG132 results (Figure 6J). In conclusion, our findings provide the evidence that endothelial cell *AMPKα1* undergoes a non-canonical ubiquitin-proteasome regulatory mechanism in the context of atherosclerosis. Moreover, *KSR2* modulates *AMPKα1* activity through this pathway.

### *KSR2* competitively binds to the K52 site of *AMPKα1*, inhibiting the *CRL4A^CRBN^* E3 ubiquitin ligase complex-mediated K48 ubiquitination and proteasomal degradation of *AMPKα1*

**Figure 7:**
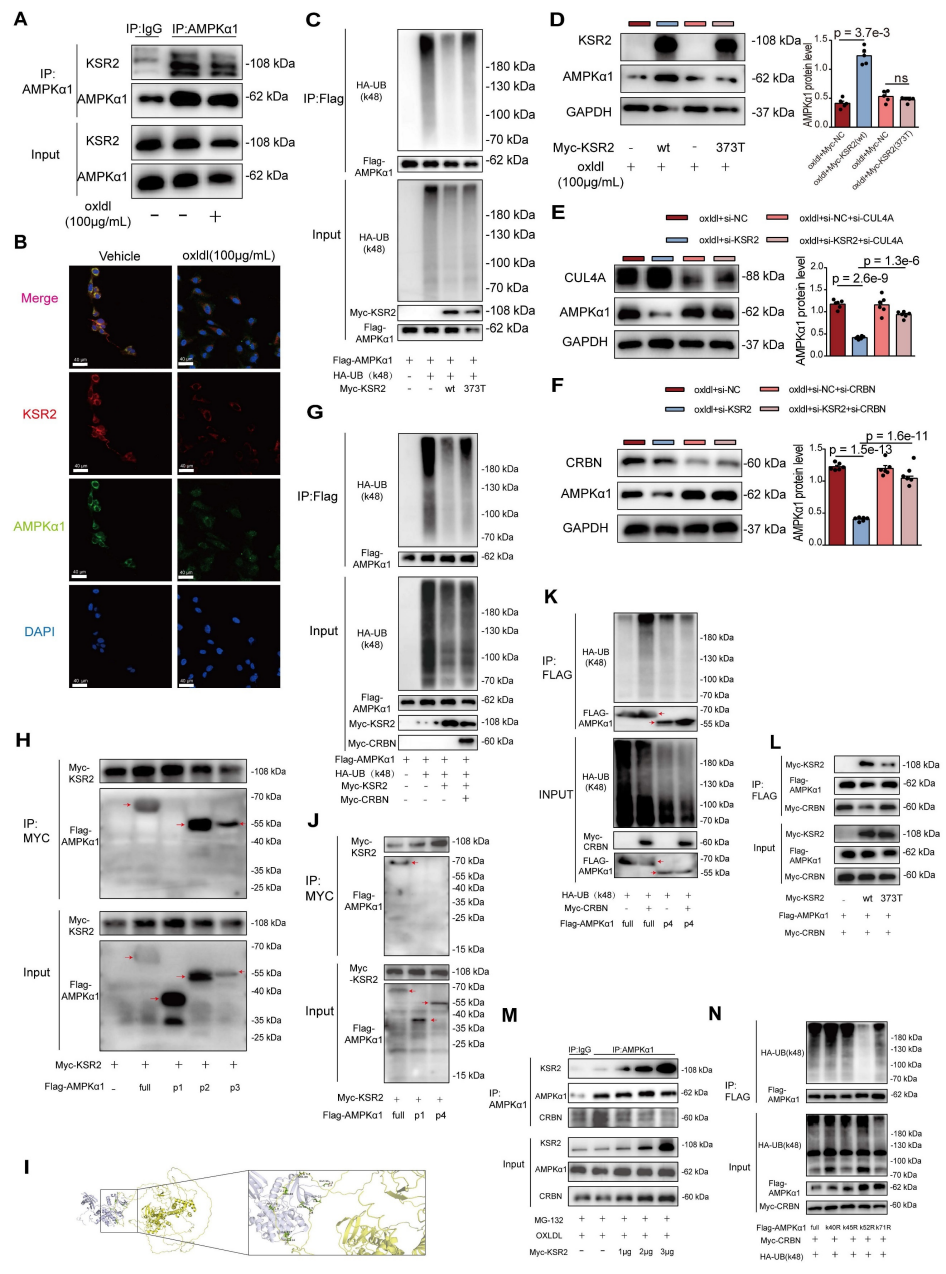
***KSR2* competitively binds to the K52 site of *AMPKα1*, inhibiting *CRL4A^CRBN^* E3 ubiquitin ligase complex-mediated K48-linked ubiquitination and proteasomal degradation of *AMPKα1*. A,** Co-IP and immunoblotting were performed to assess the interaction between endogenous *KSR2* and *AMPKα1* in HUVECs stimulated with oxLDL (100 μg/mL) for 24 h, using immunoprecipitation of *AMPKα1* or control *IgG* antibodies. **B,** Immunofluorescence co-localization analysis was used to examine the interaction between endogenous *KSR2* and *AMPKα1* after oxLDL stimulation. Scale bar = 40 μm.** C,** HEK293T cells were transfected with myc-*KSR2*(wt) or myc-*KSR2*(373T), Flag-*AMPKα1*, and HA-UB(K48) (lysine 48-specific mutant). Co-IP and immunoblotting were performed to test the K48-linked ubiquitination of exogenous *AMPKα1*. **D,** HUVECs were transfected with myc-*KSR2*(wt) or myc-*KSR2*(373T) for 48 h, followed by oxLDL (100 μg/mL) treatment for 24 h. WB was used to measure *AMPKα1* protein levels. n = 5, Kruskal-Wallis test with Dunn's multiple comparisons test due to small sample sizes.** E** and** F,** HUVECs were transfected with **(E)** *CUL4A* siRNA (si-*CUL4A*) or **(F)** *CRBN* siRNA (si-*CRBN*) along with *KSR2* siRNA for 48 h, followed by oxLDL (100 μg/mL) for 24 h. WB was used to assess *AMPKα1* protein levels. n = 6, ordinary one-way ANOVA.** G.** HEK293T cells were transfected with myc-*KSR2*, Flag-*AMPKα1*, HA-UB(K48), and myc-*CRBN* for 48 h. Co-IP and immunoblotting were performed to test K48-linked ubiquitination of exogenous *AMPKα1*.** H.** HEK293T cells were transfected with myc-*KSR2* and full-length or truncated forms of Flag-*AMPKα1*(P1 (Δ1-294 aa), P2 (Δ295-396 aa), and P3 (Δ397-574 aa)) for 48 h. Co-IP and immunoblotting were performed to examine the interaction between *KSR2* and different *AMPKα1* truncates.** I,** HADDOCK was used to predict the interaction sites and key regions between *KSR2* (AF-Q6VAB6-F1) and *AMPKα1* (6C9H, 22-559aa). The active residues were predicted using the WHISCY server, and the resulting model was visualized using PyMOL. Note that the *AMPKα1* protein structure (6C9H) is incomplete; therefore, the predicted *AMPKα1* sites in this model correspond to actual sites (Q13131) with an additional 9 residues.** J,** HEK293T cells were transfected with myc-*KSR2* and full-length or truncated Flag-*AMPKα1*(P1 (Δ1-294 aa), p4 (Δ1-123 aa)) for 48 h. Co-IP and immunoblotting were used to examine the interaction between *KSR2* and *AMPKα1* truncates.** K,** HEK293T cells were transfected with myc-*CRBN*, HA-UB(K48), and full-length or truncated Flag-*AMPKα1* p4 (Δ1-123 aa) for 48 h. Co-IP and immunoblotting were performed to explore the effect of *CRBN* on K48 ubiquitination of *AMPKα1* truncates. **L,** HEK293T cells were transfected with myc-*KSR2* (wt) or myc-*KSR2*(373T), Flag-*AMPKα1*, and myc-*CRBN* for 48 h. Co-IP and immunoblotting were used to investigate the effect of *KSR2* binding to *AMPKα1* on *CRBN* interaction.** M,** HUVECs were transfected with increasing amounts of myc-*KSR2* plasmid (0, 1, 2, 3 μg) for 48 h, pre-treated with MG132 (10 μM), and stimulated with oxLDL (100 μg/mL) for 24 h. Co-IP and immunoblotting were performed to detect the interaction between endogenous *AMPKα1* and *CRBN*.** N,** HEK293T cells were transfected with myc-*CRBN*, HA-UB(K48), and full-length or mutant Flag-*AMPKα1* (K40R, K45R, K52R, K71R) for 48 h. Co-IP and immunoblotting were used to analyze the K48 ubiquitination of exogenous Flag-*AMPKα1*. ns, not significant.

We next investigated whether the stabilization of *AMPKα1* protein by *KSR2* is mediated through direct interaction. Co-IP (Figure 7A) and immunofluorescence analyses (Figure 7B) confirmed that *KSR2* interacts with *AMPKα1* in HUVECs, and this interaction was attenuated upon oxLDL stimulation. Previous studies have identified the amino acid at position 373 (A373) as a critical site for the interaction between *KSR2* and *AMPKα1* 13. To further explore this, HUVECs were transfected with a *KSR2* mutant plasmid (myc-*KSR2* 373T). Co-IP results confirmed that the inhibition of K48-linked ubiquitination of *AMPKα1* by myc-*KSR2* 373T was significantly attenuated (Figure 7C). Importantly, Western blot analysis revealed that, compared to the wild-type myc-*KSR2* plasmid, overexpression of myc-*KSR2* 373T resulted in a substantial reduction in the stabilization of *AMPKα1* protein levels (Figure 7D). In conclusion, *KSR2* stabilizes *AMPKα1* protein in endothelial cells through direct interaction, thereby inhibiting its K48-linked ubiquitin-proteasomal degradation and activating the *AMPK* signaling pathway.

*KSR2*, as a scaffold protein, lacks intrinsic ubiquitin ligase or deubiquitinase activity. We hypothesized that *KSR2* might regulate *AMPKα1* ubiquitination and subsequent proteasomal degradation via a specific E3 ubiquitin ligase. Seven E3 ubiquitin ligases have been reported to mediate *AMPKα1* degradation through the ubiquitin-proteasome pathway: *MKRN1* 36, *RMND5A* 37, MAGE-A3/6/*TRIM28* 38, *PEDF* 39, *CRL4A^CRBN^* 40, *CRL4A^FBXo48^* 41, and *RNF44* 42. We designed siRNAs targeting the seven E3 ubiquitin ligases mentioned above, western blot analysis showed that si-*CRL4A* or si-*CRBN* markedly increased *AMPKα1* protein levels in *KSR2*-depleted HUVECs (Figure 7E-F, Figure S14A-F). Based on these findings, we hypothesize that *KSR2* inhibits the E3 ubiquitin ligase *CRL4A^CRBN^*-mediated degradation of endothelial cell *AMPKα1*.

To further validate this, we utilized the *CRBN* protein degrader TD165 43, based on PROTAC technology, and lenalidomide 44, a *CRBN* protein modulator. Western blot results showed that pre-treatment with TD165 significantly prevented the reduction of *AMPKα1* protein levels induced by si-*KSR2* in endothelial cells, whereas lenalidomide treatment had no significant effect on *AMPKα1* protein levels (Figure S14G-H). These results suggest that the E3 ubiquitin ligase *CRL4A^CRBN^* mediates *AMPKα1* degradation in endothelial cells via a lenalidomide-independent mechanism, and *KSR2* can inhibit this process. More importantly, co-IP results demonstrated that overexpression of *CRBN* in HEK293T cells significantly restored the K48-linked ubiquitination level of *AMPKα1* protein, which had been reduced by *KSR2* overexpression (Figure 7G). In summary, these findings indicate that *KSR2* inhibits *CRL4A^CRBN^* E3 ubiquitin ligase-mediated K48-linked ubiquitination and proteasomal degradation of *AMPKα1*, thereby stabilizing *AMPKα1* protein levels.

To identify the specific domain of *AMPKα1* involved in its interaction with *KSR2* and *CRBN*, we constructed three truncated *AMPKα1* plasmids based on domain positions: P1 (Δ1-294 aa), P2 (Δ295-396 aa), and P3 (Δ397-574 aa). Co-IP results showed that the interaction between *AMPKα1* and *KSR2* was abolished with the P1 construct, whereas the interactions with P2 and P3 constructs were comparable to that of the full-length plasmid (Figure 7H). These findings indicate that *KSR2* interacts with the 1-294 aa region of *AMPKα1*. To further predict the binding sites between *KSR2* and *AMPKα1*, molecular docking was performed using the HADDOCK web server. The results suggested that the 1-123 aa segment of *AMPKα1* forms multiple hydrogen bonds with *KSR2* (Figure 7I, Table S5). Based on these predictions, we constructed additional *AMPKα1* truncation plasmids: p4 (Δ1-123 aa), Co-IP analysis revealed that, similar to the P1 construct (Δ1-294 aa), the p4 (Δ1-123 aa) fragment exhibited significantly weakened interaction with *KSR2*, confirming that *KSR2* primarily interacts with the 1-123 aa segment of *AMPKα1* (Figure 7J). These findings confirm that *KSR2* predominantly interacts with the 1-123 aa region of *AMPKα1*.

Previous studies have demonstrated that *CRBN* interacts with both the 1-100 aa and 393-473 aa regions of *AMPKα1*. Furthermore, *CRBN* competitively binds to the 393-473 aa region of *AMPKα1* with the *AMPK*γ subunit, inhibiting the assembly of the *AMPK* complex and suppressing its activation 45. However, the role of *CRBN* binding to the 1-100 aa region of *AMPKα1* remains unclear. Based on our findings and previous research, we hypothesized that the *CRL4A^CRBN^* E3 ubiquitin ligase complex mediates K48-linked ubiquitin-proteasome degradation of *AMPKα1* via its N-terminal region. Co-IP results demonstrated that the *CRL4A^CRBN^* E3 ubiquitin ligase complex significantly increased K48-linked ubiquitination of full-length *AMPKα1*. However, this effect was abolished in the *AMPKα1* P4 construct (Δ1-123 aa) (Figure 7K), supporting our hypothesis. Collectively, these results suggest that both *KSR2* and *CRBN* interact with the N-terminal region of *AMPKα1*. This led us to investigate whether *KSR2* competitively binds *AMPKα1*, preventing *CRL4A^CRBN^*-mediated ubiquitin-proteasome degradation. To explore potential competitive interactions, we co-transfected Myc-*CRBN*, Flag-*AMPKα1*, and either *KSR2* (wild type, WT) or *KSR2* (372T mutant) plasmids into HEK293T cells. Co-IP results showed that overexpression of *KSR2* (WT) significantly reduced the interaction between *CRBN* and *AMPKα1*, while *KSR2* (372T mutant) overexpression had no noticeable effect (Figure 7L). Furthermore, Co-immunoprecipitation (Co-IP) results demonstrated that gradient overexpression of *KSR2* (wild type, WT) in HUVECs led to a progressive reduction in the interaction between endogenous *CRBN* and *AMPKα1* proteins (Figure 7M). We observed that while the components of the CRL4*^CRBN^* E3 ubiquitin ligase complex, including *CUL4A*, *DDB1*, and *CRBN*, are localized in both the nucleus and cytoplasm under physiological conditions, they predominantly reside in the nucleus 46-48. In contrast, *KSR2* and *AMPKα1* proteins are primarily localized in the cytoplasm 13, 49. To investigate the subcellular localization of *CUL4A*, *DDB1*, and *CRBN* under oxLDL stimulation, we analyzed their distribution in HUVECs. The results demonstrated no significant change in the total protein levels of *CUL4A*, *DDB1*, or *CRBN* upon oxLDL stimulation. However, a pronounced translocation from the nucleus to the cytoplasm was observed (Figure S15A). Immunofluorescence further confirmed a marked increase in *CRBN* cytoplasmic localization under oxLDL stimulation, accompanied by enhanced colocalization with *AMPKα1* in the cytoplasm (Figure S15B). These findings suggest that *KSR2* competes with *CRBN* in the cytoplasm for binding to the N-terminal region of *AMPKα1*, thereby inhibiting *CRL4A^CRBN^*-mediated K48-linked ubiquitin-proteasomal degradation and stabilizing *AMPKα1* protein levels.

Next, we identified the specific ubiquitination site of *AMPKα1*. Within the 1-123 aa region, four lysine residues (K40, K45, K52, K71) were identified, then we individually mutated the aforementioned lysine residues. Co-IP results showed that mutation of K52 abolished K48-linked ubiquitination mediated by the *CRL4A^CRBN^* E3 ubiquitin ligase complex, while mutations at other lysine residues had no significant effect (Figure 7N). These findings indicate that the *CRL4A^CRBN^* E3 ubiquitin ligase complex mediates K48-linked ubiquitin-proteasome degradation of *AMPKα1* via K52 in its N-terminal region. Intriguingly, molecular docking using the HADDOCK server predicted hydrogen bond interactions between *KSR2* and the K52 residue of *AMPKα1*. Taken together, these results demonstrate that *KSR2* inhibits *AMPKα1* K48-linked ubiquitin-proteasome degradation by competitively binding to *CRBN* at the K52 lysine residue, thereby stabilizing *AMPKα1* protein levels.

### Endothelial *CRBN* knockdown delays plaque progression in *Apoe*^⁻/⁻^ Mice by inhibiting endothelial inflammation and apoptosis

The above findings suggest that endothelial *CRBN* is a potential pro-atherogenic molecule, a role not previously characterized. Therefore, we validated this *in vivo*. Endothelial-specific *CRBN* knockdown was achieved via tail vein injection of AAV9-*ICAM2*-sh*CRBN*. Control mice received an equivalent dose of AAV9-*ICAM2*-shNC. Immunofluorescence staining confirmed that *CRBN* protein was specifically and efficiently knocked down in endothelial cells (mean fluorescence intensity, 60.03 ± 0.96 vs 10.79 ± 0.98, Cohen's D = 50.76, Figure S16). All groups were subsequently pair-fed a high-fat diet for 8 weeks. Compared to *Apoe*^-/-^ + shNC mice, *Apoe*^-/-^ + sh*CRBN* mice exhibited significantly smaller aortic plaques, with reduced macrophage content, whereas no significant differences were observed in smooth muscle cell content or collagen deposition within the plaques between the two groups (Figure S17A-F). Overall, these findings confirm that endothelial *CRBN* functions as a potential pro-atherogenic molecule.

Next, we investigated whether endothelial *CRBN* deficiency confers anti-inflammatory and anti-apoptotic effects in endothelial cells, similar to those observed with *KSR2* overexpression. Immunohistochemistry and aortic RT-qPCR results showed that, compared to *Apoe*^-/-^ + shNC mice, *Apoe*^-/-^ + sh*CRBN* mice exhibited significantly reduced expression of inflammatory markers in aortic plaques (Figure S17G-H). *En face* staining and Western blot results showed a significant reduction in endothelial apoptosis and increased *AMPKα1* protein levels in *Apoe*^-/-^ + AAV9-*ICAM2*-sh*CRBN* mice compared to *Apoe*^-/-^ + AAV9-*ICAM2*- shNC mice (Figure S17I-J). Taken together, these results indicate that endothelial-specific deletion of *CRBN* markedly alleviates endothelial inflammation and apoptosis, thereby attenuating the progression of atherosclerotic plaque.

### Endothelial *KSR2* attenuates atherosclerosis progression via *CRBN* in *Apoe*^-/-^ Mice

To determine whether endothelial *KSR2* restrains atherogenesis via *CRBN*, *Apoe*^-/-^ mice received tail-vein co-injection of AAV9-*ICAM2*-*KSR2* (AAV9-*KSR2*) together with AAV9-*ICAM2*-*CRBN* (AAV9-*CRBN*) or empty vector (AAV9-mock), followed by 8 weeks of high-fat diet. *En face* Oil Red O staining of aortas, together with Oil Red O and H&E staining of aortic root sections, revealed a significant increase in atherosclerotic lesion size in *Apoe*^-/-^ + AAV9-*KSR2* + AAV9-*CRBN* mice compared with *Apoe*^-/-^ + AAV9-*KSR2*. These results demonstrate that the atheroprotective effect of endothelial *KSR2* is mediated through *CRBN* (Figure S18A-B).

### Endothelial *KSR2* attenuates atherosclerosis progression by activating endothelial *AMPK* signaling

**Figure 8:**
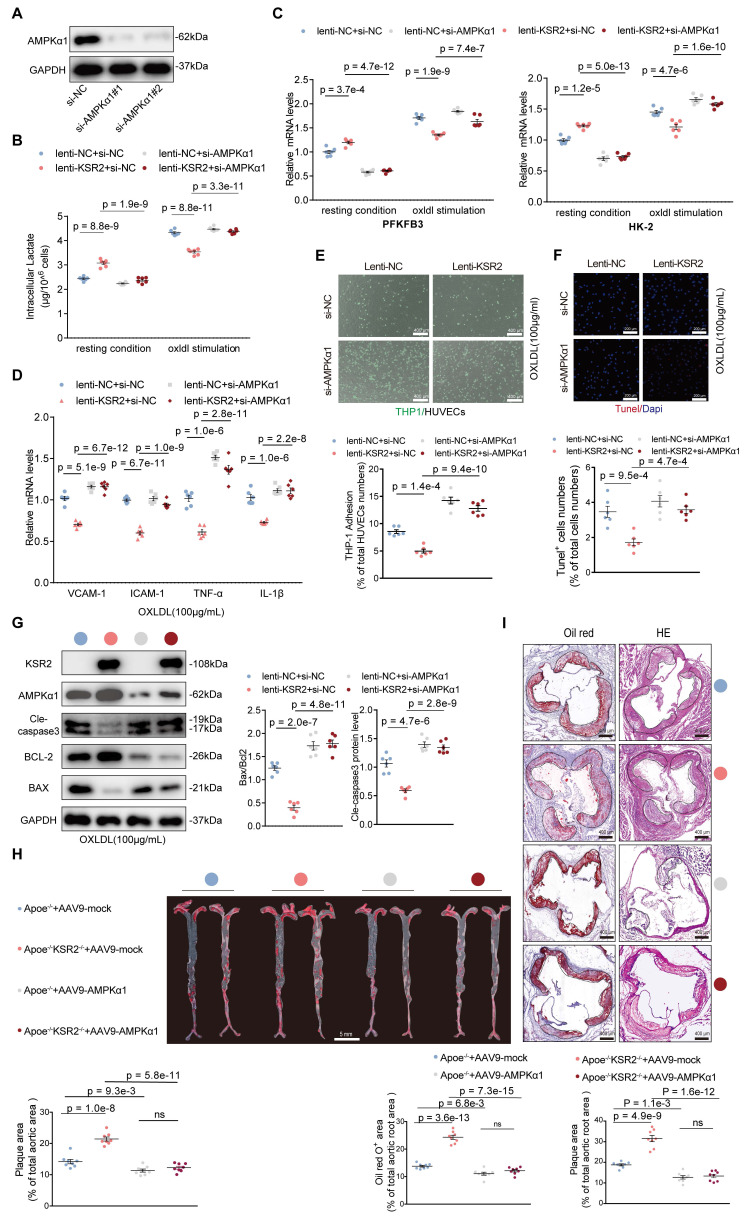
**Endothelial *KSR2* attenuates atherosclerosis progression through activation of the *AMPKα1* signaling pathway. A,** HUVECs were transfected with CTRL siRNA (si-NC), *AMPKα1* siRNA (si-*AMPKα1*, #1), or *AMPKα1* siRNA (si-*AMPKα1*, #2). Western blot (WB) analysis was performed to assess si-*AMPKα1* knockdown efficiency.** B** and** C**, Lentivirus-stably transfected lenti-*KSR2* HUVECs and lenti-NC HUVECs were transfected with si-NC or si-*AMPKα1* (#1 + 2) for 48 h, followed by treatment with or without oxLDL (100 μg/mL) for 24 h. **(B)** The Intracellular Lactate levels were measured. n = 6, ordinary one-way ANOVA. **(C)** WB analysis was performed to examine *PFKFB-3* and *HK-2* mRNA levels in HUVECs. n = 6, ordinary one-way ANOVA. **D-G,** Lentiviral stable transfection of HUVECs with lenti-NC or lenti-*KSR2* were transfected with si-NC or si-*AMPKα1* (#1 + 2) for 48 h, followed by 24 h of oxLDL (100 μg/mL) stimulation. **(D)** RT-qPCR was used to assess the mRNA levels of *VCAM-1*,* ICAM-1*, *TNF-α*, and *IL-1β* in HUVECs. n = 6, ordinary one-way ANOVA. **(E)** THP-1 adhesion assay assessing the adhesive capacity of HUVECs. Results are presented as the percentage of endothelial cells with adherent THP-1 cells. Scale bar = 400 μm. n = 6, ordinary one-way ANOVA. **(F)** Tunel staining was performed to detect apoptosis in HUVECs. Results are expressed as the percentage of Tunel-positive cells, with Tunel-positive cells in red and DAPI-stained nuclei in blue. Scale bars = 200 μm. n = 6, ordinary one-way ANOVA. **(G)** WB analysis was used to examine *Bax*, *Bcl-2*, and cleaved caspase-3 protein levels in HUVECs. n = 6, ordinary one-way ANOVA. **H,** Representative *en face* Oil Red O-stained aortas from *Apoe*⁻/⁻ + AAV9-mock, *Apoe*⁻/⁻*KSR2*⁻/⁻ + AAV9-mock, *Apoe*⁻/⁻ + AAV9-*AMPKα1*, *Apoe*⁻/⁻*KSR2*⁻/⁻ + AAV9-*AMPKα1* mice. Quantification of aortic lesion areas is shown. Scale bar = 5 mm. n = 8, ordinary one-way ANOVA.** I,** Oil Red O and H&E staining of aortic root cryosections from *Apoe*⁻/⁻ + AAV9-mock, *Apoe*⁻/⁻*KSR2*⁻/⁻ + AAV9-mock, *Apoe*⁻/⁻ + AAV9-*AMPKα1*, *Apoe*⁻/⁻*KSR2*⁻/⁻ + AAV9-*AMPKα1* mice. Quantification of aortic lesion areas is shown. Scale bar = 400 μm. n = 8, ordinary one-way ANOVA. ns, not significant.

Next, we investigated whether endothelial *KSR2* attenuates atherosclerosis via the *AMPK* signaling pathway both *in vivo* and *in vitro*. *In vitro* experiments, we used two independent siRNAs together to knock down *AMPKα1* expression in HUVECs (Figure 8A). Measurements of intracellular lactate, ATP/ADP levels (Figure 8B, Figure S19) and the mRNA levels of *PFKFB-3* and *HK-2* (Figure 8C) revealed that, under both basal and oxLDL-stimulated conditions, silencing *AMPKα1* expression significantly reversed the effects of *KSR2* overexpression on glycolysis in HUVECs. Similarly, RT-qPCR analysis demonstrated that the suppression of inflammatory factors, including *VCAM-1*,* ICAM-1*, *TNF-α*, and *IL-1β*, by *KSR2* overexpression was markedly diminished when *AMPKα1* was silenced (Figure 8D). Moreover, knockdown of *AMPKα1* significantly diminished the inhibitory effect of *KSR2* overexpression on THP-1 adhesion in HUVECs (Figure 8E). Furthermore, TUNEL assay (Figure 8F) and WB analysis (Figure 8G) showed that the anti-apoptotic effects of *KSR2* on HUVECs were significantly attenuated following *AMPKα1* knockdown.

In the *in vivo* experiments, endothelial *AMPK* signaling was selectively activated via tail vein injection of AAV9-*ICAM2*-constitutively active *AMPKα1* (AAV9-*AMPKα1*), while control mice received an equivalent dose of AAV9-*ICAM2*-mock (AAV9-mock). All groups were pair-fed a high-fat diet for 8 weeks. *En face* Oil Red O staining, Oil Red O and H&E staining of aortic root cryosections demonstrated that endothelial-specific activation of *AMPK* signaling significantly attenuated plaque progression in *Apoe*^-/-^*KSR2*^-/-^ mice compared with *Apoe*^-/-^ controls (Figure 8H-I). Collectively, these *in vivo* and *in vitro* findings indicate that *KSR2* mitigates endothelial inflammation and apoptosis by activating *AMPK* signaling, thereby suppressing the progression of atherosclerotic lesions.

### Rs12822146 risk allele impairs *AMPK* signaling in HUVECs

**Figure 9:**
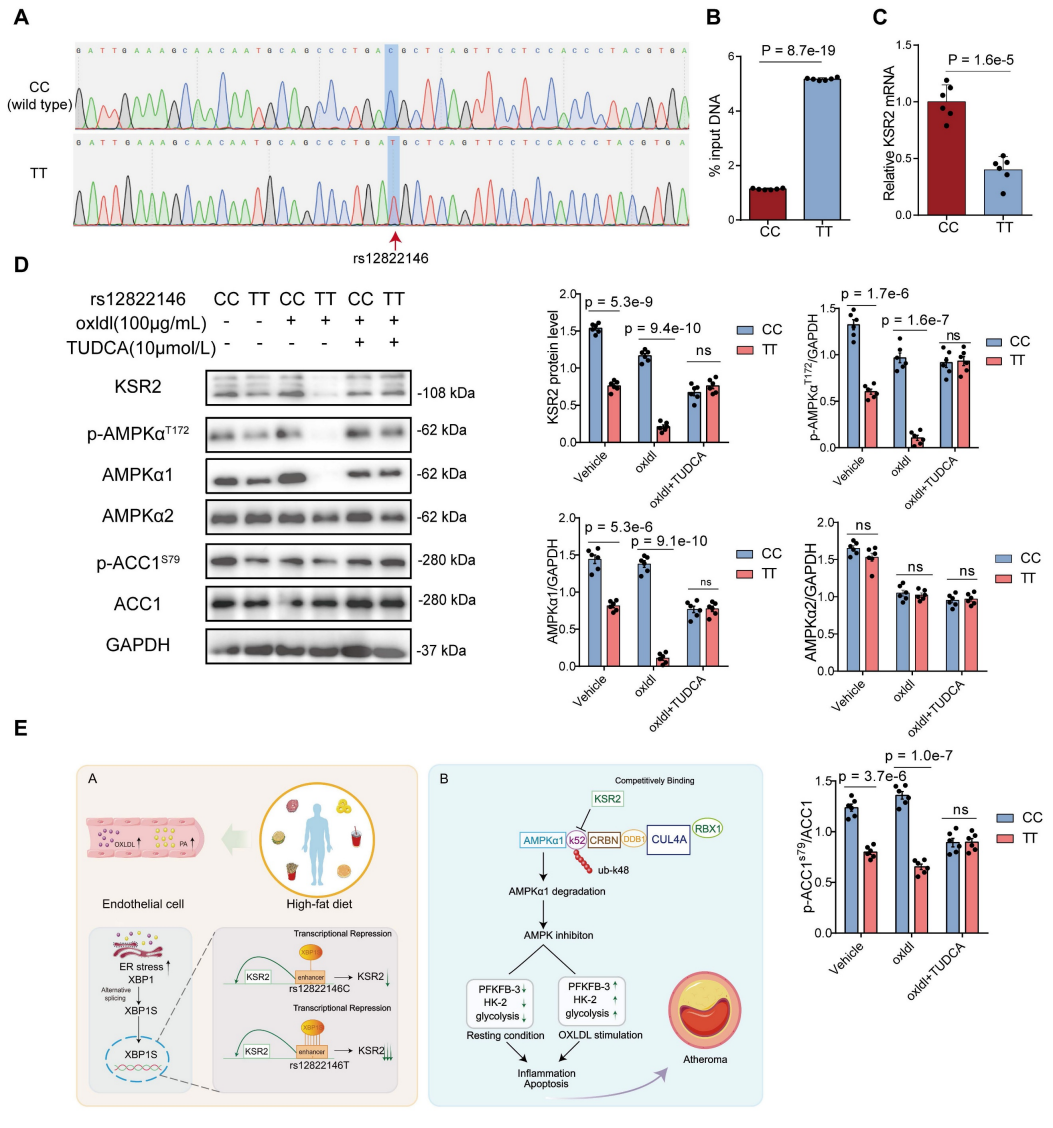
**Rs12822146 Risk Allele Impairs AMPK Signaling in HUVECs. A,** Cloning and sequencing results confirming HUVECs with different genotypes at the rs12822146 locus, with the rs12822146 site highlighted by a red arrow.** B,** ChIP-qPCR analysis showing greater enrichment of DNA fragments surrounding the rs12822146 locus with an *XBP1s* antibody in TT genotype HUVECs compared to CC genotype HUVECs. n = 6, 2-tailed unpaired Student's t-test. **C,**​ Real-time quantitative PCR (RT-qPCR) analysis showing differential expression of *KSR2* in CC versus TT genotype HUVECs. n = 6; two-tailed unpaired Student's t-test. **D,**​ Western blot revealing *KSR2*, p-*AMPK*α^T172^, *AMPKα1*,* AMPKα2*, p-*ACC1*^S79^ and *ACC1* levels in CC vs TT cells under basal conditions, after 24 h oxLDL (100 μg/mL) or after pretreatment with TUDCA (10 μmol/L) for 24 h prior to oxLDL (100 μg/mL) stimulation for an additional 24 h. n = 6, 2-tailed unpaired Student's t-test. **E,**​ Mechanistic illustration depicting how the rs12822146-*KSR2* axis influences the progression of atherosclerosis in endothelial cells. ns, not significant.

Finally, to directly validate the impact of different alleles of endothelial rs12822146 on *KSR2* expression and downstream *AMPK* signaling, we generated homozygous mutant HUVECs (genotype: TT) from wild-type cells (genotype: CC) via site-directed mutagenesis using CRISPR-Cas9 gene editing technology (Figure 9A). ChIP-qPCR analysis demonstrated significantly greater enrichment of DNA fragments surrounding the rs12822146 locus with an *XBP1s* antibody in TT genotype cells compared to CC controls (5.17 ± 0.05 vs 1.35 ± 0.03, Cohen's D = 92.65, Figure 9B). Consistent with this, RT-qPCR revealed a marked decrease in *KSR2* mRNA levels in TT HUVECs (1.00 ± 0.15 vs 0.4 ± 0.12, Cohen's D = 4.42, Figure 9C). We further examined the *KSR2*-*AMPK* pathway under oxLDL stimulation with or without the *XBP1s* inhibitor TUDCA. Western blot analysis showed that TT genotype cells exhibited reduced protein levels of *KSR2*, p-*AMPK*α^T172^, *AMPKα1*, and p-*ACC1^S79^*/*ACC1*, but not* AMPKα2*, compared to CC genotype cells. These suppressive effects were exacerbated by oxLDL stimulation and significantly attenuated by TUDCA pretreatment (Figure 9D). Collectively, these results demonstrate that the transcription factor *XBP1s* differentially binds the rs12822146 risk allele to repress *KSR2* expression and inhibit *AMPK* signaling in endothelial cells.

## Discussion

Although genome-wide association studies have revealed over 400 independent loci related to coronary artery disease in the past decade 4-8, the causal genes underlying these loci and their mechanistic roles in CAD pathogenesis remain elusive. In this study, we provide the first evidence that the rs12822146 allele polymorphism is associated with the expression of the *KSR2* gene in endothelial cells. Furthermore, *KSR2* activates the *AMPK* signaling pathway through a non-canonical mechanism in endothelial cells, thereby delaying the progression of atherosclerosis (Figure 9E).

It is estimated that 40%-60% of the susceptibility to coronary artery disease can be attributed to genetic factors 4, 50. Genome-wide association studies are considered a "golden key" for uncovering the associations between common genetic variations, primarily SNPs, and complex diseases. Majid Nikpay *et al.* 6 performed a meta-analysis integrating GWAS data from diverse populations and identified rs11830157 as a locus closely associated with CAD. In recessive models, the risk allele of rs11830157 was shown to increase the incidence of CAD by approximately 12%. However, due to the complexities of linkage disequilibrium and the intricate interactions between genetic and environmental factors, GWAS-identified tag SNPs may not always represent the true causal variants. Dual luciferase assays indicated that the tag SNP rs11830157 identified by GWAS is not a functional variant; rather, the linked SNP rs12822146 may represent the true disease-associated variant. We next explored the functional implications of this locus. Using dual luciferase assays, ChIP, electrophoretic mobility shift assays (EMSA and supershift EMSA), and CRISPR/Cas9 gene-editing techniques, we found that this locus resides within an enhancer region in endothelial cells. The risk allele of rs12822146 enhances the binding of the transcriptional repressor *XBP1s*, which suppresses the activity of the enhancer and ultimately downregulates *KSR2* gene expression in endothelial cells. *XBP1s*, the active isoform of *XBP1*, is generated under ER stress conditions through the *IRE1α* RNase-mediated splicing of *XBP1u* mRNA.

It then acts as a transcription factor, activating the unfolded protein response (UPR) to alleviate the accumulation of misfolded or unfolded proteins 24. However, the UPR functions as a double-edged sword, prolonged and excessive activation of the UPR can induce cellular damage via multiple mechanisms, including inflammation 51 and apoptosis 52. Previous studies have shown that excessive ER stress is implicated in various cardiovascular diseases, including atherosclerosis, and that inhibiting ER stress can improve cardiovascular function 53. Our findings suggest that *XBP1s*, as a repressive transcription factor, downregulates *KSR2* expression in endothelial cells. Functional studies of *KSR2* further confirmed its protective role against atherosclerosis in endothelial cells. These results provide new insights into the relationship between ER stress and atherosclerosis development, and more importantly, suggest that for patients carrying the T allele of rs12822146, dietary control and ER stress inhibition may have significant potential for preventing atherosclerosis. TUDCA, a conjugated bile acid, is widely used in clinical practice to promote bile acid excretion, has been reported to exert atheroprotective effects. For instance, Hamczyk *et al.* demonstrated that TUDCA treatment ameliorates the vascular phenotype in progeria mouse models and extends lifespan in *Apoe*^-/-^ *Lmna^LCS/LCS^ SM22α*-Cre mice 54. Similarly, Wang *et al.* found that TUDCA attenuates atherogenesis by suppressing the AIM2 inflammasome and enhancing macrophage cholesterol efflux capacity 55. Our results, together with these earlier findings, corroborate and extend the understanding of TUDCA's pleiotropic anti-atherosclerotic properties. Importantly, our study suggests a clinically relevant implication: TUDCA may offer potential benefit in primary prevention or secondary therapy of CVD—particularly in individuals carrying the risk allele (T) at the rs12822146 locus. However, additional clinical and basic research is needed to evaluate the safety and efficacy of this treatment strategy. It is also important to note that unique genetic backgrounds of CAD are observed across different ethnic groups 56. Therefore, further studies involving populations of African ancestry are required to validate the association between this locus and CAD in these groups. In summary, our study identifies the CAD-associated SNP rs12822146 as being linked to *KSR2* gene expression in endothelial cells, suggesting that endothelial *KSR2* may play a significant role in the pathogenesis and progression of atherosclerosis.

*KSR2* gene is located in the 12q24 chromosomal region, which has been implicated as a susceptibility locus for dyslipidemia and obesity 57, 58. However, it remains unclear whether *KSR2* can influence the development of atherosclerosis through a cell-autonomous mechanism. Using HFD ad libitum-fed *KSR2*^⁻/⁻^ mice, HFD pair-fed *Apoe*^⁻/⁻^*KSR2*^⁻/⁻^ mice and AAV9-*KSR2* endothelium-specific overexpression models, we demonstrated that endothelial *KSR2* regulates atherosclerosis progression independently of systemic lipid and glucose alterations. This is an intriguing finding because *KSR2* not only regulates systemic metabolic parameters, such as lipid levels, but changes in lipid levels can further modulate *KSR2* expression in endothelial cells, thereby influencing the development of atherosclerosis. These results suggest that the impact of *KSR2* on atherosclerosis is multifaceted, the development of *KSR2*-specific activators may offer a promising therapeutic approach for treating atherosclerosis-related diseases in clinical practice.

In this study, we elucidated the molecular mechanism by which *KSR2* attenuates atherosclerosis. Specifically, *KSR2* regulates endothelial glycolytic balance through activation of the *AMPK* signaling pathway, thereby inhibiting endothelial inflammation and apoptosis, which ultimately slows the progression of atherosclerosis. In response to vascular injury, endothelial cells become activated and produce proinflammatory cytokines and chemokines. These factors attract monocytes and neutrophils, which adhere to the activated endothelium, infiltrate the arterial wall, and initiate inflammation. The central role of inflammation in atherosclerosis was further established by the CANTOS trial (Canakinumab Anti-inflammatory Thrombosis Outcome Study), which demonstrated that targeted inhibition of *IL-1β* significantly reduced cardiovascular events 59-61. When endothelial injury becomes severe, endothelial cells undergo various forms of cell death, with apoptosis being a hallmark of vascular injury. This results in vascular leakage, inflammation, and coagulation. Additionally, numerous studies have shown that inhibiting endothelial cell apoptosis can alleviate the progression of atherosclerosis 62, 63. Both previous research and our RNA sequencing data suggest that *KSR2* is closely linked to cellular energy metabolism. Endothelial cells primarily rely on anaerobic glycolysis for energy production 27. However, the relationship between endothelial glycolysis and atherosclerosis is highly complex. A potential explanation for this relationship could be derived from the link between anaerobic glycolysis and endothelial cell proliferation. Endothelial proliferation predominantly depends on aerobic glycolysis for ATP generation, which provides the energy required for rapid cell division 64, 65. Endothelial cells allocate approximately 60% of ATP toward maintaining homeostasis, with the remaining 40% dedicated to proliferation 66. Furthermore, arterial regions susceptible to atherosclerotic plaque formation exhibit an elevated endothelial turnover rate. Providing adequate energy for endothelial repair and maintaining a certain level of proliferative activity is essential for protecting the arterial wall from atherosclerosis 65, 67. Moreover, moderate increases in glycolysis can protect endothelial cells by inhibiting their senescence, likely through an increase in the intracellular NAD pool 68, 69. On the other hand, excessive glycolytic activation leads to endothelial cell overproliferation, which contributes to increased permeability to proatherogenic lipoproteins and endothelial activation, accelerating atherosclerosis progression. The mechanisms underlying this process are yet to be fully elucidated. Previous studies have found that endothelial *HIF1A* promotes atherosclerosis by driving inflammation and proliferation in atheroprone regions through upregulation of glycolysis 29, 70. Given the variability in vascular sites, aging, and metabolic status, endothelial cell proliferation and glycolysis demands may differ, underscoring the importance of studying the relationship between endothelial glycolysis and atherosclerosis in various contexts. Overall, maintaining endothelial glycolysis within an optimal range appears beneficial.

Our study further reveals that *KSR2* stabilizes *AMPKα1* protein through a non-canonical ubiquitin-proteasome pathway, thereby activating endothelial *AMPK* signaling. Notably, our findings indicate that, in addition to promoting *AMPK*α phosphorylation at Thr172—as previously reported 18, 19, 34—*KSR2* also activates *AMPK* signaling by increasing *AMPKα1* protein abundance, a regulatory mechanism that has not been described in earlier studies. This prompted us to investigate the underlying molecular basis in greater detail. Through both *in vitro* and *in vivo* experiments, we demonstrated that K48-linked ubiquitin-proteasomal degradation of *AMPKα1* is a broadly occurring mechanism in atherosclerosis models. However, our *in vitro* data also suggest that under basal conditions, *AMPKα1* undergoes mild degradation that appears to be independent of the ubiquitin-proteasome system. This indicates that the regulation of *AMPKα1* protein stability may differ depending on physiological or pathological context. Previous studies have indicated that *AMPKα1* protein levels are reduced in the skeletal muscle of obese or diabetic mice, with the E3 ubiquitin ligase MG53 mediating the K48-linked ubiquitin-proteasomal degradation of *AMPK*α in diabetic models 35. In this study, we confirm that in an atherosclerosis model, endothelial *KSR2* competes with *CRBN* to bind the N-terminal of *AMPKα1*, particularly at the K52 site. This interaction inhibits the *CRL4A^CRBN^* E3 ubiquitin ligase complex from mediating K48-linked ubiquitin-proteasomal degradation of *AMPKα1*, thereby activating *AMPK* signaling. CRLs are the largest known family of E3 ubiquitin ligases 71, with approximately 20% of proteins degraded via the ubiquitin-proteasome system in human cells being catalyzed by CRLs 72. CRLs are multimeric complexes composed of Cullin family proteins, RING proteins, adaptor proteins, and substrate recognition subunits 45. Several previous studies have investigated the regulatory mechanisms of *CRL4A^CRBN^* on the *AMPK* signaling pathway, but their findings have been inconsistent. Seung-Joo Yang *et al.* 73 reported that *CRL4A^CRBN^* targets the *AMPK*γ subunit for proteasomal degradation via ubiquitination, with minimal effects on the stability of other subunits. In contrast, Eunju Kwon *et al.* 40 found that *CRL4A^CRBN^* mediates the degradation of the *AMPK*α subunit through K29- and K63-linked polyubiquitination. Moreover, Kwang Min Lee *et al.* 45 demonstrated that *CRBN* can directly bind to the α1 subunit, reducing its affinity for the γ1 subunit and thereby inhibiting *AMPK* phosphorylation and activation—an effect independent of the E3 ligase activity of the *CRL4A^CRBN^* complex. Collectively, these findings highlight the complexity of *CRBN*-mediated regulation of the *AMPK* pathway, which may vary across different cell types, tissues, stimuli, and pathological conditions. We speculate that some of these differences may be attributed to the subcellular localization of CRL4 E3 ligase complex components. Under physiological conditions, key components of the *CRL4A^CRBN^* complex—such as *CUL4A*, *DDB1*, and *CRBN*—are predominantly localized in the nucleus, suggesting that only limited amounts of active *CRL4A^CRBN^* complexes are present in the cytoplasm. Given that *AMPKα1* is primarily cytoplasmic, the regulatory effects of *CRBN* on *AMPK* signaling under basal conditions are likely mediated by its direct interaction with the C-terminal region of *AMPKα1*, which interferes with the binding of the γ1 subunit, consistent with the findings by Kwang Min Lee *et al.* 45 and independent of ubiquitin ligase activity. Our data further demonstrate that, in response to atherosclerosis-related stimuli, *CUL4A*, *DDB1*, and *CRBN* undergo marked cytoplasmic translocation in endothelial cells, leading to increased formation of cytoplasmic *CRL4A^CRBN^* complexes. Under these conditions, *CRBN* not only binds to the N-terminus of *AMPKα1* but also promotes its degradation through *CRL4A^CRBN^* mediated ubiquitination. It is important to note that regulation of *CRL4A^CRBN^* E3 ligase activity is highly complex, and how atherosclerosis-related stimuli mediate the nuclear-to-cytoplasmic translocation of *CUL4A*, *DDB1*, and *CRBN*, as well as whether additional mechanisms regulate the activity of the *CRL4A^CRBN^* complex, remains to be fully elucidated. Nevertheless, our findings consistently support that *CRBN* functions as a negative regulator of *AMPK* signaling. In contrast to the findings of Eunju Kwon *et al.* 40, we found that *CRL4A^CRBN^* mediates *AMPKα1* degradation primarily through K48-linked, rather than K63- or K29-linked, polyubiquitin chains. Although a single substrate can be modified by different ubiquitin linkages depending on the E3 ligase context 74, our ubiquitin linkage-specific assays in HEK293T cells showed that *KSR2* selectively suppresses K48-linked ubiquitination of exogenous *AMPKα1*. It is also recognized that the predominant form of ubiquitination may vary with cellular context or experimental conditions 75, which could contribute to the discrepancy between our results and those of Kwon *et al.* The activity of CRLs is tightly regulated by neddylation, a post-translational modification process involving the covalent attachment of the ubiquitin-like molecule *NEDD8* to a conserved lysine residue within the *Cullin* C-terminal domain 76. MLN4924, an inhibitor of the Neddylation pathway, has been shown to attenuate inflammation and prevent atherosclerosis 77, 78. Our findings further support the theoretical foundation for the potential anti-atherosclerotic effects of MLN4924, offering valuable insight for future therapeutic interventions targeting CRL-mediated pathways in atherosclerosis.

Thalidomide and its analogs are effective agents in the clinical treatment of multiple myeloma, with *CRBN* recognized as their primary molecular target. Acting as molecular glues, these compounds facilitate the recruitment of neo-substrates to *CRBN*, leading to their ubiquitin-proteasome-mediated degradation via the *CRL4A^CRBN^* complex 44. In our *in vitro* studies, we demonstrated that *CRL4A^CRBN^* promotes *AMPKα1* degradation in a lenalidomide-independent manner, as shown by *CRBN* knockdown, treatment with TD-165 (a PROTAC-based *CRBN* degrader), and lenalidomide exposure. *In vivo*, we used AAV9-sh*CRBN* to achieve endothelial-specific *CRBN* deletion and confirmed that *CRBN* functions as a pro-atherogenic factor. TD-165 effectively induced *CRBN* degradation *in vitro* 43. Previous studies have shown that in osteoarthritis models, *CRBN* exacerbates disease progression by inhibiting *AMPK* signaling in chondrocytes, and intra-articular injection of TD-165 mitigates cartilage destruction 79. Similarly, in heart failure models with reduced ejection fraction, *CRBN* impairs cardiac contractility by targeting Cav1.2α for *CRL4A^CRBN^* -mediated degradation, whereas TD-165 treatment improved cardiac function in both *in vitro* and *ex vivo* settings 80. Our findings provide the evidence that *CRBN* is a viable therapeutic target in atherosclerosis. Although TD-165 is effective at degrading *CRBN* in cells, its high plasma protein binding rate (> 99.9%) and large molecular weight limit its bioavailability and organ-targeting efficiency *in vivo* 43. Improving the pharmacokinetic profile of TD-165—such as enhancing bioavailability, reducing plasma protein binding, and increasing tissue-specific delivery—may facilitate its future therapeutic application in coronary artery disease.

Our study has several limitations. (1) Due to the difficulty in obtaining a sufficient number of coronary samples, we were unable to further validate the impact of the rs12822146 allele polymorphism on the expression of *KSR2* in human coronary endothelial cells through clinical studies. (2) We did not employ an endothelial-specific conditional *KSR2* knockout mouse model in this study, but we utilized multiple *in vivo* models—including HFD ad libitum-fed *KSR2*^⁻/⁻^ mice, pair-fed *Apoe*^⁻/⁻^*KSR2*^⁻/⁻^ mice, and *Apoe*^⁻/⁻^ mice with endothelial-specific *KSR2* overexpression via AAV9-*ICAM2* to exclude the confounding effects of metabolic abnormalities and to validate the protective role of endothelial *KSR2* in atherosclerosis. (3) Generation of a mouse model carrying the orthologous human risk allele could more directly elucidate the functional impact of the rs12822146-*XBP1s*-*KSR2* axis and offer stronger translational insights for coronary artery disease prevention in human carriers.

## Conclusions

In summary, we have revealed that the CAD-associated SNP rs12822146 allele regulates the expression of *KSR2* in endothelial cells by differentially binding to the transcription factor *XBP1s*. *KSR2* competitively binds to *AMPKα1* at the K52 site with *CRBN*, thereby inhibiting the K48-linked ubiquitin-proteasomal degradation of *AMPKα1* by the *CRL4A^CRBN^* E3 ligase complex. This leads to the activation of the *AMPK* signaling pathway, which subsequently suppresses endothelial inflammation and apoptosis by maintaining glycolytic balance. Our findings provide a comprehensive molecular explanation of the rs12822146-*KSR2*-atherosclerosis axis, with important implications for the primary prevention and secondary treatment of coronary heart disease.

## Supplementary Material

Supplementary methods, figures and tables.

## Abbreviations

ASCVD: atherosclerotic cardiovascular disease
CAD: coronary artery disease
GWAS: genome-wide association studies
SNP: single nucleotide polymorphism
*KSR2*: kinase suppressor of Ras 2
*KSR1*: kinase Suppressor of Ras 1
*AMPK*: AMP-activated protein kinase
Bp: base pair
Chip: chromatin immunoprecipitation
HUVEC: human umbilical vein endothelial cell
*H3K4me1*: mono-methyl-histone H3 lysine 4
*H3K27ac*: acetyl-histone H3 lysine 27
RT-qPCR: reverse transcription quantitative polymerase chain reaction
TUDCA: Tauroursodeoxycholic acid
WB: western blot
PF: pair feeding
TG: triglycerides
TC: total cholesterol
LDL-C: low-density lipoprotein cholesterol
HDL-C: high-density lipoprotein cholesterol
oxLDL: oxidized low-density lipoprotein
PA: palmitic acid
ECAR: extracellular acidification rate
CQ: chloroquine
CO-IP: co-immunoprecipitation
