# Supplementary material for: Endothelial KSR2 regulated by genetic variation protects against atherosclerosis through AMPKα1 stabilization
Source: Theranostics. 2026 Jan 1;16(5):2598–626. doi: 10.7150/thno.122864 (PMC12713192; doi:10.7150/thno.122864)

Supplemental Material

Reagents and antibodies

Antibody against *H3K4me1*(#5326), *H3K27ac* (# 8173), *cleaved-caspase3* (#9661), *Bax* (#2772), Phospho-*AMPKα*(Thr172) (#2535), Ubiquitin (#58395S), *DDB1* (#5428), *FLAG*-Tag (#14793), *MYC*-Tag (#2278), *HA*-Tag (#3724), Mouse *IgG1* (#5415) were from Cell Signal Technology (Boston, USA). Antibody against *Moma-2* (ab33451), *α-SMA* (ab124964), *AMPKα*1 (ab32047), *MKRN1* (ab72054), *HK-2* (ab209847), Goat Anti-Rabbit IgG H&L (Alexa Fluor® 594) (ab150080), Goat Anti-Mouse IgG H&L (Alexa Fluor® 594) (ab150116), Goat Anti-Mouse IgG H&L (Alexa Fluor® 488) (ab150113), Goat Anti-Rat IgG H&L (Alexa Fluor® 488) (ab150157) were from Abcam (Cambridge, UK). Antibody against *xbp1u/s* (24168-1-AP), *GAPDH* (10494-1-AP), *CD31*(28083-1-AP), *TNF-α*(60291-1-Ig), *IL-1β*(16806-1-AP), *AMPKα*2 (18167-1-AP), Phospho-*ACC1* (Ser79) (29119-1-AP), *ACC1*(21923-1-AP), *RMND5A* (17559-1-AP), *TRIM28* (15202-1-AP), *PEDF* (26045-1-AP), *CUL4A* (14851-1-AP), *CRBN* (28494-1-AP), HRP-conjugated Affinipure Goat Anti-Rabbit IgG(H+L) (SA00001-2), HRP-conjugated Affinipure Goat Anti-Mouse IgG(H+L) (SA00001-1) were from Proteintech Group, Inc. (Chicago, IL, USA). Antibody against *KSR2* (sc-100421) was from Santa Cruz Biotechnology. Antibody against *Bcl2* (A19693), *CD68* (A24386PM) was from ABclonal Biotechnology Co., Ltd. Antibody against *FBXo48* (orb183658) was from Biorbyt. Antibody against *RNF44* (HPA038981) was from AmyJet scientific (Wuhan, CHINA). TUDCA(HY-19696), Palmitic acid (HY-N0830), 2-DG(HY-13966), Cycloheximide (HY-12320), TD165(HY-130714), Lenalidomide (HY-A0003), Protein A/G Magnetic Beads (HY-K0202) were purchased from MedChemExpress (MCE, China). ox-LDL were purchased from Yiyuan

Biotechnologies (Guangzhou, China). MG-132(GC10383), 3-MA(GC68539), Chloroquine (GC19549) were purchased from GLPBIO (Montclair, CA, USA). Puromycin Solution was purchased from biosharp (Beijing, China). The atherosclerosis model diet was formulated based on the Clinton-Cybulsky diet (TP28251) sourced from Trophic Diets in Nantong, China, this composition includes 40% fat and 1.25% cholesterol. Total Cholesterol Assay kit (Cat A111-1), Triacylglycerol Assay kit (Cat A110-1), Glucose Assay kit (CAT A154-1-1) were from Nanjing Jiancheng Bioengineering Institute (Nanjing, China). Fluoroshield Mounting Medium with 4′ ,6-diamidino-2-phenylindole (DAPI, ab104139) from Abcam (Cambridge, UK). Lipofectamine 2000 (11668019), Lipofectamine RNAimax (13778100) were from ThermoFisher Scientific (Shanghai, China). One Step Mouse Genotyping Kit (PD101-01), ChamQ Universal SYBR qPCR Master Mix(Q711), HiScript III RT SuperMix for qPCR (+gDNA wiper) (R323-01) were from Vazyme (Nanjing, China). Hematoxylin-Eosin/HE Staining Kit (G1120), Masson's Trichrome Staining Kit(G1340), RIPA lysis buffer(R0010), Lactic acid (LA) content detection kit (BC2235) were from Solarbio (Beijing, China). Oil Red O (O1516) was from Sigma-Aldrich (St. Louis, MO). Trizol Reagent (Cat. No. 15596018) was from Invitrogen (Grand Island, NY). Protease inhibitor cocktail (CW2200), Phosphatase Inhibitor Cocktail (CW2383), SDS-PAGE Loading Buffer (CW0027) were from CWBIO (Beijing, China). Immobilon ECL ultra western HRP substrate (WBULS0500) was from Millipore (Bedford, MA, USA). Dual-Luciferase Reporter Assay kit (MA0518) was from MeilunBio (Dalian, China). SimpleChIP® Enzymatic Chromatin IP Kit （#9005） was from Cell Signal Technology（Boston, USA）. EMSA probe biotin-labeling kit (GS008), Chemiluminescent

EMSA kit (GS009), BCA protein assay kit (P0012), Nuclear and Cytoplasmic Protein Extraction Kit (P0028) was from Beyotime (Shanghai, China). In Situ Cell Death Detection Kit, TMR Red (Cat. No. 12156792910) was from Roche (Indiana, IN, USA). ATP/ADP ratio chemiluminescence assay kit (E-BC-F004) was from Elabscience (Wuhan, CHINA).

Table S1

Characteristics of patients diagnosed without and with CAD. Data are presented as mean ± SEM, n = 5 in each group. Statistical significance was determined using a two-tailed unpaired Student's t-test, with $p < 0.05$ considered significant. Abbreviations: BW, body weight; TC, total cholesterol; TG, triglycerides; HDL, high-density lipoprotein; LDL, low-density lipoprotein; BG, blood glucose.

	**Diagnosed without CAD**	**Diagnosed with CAD**	**P value**
**Age (year)**	55.6±5.14	52.4±4.46	0.9444
**BW (Kg)**	74.8±3.94	72.4±5.56	0.9683
**Systolic pressure**<br>**Diastolic pressure**<br>**(mmHg)**	108.8±7.91<br>60.2±3.7	108.4±10.58<br>61.2±7.4	0.9365<br>0.8016
**TC (mmol/l)**	3.90±0.09	3.78±0.13	0.6667
**TG (mmol/l)**	1.62±0.05	1.71±0.07	0.4127
**HDL (mmol/l)**	1.18±0.04	1.07±0.03	0.0476

**LDL (mmol/l)**	2.17±0.07	2.52±0.04	0.0079
**BG (mmol/l)**	5.84±0.23	5.86±0.2	0.9524

Table S2

The primer sequences used for the target genes.

Genotyping the mice

**Sequence**	**Gene Name**
TGAGTGCGGACTGTTGAT	*KSR2*-F
CTTCTGGACGACTGAGGG	*KSR2*-R
TGCCTAGTCTCGGCTCTGAACTAC	APOE-F
CAACCTGGGCTACACACTAATTGAG	APOE-R

CHIP

**Sequence**	
AGCAAGGAGCCTAGGATTGAAA	*KSR2*(Forward)
CCGGATACCAGGATGCTCTCA	*KSR2*(Reverse)

EMSA

**Sequence**	
GCAGCCCTGACGCTCAGTTCC	Ref-seq（Forward)
GGAACTGAGCGTCAGGGCTGC	Ref-seq（Reverse)
GCAGCCCTGATGCTCAGTTCC	Alt-seq（Forward)
GGAACTGAGCATCAGGGCTGC	Alt-seq（Reverse)

GCTACGTTAGCCTAGTGACT	Non-specific（Forward)
AGTCACTAGGCTAACGTAGC	Non-specific（Reverse)

Crispr-cas9

Sequence	
AGCAAGGAGCCTAGGATTGAAA	P1-F (human)
CCGGATACCAGGATGCTCTCA	P2-R (human)
TGCATCTTGTGCACCGATTTC	P3-F (human)
TCTTGGACACCATACGACCAG	P4-R (human)

RT-qPCR

Sequence	Gene Name
GAGGAGACCCAGCAAGGTTAG	*KSR2*-F (mouse)
CTGGAGTCTGGCTGGTAAGG	*KSR2*-R (mouse)
CAGGCATGAAACCCAACCTC	*KSR2*-F (human)
GGTTTCGCTTTGGCAGTTTC	*KSR2*-R (human)
GTTCCAGCGAGGGTCTACC	VCAM-1-F (mouse)
ACTCTTGGCAAACATTAGGTGT	VCAM-1-R (mouse)
TGCAAGTCTACATATCACCCAAGAA	VCAM-1-F (human)
GTAGACCCTCGCTGGAACAG	VCAM-1-R (human)
TGCCTCTGAAGCTCGGATATAC	ICAM-1-F (mouse)
TCTGTCGAACTCCTCAGTCAC	ICAM-1-R (mouse)
CCCACAGTCACCTATGGCAA	ICAM-1-F (human)
GAGACCTCTGGCTTCGTCAG	ICAM-1-R (human)

GCAACTGTTCCTGAACTCAACT	IL-1β-F (mouse)
ATCTTTTGGGGTCCGTCAACT	IL-1β-R (mouse)
GCCCTAAACAGATGAAGTGCT	IL-1β-F (human)
GAACCAGCATCTTCCTCAG	IL-1β- R (human)
CACGCTCTTCTGTCTACTG	TNFα-F (mouse)
AAGATGATCTGAGTCTGAGG	TNFα-R (mouse)
TGGCCCAGGCAGTCAGA	TNFα-F (human)
GGTTTGCTACAACATGGGCTACA	TNFα-R (human)
ATGGAATTAGAGCGCCAAGA	PFKFB3-F (mouse)
CATTTCAGGTATGGCATCTCC	PFKFB3-R (mouse)
CTCGCATCAACAGCTTTGAGG	PFKFB3-F (human)
TCAGTGTTTCCTGGAGGAGTC	PFKFB3-R (human)
CAACTCCGGATGGGACAG	HK2-F (mouse)
CACACGGAAGTTGGTTCCTC	HK2-R (mouse)
GAGCCACCACTCACCCTACT	HK2-F (human)
CCAGGCATTCGGCAATGTG	HK2-R (human)
TGTCTCTGGAGGAGAGCTATTTGA	*AMPKα*1-F (mouse)
GGTGAGCCACAGCTTGTTCTT	*AMPKα*1-R (mouse)
GGAGCCTTGATGTGGTAGGAA	*AMPKα*1-F (human)
TCAAATAGCTCTCCTCCTGAGAC	*AMPKα*1-R (human)
CAGAAGATTCGCAGTTTAGATGTTGT	*AMPKα*2-F (mouse)
ACCTCCAGACACATATTCCATTACC	*AMPKα*2-R (mouse)

TCCTCAACACCTCAGCGTTC	*AMPKα*2-F (human)
CTTCCGGTCAAAGAGCCAGT	*AMPKα*2-R (human)
GGCTGTATTCCCCTCCATCG	β-actin- F (mouse)
CCAGTTGGTAACAATGCCATGT	β-actin- R (mouse)
CATGTACGTTGCTATCCAGGC	β-actin- F (human)
CTCCTTAATGTCACGCACGAT	β-actin- R (human)

Table S3

Sequences of gene-shRNA.

Gene Name	Sequence
Xbp1s-siRNA (human)	UAGAAAAUCAGCUUUUACGAGAGAA
KSR2- siRNA#1 (human)	CCCUCUAGUCAUCAGAAUAAGAGCA
KSR2- siRNA#2 (human)	GGGUUACUCAAACAAACAAUCCAAA
KSR2- siRNA#3 (human)	CCTCAGGAATGTCCACATGTC
CUL4A-siRNA (human)	AAGAAGAUUAACACGUGCUGG
CRBN-siRNA (human)	AAGTGCCAGATATTTCCTTCA
MKRN1- siRNA (human)	CACAGGCG AAGCTGAGTCAAG
RMND5A - siRNA (human)	CACCAUAUGUUCACCUACU
TRIM28- siRNA (human)	GCAUGAACCCCUUGUGCUG
PEDF- siRNA (human)	GGAAAUUCCCGAUGAGAUCUUTT
FBXo48- siRNA (human)	Santa cruz (#sc-94411)
RNF44- siRNA (human)	CGUUCAUGGUUGAUCUCCACG
*AMPKα*1- siRNA#1(human)	CAAAGUCGACCAAAUGAUA

*AMPKα*1- siRNA#2 (human)	GAAGGUUGGCAAACAUGAATT

Table S4

SNPs in High Linkage Disequilibrium with rs11830157 ($r^2 \geq 0.8$) in CHB, GIH, or CEU Populations. Abbreviations: Ref, reference allele; Alt, alternative allele; MAF, minor allele frequency; CHB, HAn Chinese in Beijing, China; GIH, Gujarati Indian from Houston, Texas; CEU, Utah Residents (CEPH) with Northern and Western European Ancestry

	Allele		LD Coefficient with rs11830157 (r^2)				
**snp**	**Ref>Alt**	**MAF**	**CHB**	**GIH**	**CEU**	**Location**	**Distance (bp)**
**rs12814988**	T>G	0.25	0.69242	1	0.603445	12:117827344	292
**rs874560**	T>C	0.28	0.69242	1	0.711249	12:117830660	3024
**rs7136032**	C>T	0.28	0.69242	1	0.711249	12:117831465	3829
**rs12825364**	A>G	0.28	0.69242	1	0.687151	12:117832826	5190
**rs1155759**	G>A	0.24	0.69242	0.978	0.628376	12:117835671	8035
**rs1074482**	C>T	0.26	0.662649	0.935	0.30759	12:117845711	18075
**rs71099081**	T_{11}>T_{10}/T_{12}	0.003/0.094	0.558157	0.914	0.54046	12:117840106-117840116	12470
**rs12822146**	C>T	0.16	0.662649	0.893	0.37237	12:117837586	9950

Table S5

Polar interactions between *KSR2* and *AMPKα*1. PDBePISA was used to analyze the HADDOCK-predicted binding model. Note that the *AMPKα*1 protein structure (6C9H) is incomplete; therefore, the predicted *AMPKα*1 sites in this model correspond to actual sites (Q13131) with an additional 9 residues.

	Residues in *KSR2*	Distance (Å)	Residues in *AMPKα*1
Hydrogen bonds			
1	CYS 348(N)	3.80	ASP 22(OD2)
2	GLN 354(N)	3.85	ASP 105(OD1)

3	ARG 366(HE)	1.69	ASP 114(OD2)
4	HIS 372(NE2)	3.63	GLN 319(O)
5	HIS 342(O)	3.09	GLY 11(N)
6	SER 343(O)	2.97	GLY 11(N)
7	SER 347(O)	2.74	ASP 22(N)
8	GLU 349(OE1)	1.84	LYS 43(HZ1)
9	PRO 374(O)	1.89	ARG 74(HH21)
10	THR 379(OG1)	2.08	GLN 83(HE21)
11	GLU 385(OE1)	3.39	SER 89(N)
Salt bridges			
1	ARG 366(NE)	2.69	ASP 114(OD2)
2	ARG 366(NH2)	3.50	ASP 114(OD2)
3	GLU 349(OE1)	2.67	LYS 43(NZ)
4	GLU 349(OE2)	3.06	LYS 43(NZ)

Figure S1

Coronary artery disease (CAD) associated SNP rs12822146 allelic variation correlates with *KSR2* Expression in endothelial cells. A, The region encompassing all common SNPs with $r^2 \geq 0.8$ with respect to the SNP rs11830157 in CHB（Han Chinese in Beijing, China）, GIH(Gujarati Indian from Houston, Texas), CEU(Utah Residents (CEPH) with Northern and Western European Ancestry) population. The DNaseI, CTCF, *H3K4me1*, and *H3K27ac* signals surrounding this region were visualized using publicly available ENCODE datasets on the UCSC Genome Browser [Expanded Encyclopedias of DNA Elements in the Human and Mouse Genomes]. Data from aortic, thoracic aortic, and coronary artery tissues from different patients were used for analysis. Blue dashed boxes indicate regions of open chromatin， the red dashed line marks the position of SNP rs12822146. **B** and **C,** ChIP-qPCR showing no enhancer activity in the region surrounding rs12822146 in **（B）** smooth muscle cells and **(C)** macrophages. Pulldown of *H3K4me1* (mono-methyl-histone H3 lysine 4), *H3K27ac* (acetyl-histone H3 lysine 27) was performed to assess the enrichment of chromatin fragments containing rs12822146. n = 5, 2-tailed unpaired Mann-Whitney U test due to small simple sizes. **D** and **E,** ChIP-qPCR showing no specific binding of **（D）** MEIS1 or **(E)** GATA2 to the region surrounding rs12822146 in endothelial cells, with or without oxLDL stimulation (100 μg/mL). n = 6, 2-tailed unpaired Student's t-test. **F** and **G,** ChIP-qPCR showing no specific binding of XBP1s to the region surrounding rs12822146 in **（F）** smooth muscle cells and **(G)** macrophages, with or without oxLDL stimulation (100 μg/mL). n = 5, 2-tailed unpaired Mann–Whitney U test due to small sample size. **H,** Competitive EMSA demonstrating allele-specific competitive binding of nuclear proteins from endothelial cells to the rs12822146 locus. **I** and **J,** HUVECs were transfected with TUDCA (10 μmol/L, 24h) or DMSO for 48 h, followed by treatment with or without oxLDL (100 μg/mL) for 24 h. **(I)** RT-qPCR and **（J）** western blot showing changes in *KSR2* expression. n = 6. 2-tailed unpaired Student's t-test, except for the left panel in **J** employed 2-tailed unpaired Mann-Whitney U test due to non-normal distribution. ns, not significant.

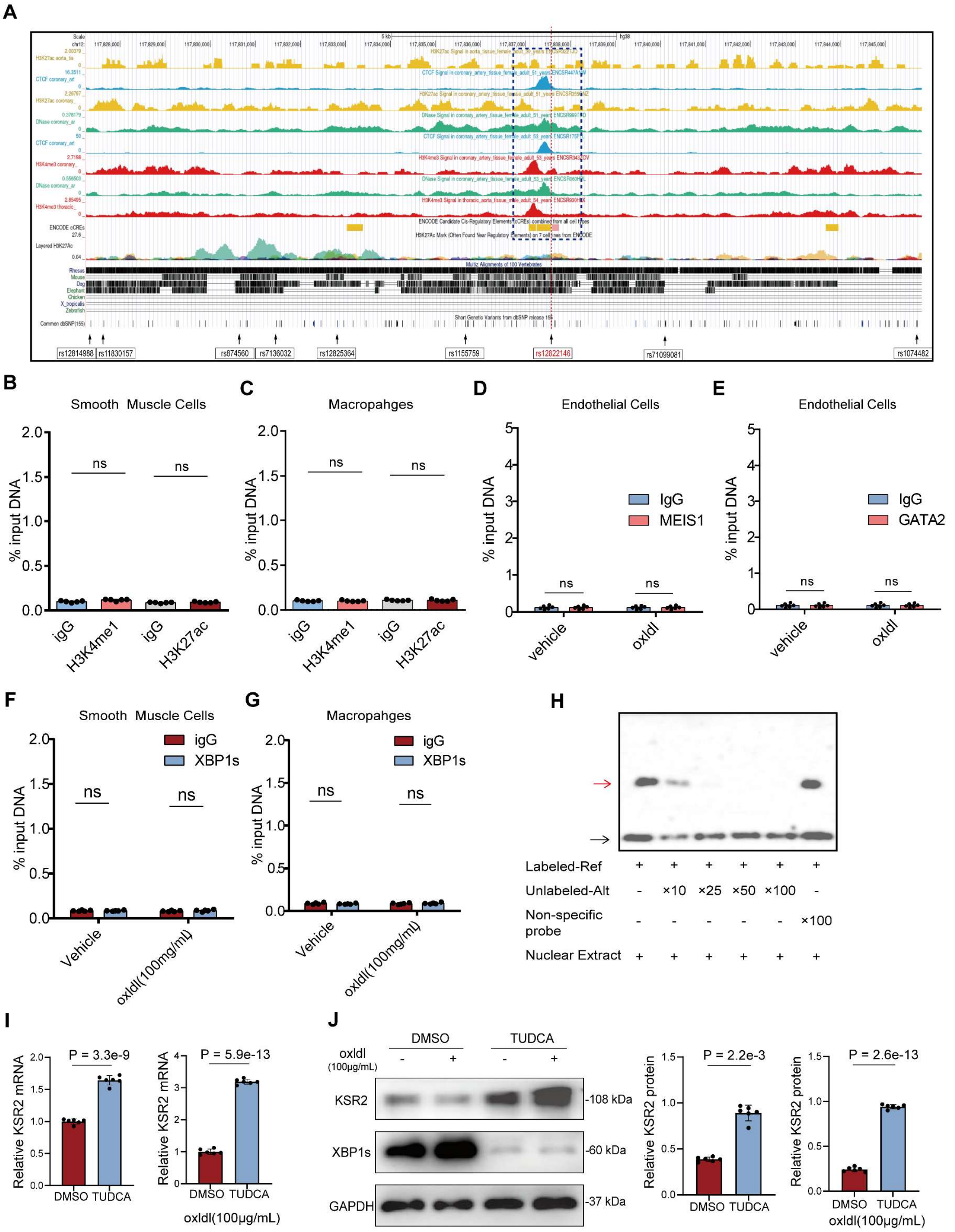
A
rs12814988
rs11830157
rs874560
rs7136032
rs12825364
rs1155759
rs12822146
rs71099081
rs1074482
B
Smooth Muscle Cells
% input DNA
ns
igG
H3K4me1
H3K27ac
C
Macropahges
D
Endothelial Cells
IgG
MEIS1
vehicle
oxldl
E
Endothelial Cells
IgG
GATA2
F
Smooth Muscle Cells
igG
XBP1s
Vehicle
oxldl(100mg/ml)
G
Macropahges
H
Labeled-Ref
Unlabeled-Alt
×10
×25
×50
×100
Non-specific probe
Nuclear Extract
I
Relative KSR2 mRNA
P = 3.3e-9
P = 5.9e-13
DMSO
TUDCA
oxldl(100μg/mL)
J
DMSO
TUDCA
oxldl (100μg/mL)
KSR2
XBP1s
GAPDH
-108 kDa
-60 kDa
-37 kDa
Relative KSR2 protein
P = 2.2e-3
P = 2.6e-13

Figure S2

Localization and dynamics of *KSR2* expression within atherosclerotic plaques. A and B, Immunofluorescence staining of cryosections from intermediate and advanced coronary lesions showing *KSR2* localization in **(A)** smooth muscle cells and **(B)** macrophages. *KSR2* is shown in red, *α-SMA* or *CD68* in green, and nuclei (DAPI) in blue. Scale bars = 100 μm. EC, endothelial cells; N, neointima; M, media; A, adventitia. White dashed lines indicate the endothelial boundary within the plaque. **C,** Immunofluorescence staining of mouse aortic cryosections showing *KSR2* localization within atherosclerotic plaques. *KSR2* is shown in red; *CD31*, *α-SMA*, or *CD68* in green; and nuclei (DAPI) in blue. Scale bars = 100 μm. L, lumen. **D-F,** Western blot analysis of *KSR2* expression in **(D)** primary aortic endothelial cells, **(E)** aortic smooth muscle cells, and **(F)** peritoneal macrophages isolated from mice fed a high-fat diet for 0, 4, 8, and 12 weeks. n = 5, Kruskal-Wallis test with Dunn's multiple comparisons test due to small sample sizes. ns, not significant.

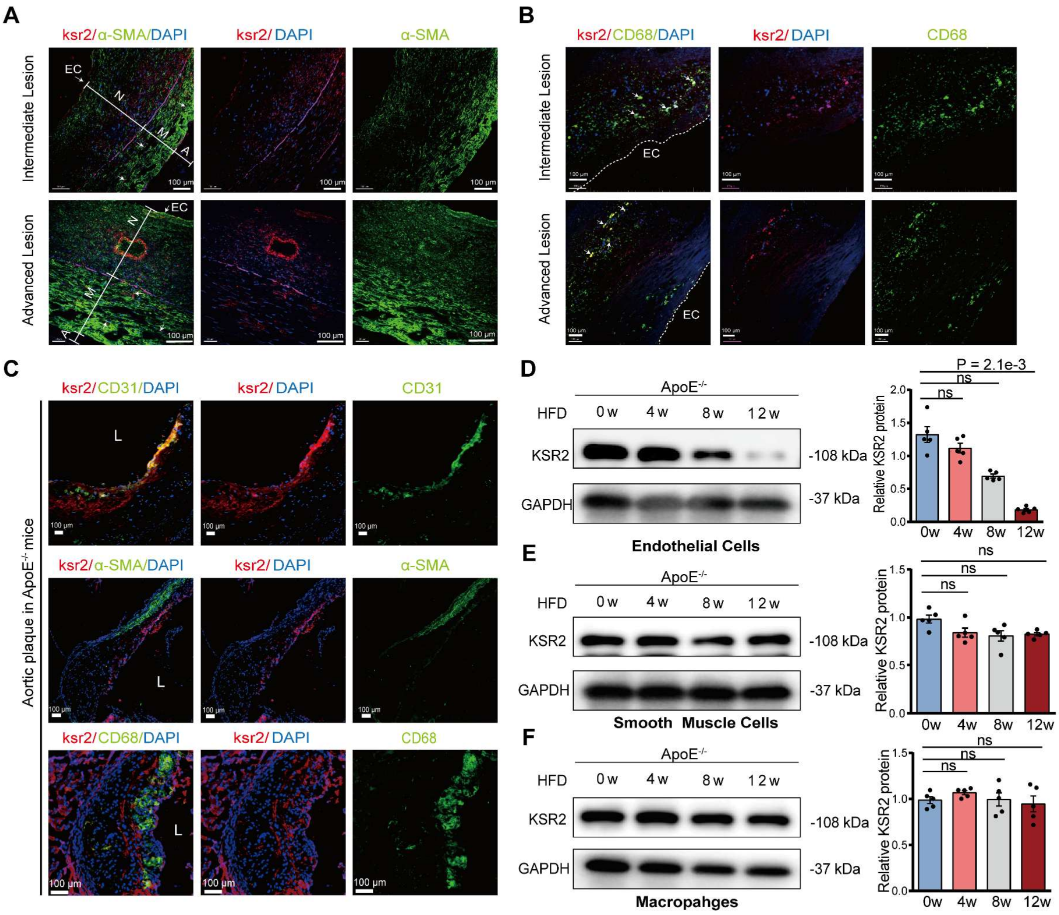

Figure S3

General Characteristics of wild-type (WT) and *KSR2*$^{-/-}$ mice. Eight-week-old WT and *KSR2*$^{-/-}$ mice were fed a high-fat diet (HFD) ad libitum for 12 weeks. **A,** Schematic diagram of *KSR2* knockout mice. **B and C,** Western blot **(B)** and RT-qPCR **(C)** analyses of aortic tissue were performed to verify *KSR2* knockout efficiency. n = 8, 2-tailed unpaired Mann–Whitney U test due to non-normal distribution. **D-K,** Measurements of **(D)** body weight, **(E)** serum triglycerides, **(F)** low-density lipoprotein cholesterol (LDL-C), **(G)** high-density lipoprotein cholesterol (HDL-C), **(H)** total cholesterol, **(I)** blood glucose levels, **(J)** serum insulin levels and **(K)** HOMR-IR index in WT and *KSR2*$^{-/-}$ mice. n = 6. 2-tailed unpaired Student's t-test, except for serum triglycerides **(D)**, which were analyzed using the 2-tailed unpaired Mann–Whitney U test due to non-normal distribution. HOMA-IR = fasting insulin (μIU/mL) × fasting glucose (mmol/L) / 22.5. ns, not significant.

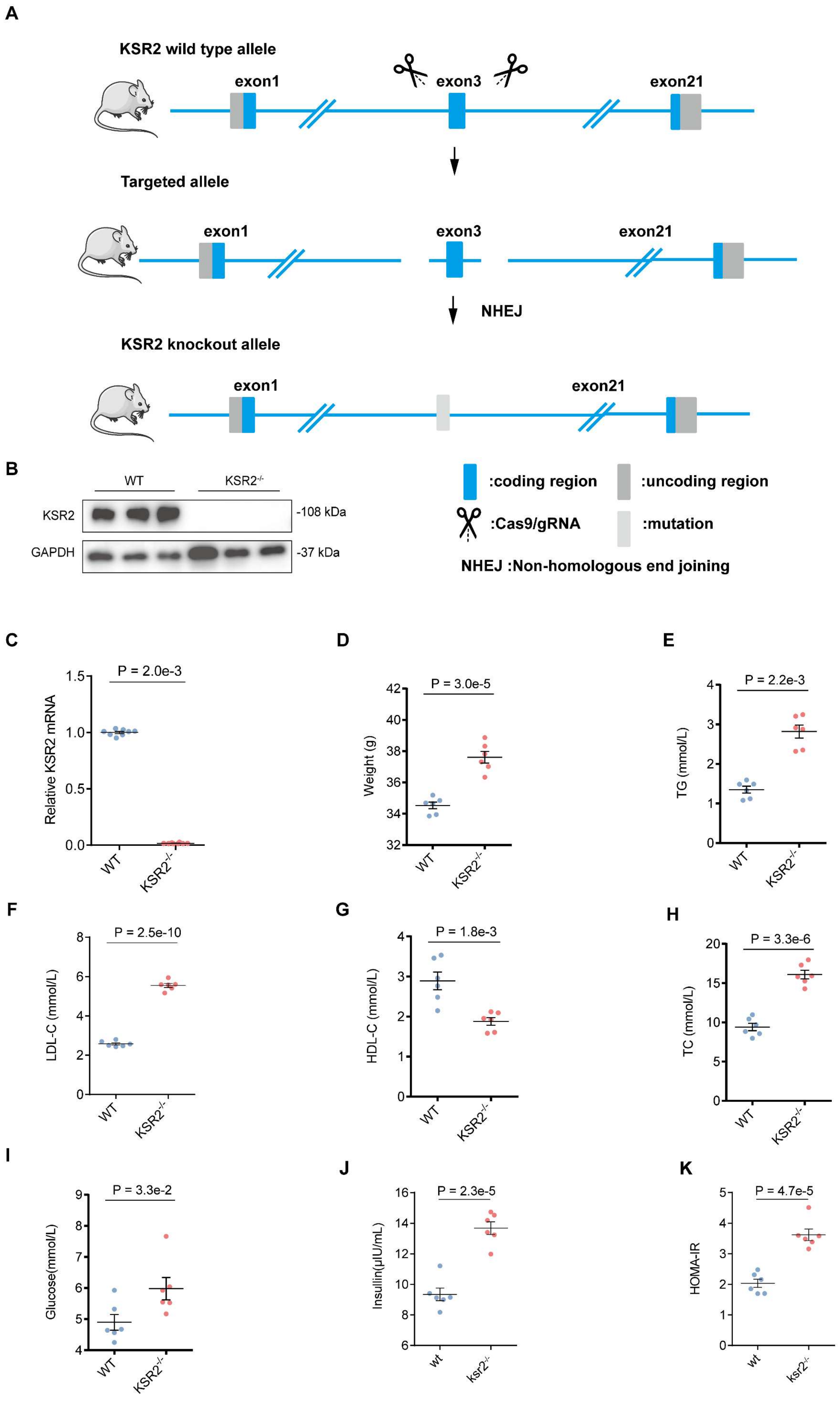
A
KSR2 wild type allele
exon1
exon3
exon21
Targeted allele
exon1
exon3
exon21
NHEJ
KSR2 knockout allele
exon1
exon21
B
WT
KSR2-/-
KSR2
-108 kDa
GAPDH
-37 kDa
:coding region
:uncoding region
:Cas9/gRNA
:mutation
NHEJ :Non-homologous end joining
C
P = 2.0e-3
Relative KSR2 mRNA
WT
KSR2-/-
D
P = 3.0e-5
Weight (g)
WT
KSR2-/-
E
P = 2.2e-3
TG (mmol/L)
WT
KSR2-/-
F
P = 2.5e-10
LDL-C (mmol/L)
WT
KSR2-/-
G
P = 1.8e-3
HDL-C (mmol/L)
WT
KSR2-/-
H
P = 3.3e-6
TC (mmol/L)
WT
KSR2-/-
I
P = 3.3e-2
Glucose(mmol/L)
WT
KSR2-/-
J
P = 2.3e-5
Insulin(μIU/mL)
wt
ksr2-/-
K
P = 4.7e-5
HOMA-IR
wt
ksr2-/-

Figure S4

General characteristics of Apoe$^{-/-}$ and Apoe$^{-/-}$*KSR2*$^{-/-}$ mice. Eight-week-old Apoe$^{-/-}$ and Apoe$^{-/-}$*KSR2*$^{-/-}$ mice were pair-fed a high-fat diet (HFD) for 12 weeks. Measurements of **(A)** body weight, **(B)** serum triglycerides, **(C)** low-density lipoprotein cholesterol (LDL-C), **(D)** high-density lipoprotein cholesterol (HDL-C), **(E)** total cholesterol, **(F)** blood glucose levels, **(G)** serum insulin levels and **(H)** HOMR-IR index in Apoe$^{-/-}$ and Apoe$^{-/-}$*KSR2*$^{-/-}$ mice. n = 10. 2-tailed unpaired Student's t-test, except for serum triglycerides **(B)**, which were analyzed using the 2-tailed unpaired Mann–Whitney U test due to non-normal distribution. HOMA-IR = fasting insulin (μIU/mL) × fasting glucose (mmol/L) / 22.5. ns, not significant.

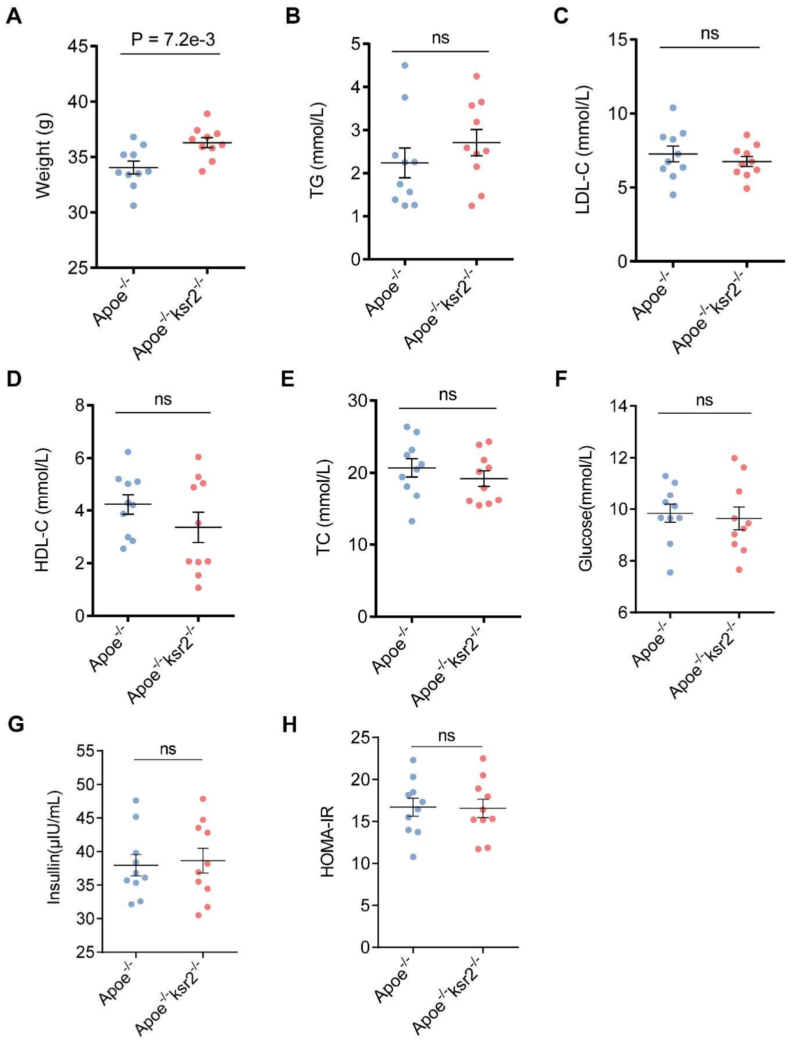

Figure S5

Metabolic profile of Apoe$^{-/-}$ mice with endothelial-specific overexpression of *KSR2*. Eight-week-old Apoe$^{-/-}$ mice received tail vein injections of equal doses of AAV9-ICAM2-*KSR2* (AAV9-*KSR2*) or AAV9-ICAM2-mock (AAV9-mock), followed by 12 weeks of high-fat diet feeding. **A,** Ex vivo imaging was used to detect the successful, vascular-specific distribution of AAV9-*KSR2* in Apoe$^{-/-}$ mice following tail vein injection. Scale bar = 5 mm. **B,** RT-qPCR analysis of *KSR2* mRNA levels in the aortic tissue of Apoe$^{-/-}$ + AAV9-mock and Apoe$^{-/-}$ + AAV9-*KSR2* mice. n = 5, 2-tailed unpaired Mann-Whitney U test due to small sample sizes. **C-H,** Measurements of **(C)** body weight, **(D)** serum triglycerides, **(E)** low-density lipoprotein cholesterol (LDL-C), **(F)** high-density lipoprotein cholesterol (HDL-C), **(G)** total cholesterol, **(H)** blood glucose levels, **(I)** serum insulin levels and **(J)** HOMR-IR index in Apoe$^{-/-}$ + AAV9-mock and Apoe$^{-/-}$ + AAV9-*KSR2* mice. n = 8, 2-tailed unpaired Student's t-test. HOMA-IR = fasting insulin (μIU/mL) × fasting glucose (mmol/L) / 22.5. ns, not significant.

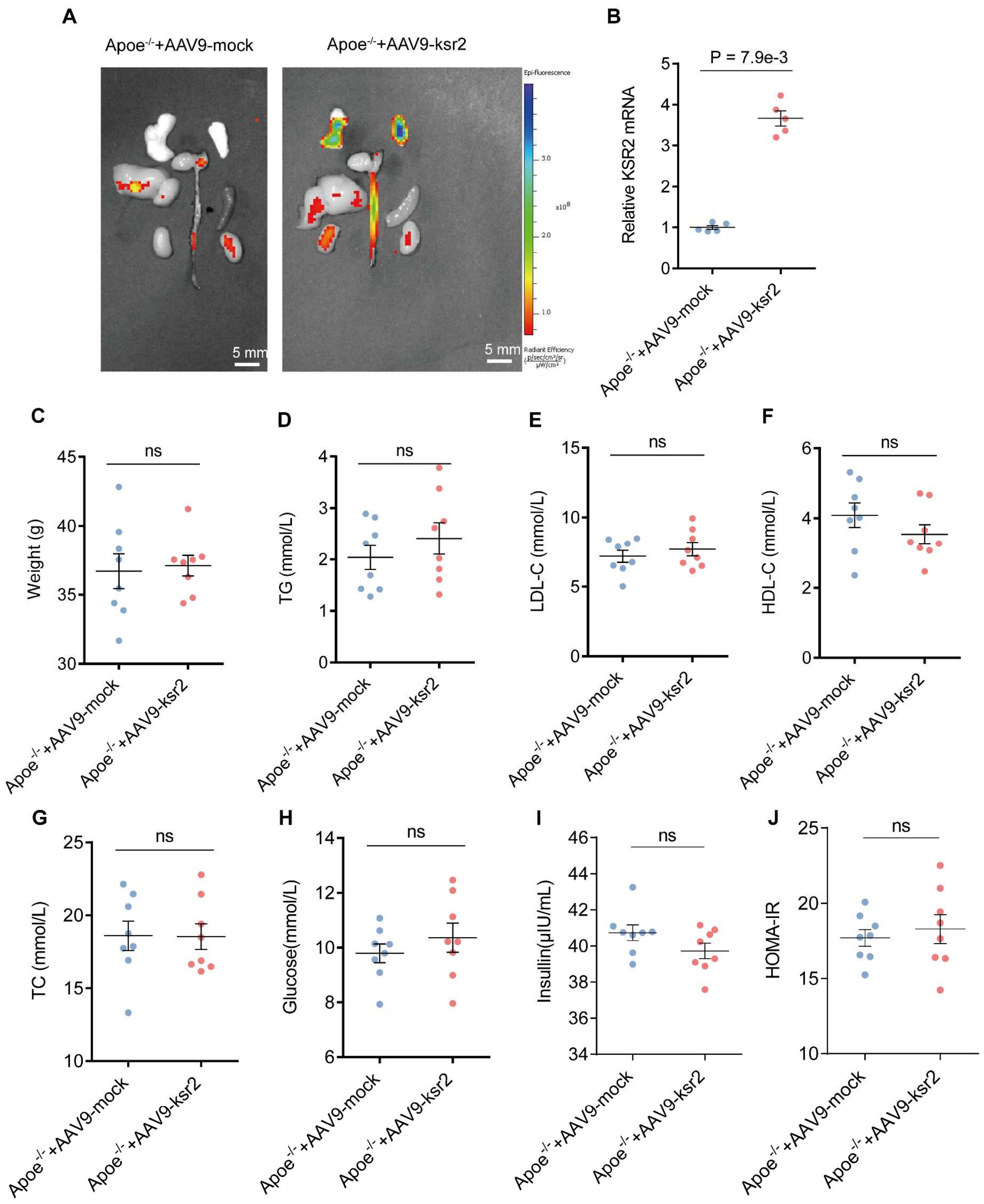
A
Apoe-/-+AAV9-mock
Apoe-/-+AAV9-ksr2
5 mm
5 mm
Epi-Fluorescence
Radiant Efficiency
B
P = 7.9e-3
Relative KSR2 mRNA
C
Weight (g)
D
TG (mmol/L)
E
LDL-C (mmol/L)
F
HDL-C (mmol/L)
G
TC (mmol/L)
H
Glucose(mmol/L)
I
Insullin(μIU/mL)
J
HOMA-IR
ns

Figure S6

Effect of palmitic acid stimulation on endothelial *KSR2* expression. Western blot and RT-qPCR analyses were performed to assess *KSR2* expression in HUVECs following time-course stimulation with palmitic acid (PA, 200 μmol/L). Relative expression levels were normalized to the 0 h control group. n = 5, Kruskal-Wallis test with Dunn's multiple comparisons test due to small sample sizes.

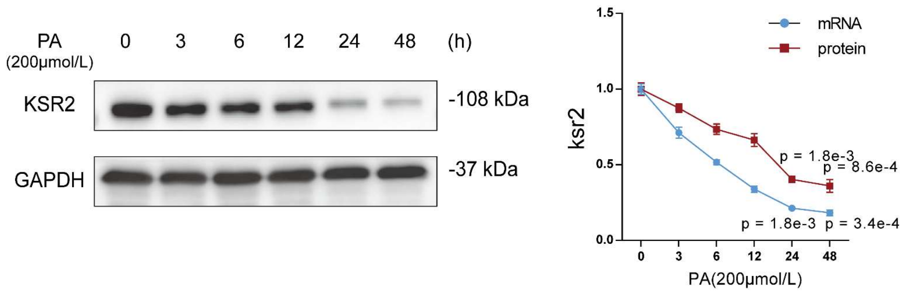

Figure S7

RNA-Seq analysis reveals the regulatory role of *KSR2* in HUVECs. A, Western blot analysis was used to detect *KSR2* expression in lentiviral stable-transfected HUVECs overexpressing *KSR2* (lenti-*KSR2*) and control (lenti-NC) cells. **B,** Volcano plots showing differentially expressed genes (DEGs) in lenti-NC and lenti-*KSR2* HUVECs. The plot highlights 535 upregulated genes (represented by red dots) and 444 downregulated genes (represented by green dots). n = 3. **C,** KEGG pathway analysis of DEGs identified in lenti-NC and lenti-*KSR2* HUVECs. **D,** GO enrichment analysis of DEGs identified in lenti-NC and lenti-*KSR2* HUVECs.

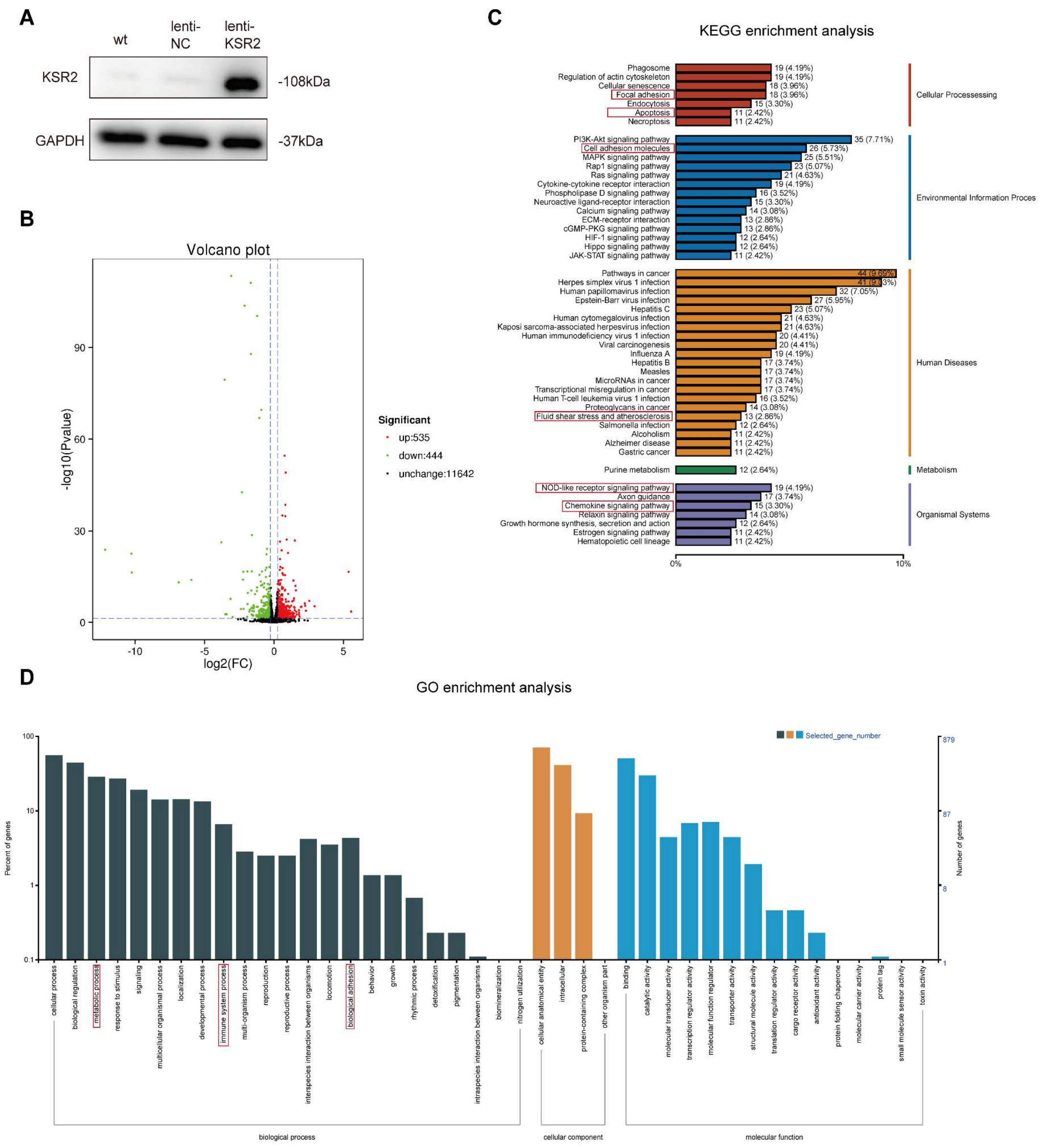

Figure S8

Validation of *KSR2* knockdown efficiency in HUVECs. RT-qPCR analysis confirmed that siRNA-mediated knockdown of *KSR2* (si-*KSR2*) significantly reduced *KSR2* mRNA levels in HUVECs. n = 5; Kruskal–Wallis test with Dunn's multiple comparisons test was used due to small sample sizes. ns, not significant.

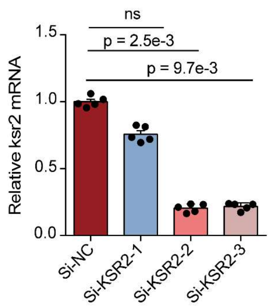

Figure S9

Endothelial *KSR2* mitigates atherosclerosis by inhibiting inflammation and apoptosis. A, Immunohistochemical staining of VCAM-1, ICAM-1, *TNF-α*, and IL-1β in the aortic roots of $Apoe^{-/-}$ + AAV9-mock and $Apoe^{-/-}$ + AAV9-*KSR2* mice. Scale bar = 400 μm. n = 8, 2-tailed unpaired Student's t-test. **B,** Representative en face TUNEL staining of endothelial cells in the aorta of $Apoe^{-/-}$ + AAV9-mock and $Apoe^{-/-}$ + AAV9-*KSR2* mice. TUNEL-positive cells are shown in red, *CD31* in green, and nuclei (DAPI) in blue. Scale bar = 20 μm. n = 5, 2-tailed unpaired Mann-Whitney U-test due to small sample sizes. **C,** Western blot analysis of cleaved caspase-3, Bcl-2, and *Bax* protein levels in aortic tissues from $Apoe^{-/-}$ + AAV9-mock and $Apoe^{-/-}$ + AAV9-*KSR2* mice. n = 6, Statistical analysis of *Bax*/Bcl-2 results was performed using a 2-tailed unpaired Mann–Whitney U test due to non-normally distributed data, cleaved caspase-3 results were analyzed using a 2-tailed unpaired Student's t-test. **D, E, F and G,** HUVECs were transfected with Lenti-NC or Lenti-*KSR2* for 48 h, followed by treatment with oxLDL (100 μg/mL) for 24 h. **(D)** RT-qPCR was used to measure the mRNA levels of VCAM-1, ICAM-1, *TNF-α*, and IL-1β. n = 6, 2-tailed Mann–Whitney U tests (multiple t-test framework, one per gene) with Bonferroni adjustment to control the family-wise error rate. **(E)** THP-1 adhesion assay assessing the adhesive capacity of HUVECs following *KSR2* overexpressed. Results are presented as the percentage of endothelial cells with adherent THP-1 cells. Scale bar = 400 μm. n = 6, 2-tailed unpaired Student's t-test. **(F)** TUNEL staining analysis of apoptosis in HUVECs following *KSR2* overexpressed. Results are presented as the percentage of TUNEL-positive cells. Scale bar = 200 μm. n = 6, 2-tailed unpaired Student's t-test. **(G)** WB analysis was used to assess the protein levels of cleaved caspase-3, Bcl-2, and *Bax*. n = 6, 2-tailed unpaired Student's t-test.

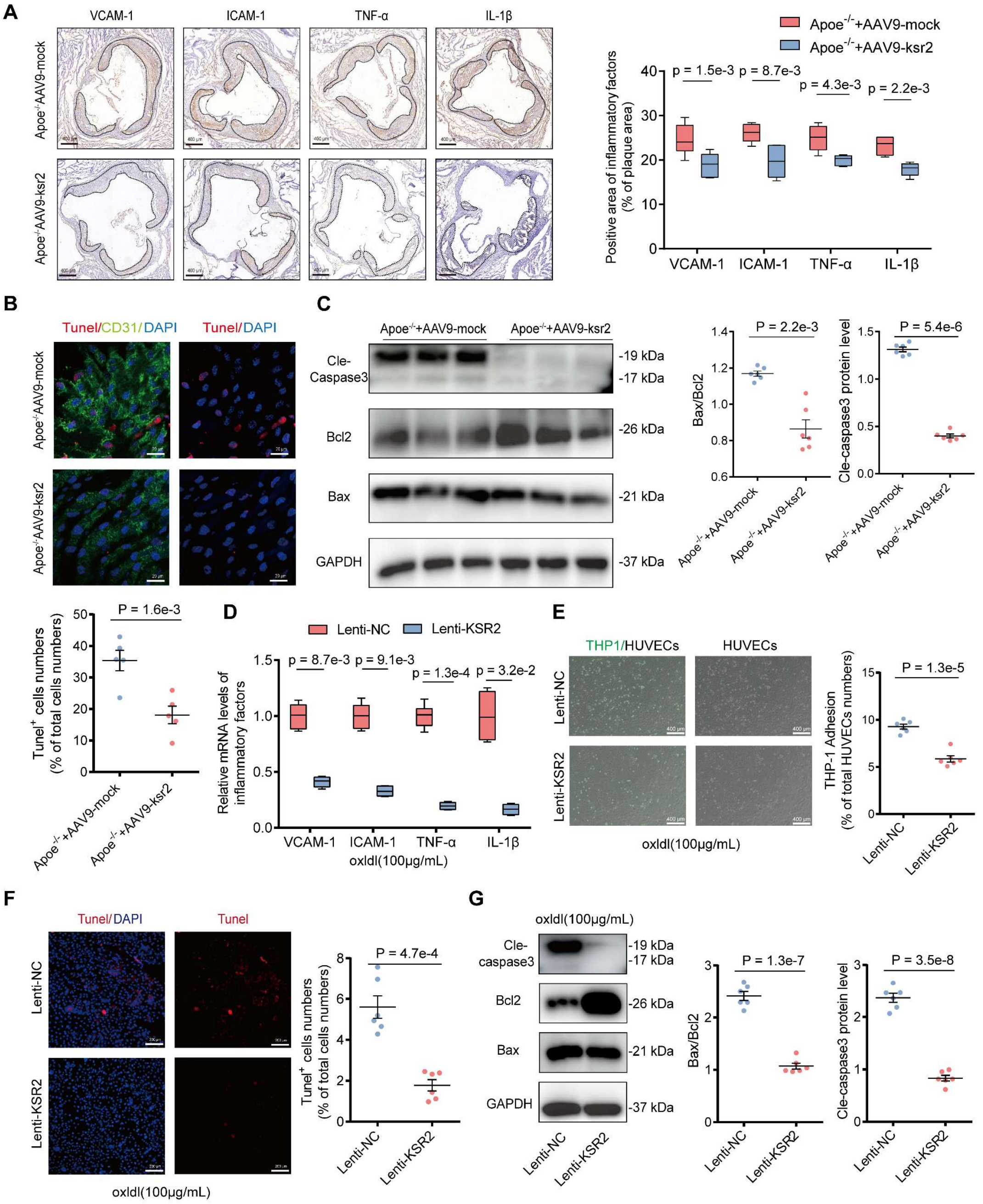

A
VCAM-1
ICAM-1
TNF-α
IL-1β
Apoe-/-AAV9-mock
Apoe-/-AAV9-ksr2
Apoe-/-+AAV9-mock
Apoe-/-+AAV9-ksr2
Positive area of inflammatory factors (% of plaque area)
p = 1.5e-3
p = 8.7e-3
p = 4.3e-3
p = 2.2e-3
B
Tunel/CD31/DAPI
Tunel/DAPI
Tunel+ cells numbers (% of total cells numbers)
P = 1.6e-3
C
Apoe-/-+AAV9-mock
Apoe-/-+AAV9-ksr2
Cle-Caspase3
Bcl2
Bax
GAPDH
-19 kDa
-17 kDa
-26 kDa
-21 kDa
-37 kDa
Bax/Bcl2
P = 2.2e-3
Cle-caspase3 protein level
P = 5.4e-6
D
Lenti-NC
Lenti-KSR2
Relative mRNA levels of inflammatory factors
p = 8.7e-3
p = 9.1e-3
p = 1.3e-4
p = 3.2e-2
oxldl(100μg/mL)
E
THP1/HUVECs
HUVECs
Lenti-NC
Lenti-KSR2
oxldl(100μg/mL)
THP-1 Adhesion (% of total HUVECs numbers)
P = 1.3e-5
F
Tunel/DAPI
Tunel
Lenti-NC
Lenti-KSR2
oxldl(100μg/mL)
Tunel+ cells numbers (% of total cells numbers)
P = 4.7e-4
G
oxldl(100μg/mL)
Cle-caspase3
Bcl2
Bax
GAPDH
-19 kDa
-17 kDa
-26 kDa
-21 kDa
-37 kDa
Lenti-NC
Lenti-KSR2
Bax/Bcl2
P = 1.3e-7
Cle-caspase3 protein level
P = 3.5e-8

Figure S10

***KSR2* overexpression in HUVECs maintains glycolytic balance. A-C,** lenti-*KSR2* and lenti-NC HUVECs were treated with oxLDL (100 μg/mL) or control for 24 h. **(A)** RT-qPCR analysis of mRNA levels of *HK-2* (left) and *PFKFB-3* (right) in the cells. n = 6, ordinary one-way ANOVA. **(B)** The extracellular acidification rate (ECAR) profile was used to assess the glycolytic function of HUVECs. Vertical lines indicate the addition of glucose (10 mM), oligomycin (1 μM), and 2-DG (50 mM). n = 10, Kruskal–Wallis test with Dunn's multiple comparisons test due to non-normally distributed data. **(C)** The cellular ATP/ADP ratio was determined to evaluate energy metabolic shifts in HUVECs. n = 6, ordinary one-way ANOVA. **D,** RT-qPCR analysis of PFKFB3 and HK2 mRNA levels in the aortic tissues of Apoe$^{-/-}$ + AAV9-mock and Apoe$^{-/-}$ + AAV9-*KSR2* mice. n = 6, 2-tailed Mann–Whitney U tests (multiple t-test framework, one per gene) with Bonferroni adjustment to control the family-wise error rate. **E,** Representative en face immunofluorescence staining of PFKFB3 and *HK-2* in endothelial cells of the aorta from Apoe$^{-/-}$ + AAV9-mock and Apoe$^{-/-}$ + AAV9-*KSR2* mice. PFKFB3 or *HK-2* is shown in red, *CD31* in green, and DAPI in blue. Scale bar = 40 μm. n = 6, 2-tailed unpaired Student's t-test. ns, not significant.

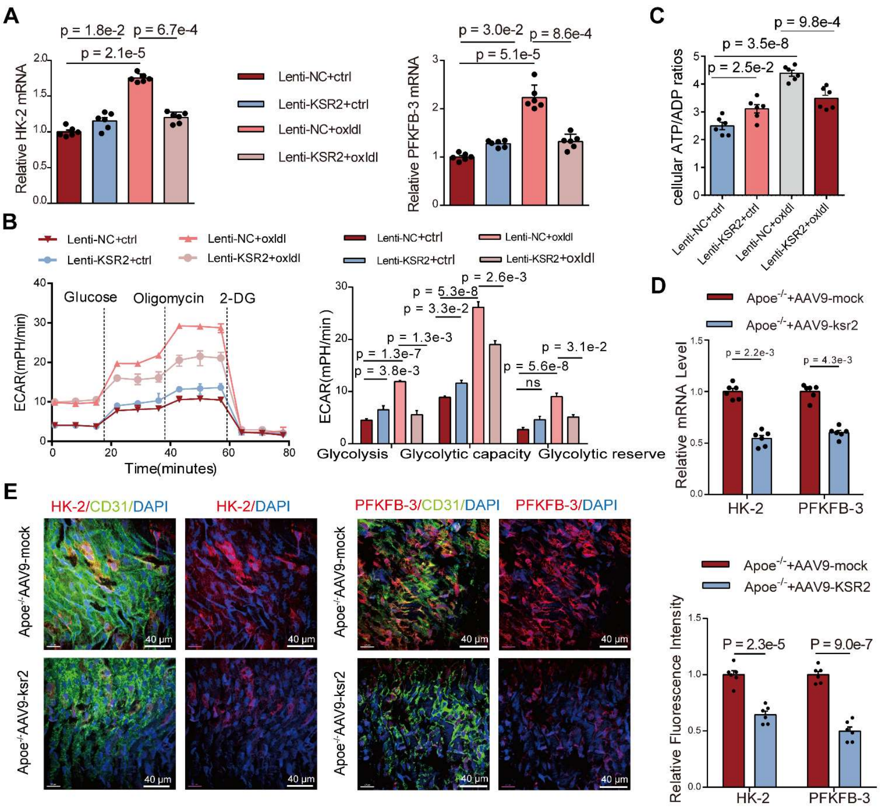

Figure S11

In vivo validation of the effect of endothelial *KSR2* overexpression on AMPK signaling pathway. A, Western blot was used to assess the levels of p-*AMPKα*^{T172}, *AMPKα*1, *AMPKα*2, p-*ACC1*^{S79}, and *ACC1* proteins in the aortic tissue of Apoe$^{-/-}$ + AAV9-mock and Apoe$^{-/-}$ + AAV9-*KSR2* mice. n = 6, statistical analysis of p-*AMPKα*^{T172}, *AMPKα*1 results were performed using 2-tailed unpaired Mann–Whitney U test due to non-normally distributed data, levels of *AMPKα*2, p-*ACC1*^{S79}, and *ACC1* were analyzed with 2-tailed unpaired Student's t-test. **B,** RT-qPCR was used to investigate the mRNA levels of *AMPKα*1 and *AMPKα*2 in the aortic tissue of Apoe$^{-/-}$ + AAV9-mock and Apoe$^{-/-}$ + AAV9-*KSR2* mice. n = 6, 2-tailed Mann–Whitney U tests (multiple t-test framework, one per gene) with Bonferroni adjustment to control the family-wise error rate. ns, not significant.

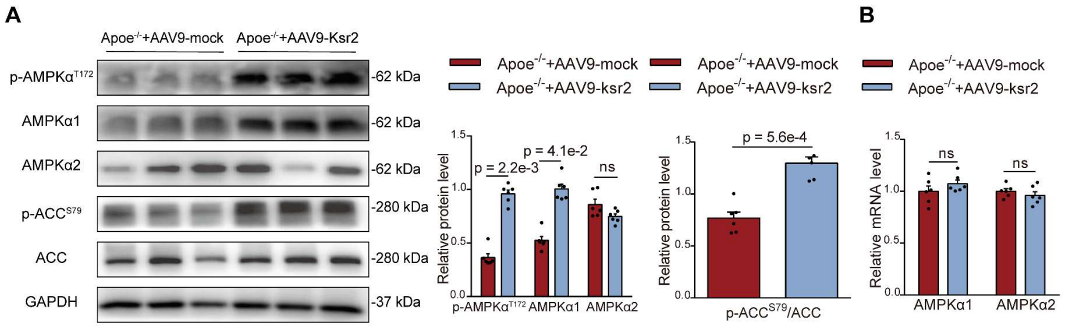

Figure S12

Mechanistic validation of *KSR2*-mediated regulation of *AMPKα*1 protein stability. HUVECs were transfected with control siRNA (si-NC) or *KSR2* siRNA (si-*KSR2*) for 48 h, followed by pre-treatment with **(A)** chloroquine (CQ; 10 mM), **(B)** 3-methyladenine (3-MA; 10 mM) and subsequent oxLDL (100 μg/mL) stimulation for 24 h. WB analysis was performed to measure *AMPKα*1 protein levels. n = 6, ordinary one-way ANOVA. ns, not significant.

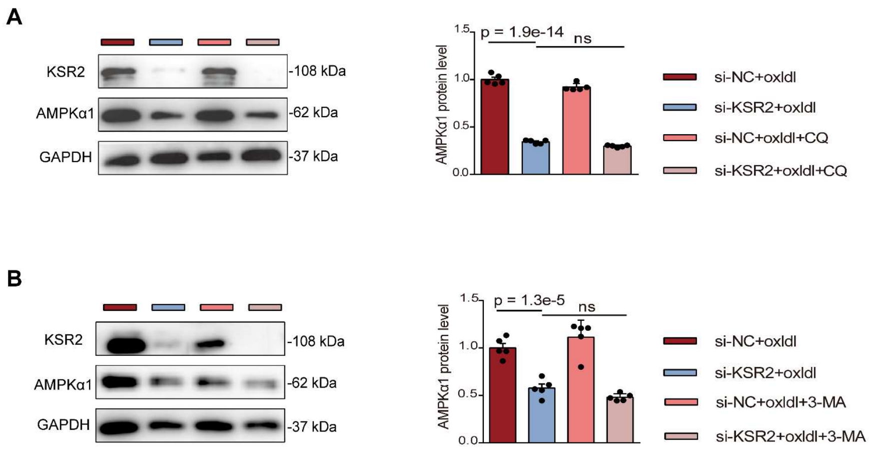

Figure S13

Investigation of the specific ubiquitin chain type mediating *KSR2*-regulated *AMPKα*1 ubiquitination in HEK293T cells. HEK293T cells were transfected with *MYC-KSR2*, *FLAG-AMPKα*1, *HA*-UB(M0) (M0-specific mutant), *HA*-UB(K6) (lysine 6-specific mutant), *HA*-UB(K11) (lysine 11-specific mutant), *HA*-UB(K27) (lysine 27-specific mutant), *HA*-UB(K29) (lysine 29-specific mutant), *HA*-UB(K33) (lysine 33-specific mutant) and appropriate control plasmids for 48 h, then treated with MG132 (10 μM) for 24 h. Co-IP and immunoblotting were performed to test the ubiquitination of exogenous *AMPKα*1 via immunoprecipitation of *FLAG*-tagged *AMPKα*1.

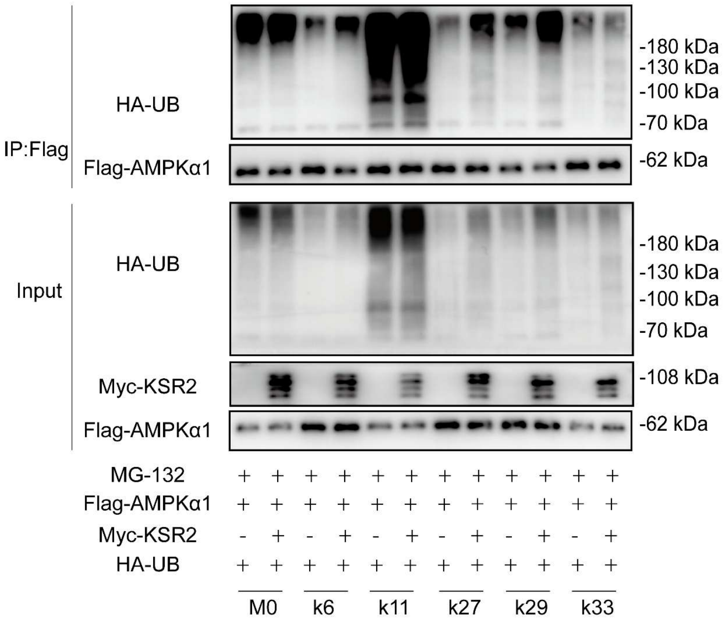

Figure S14

Identification of E3 ubiquitin ligases mediating *KSR2*-stabilized *AMPKα*1 protein. HUVECs were transfected with siRNAs targeting **(A)** ***MKRN1***, **(B)** *RMND5A,* **(C)** TRIM28, **(D)** PEDF, **(E)** Fbxo48, or **(F)** RNF44, or pretreated with **(G)** TD165 (0.5 μM) or **(H)** lenalidomide (1 μM), in combination with *KSR2* siRNA for 48 hours, followed by oxLDL stimulation (100 μg/mL) for 24 hours. *AMPKα*1 protein levels were assessed by Western blotting. **A–F**: n = 5, Kruskal–Wallis test with Dunn's multiple comparisons test due to small sample sizes. **G** and **H**: n = 6, ordinary one-way ANOVA. ns, not significant.

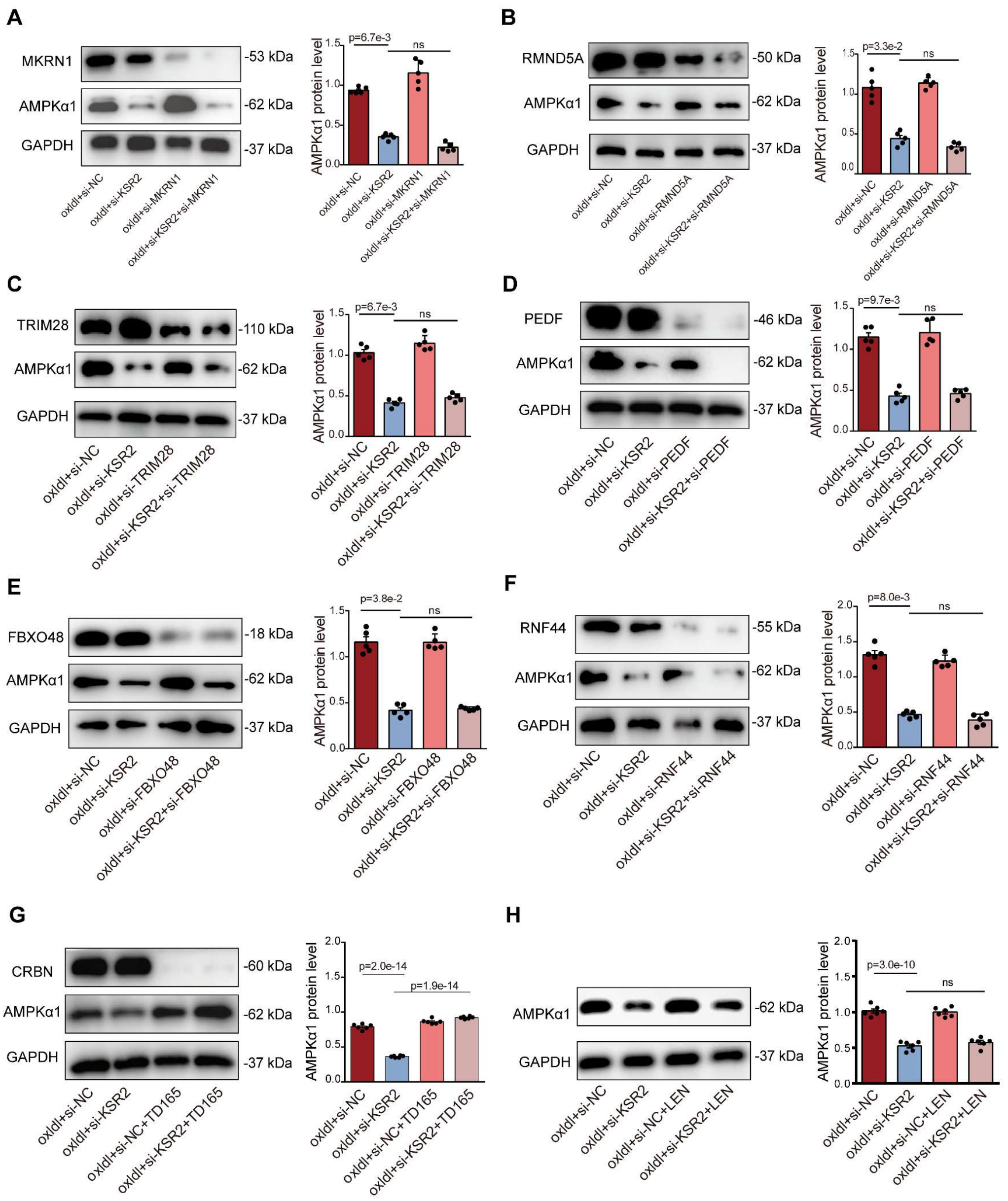

Figure S15

Subcellular localization of *CUL4A*, *DDB1*, and *CRBN* in endothelial cells following oxLDL stimulation. **A** and **B**, HUVECs were treated with oxLDL (100 μg/mL) or vehicle for 24 h. **(A)** Total, nuclear, and cytoplasmic proteins were extracted, and WB was used to analyze the subcellular distribution of *CUL4A*, *DDB1*, and *CRBN*. **(B)** Immunofluorescence co-localization analysis was performed to examine the cellular localization of *CUL4A*, *DDB1*, and *CRBN*. Scale bars = 40 μm.

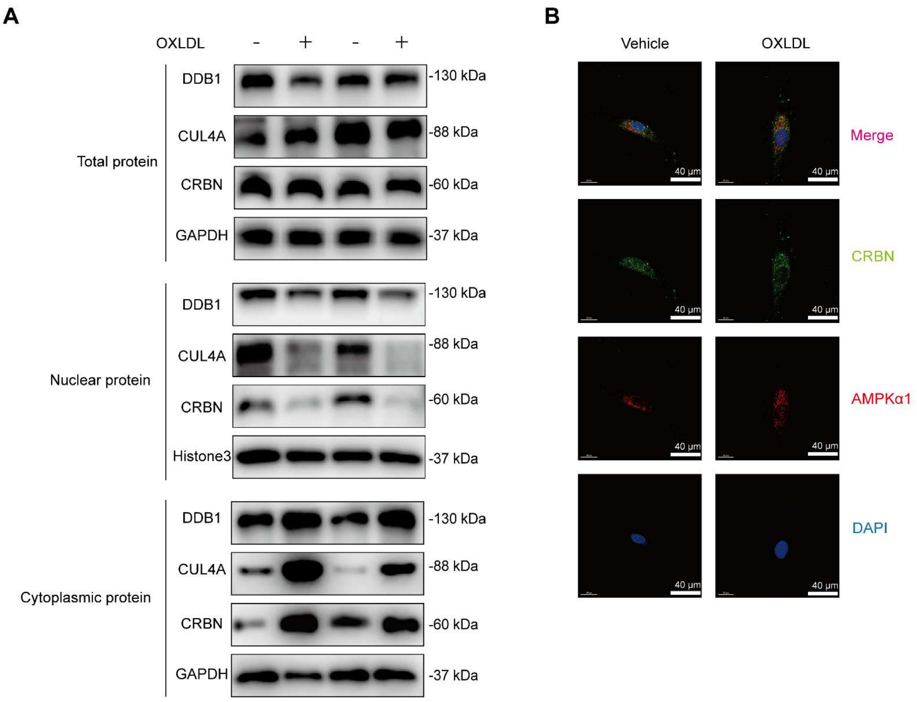

Figure S16

Specific knockdown of endothelial *CRBN* in mice via AAV9-sh*CRBN*. Frozen sections of mouse aortic roots were subjected to double immunofluorescence staining for *CRBN* and the endothelial marker *CD31*, smooth muscle cell marker *α-SMA*, or macrophage marker *MOMA-2*. Mean fluorescence intensity (MFI) was used to quantify the protein level of *CRBN* in plaque cells. n = 6, 2-tailed unpaired Student's t-test. ns, not significant.

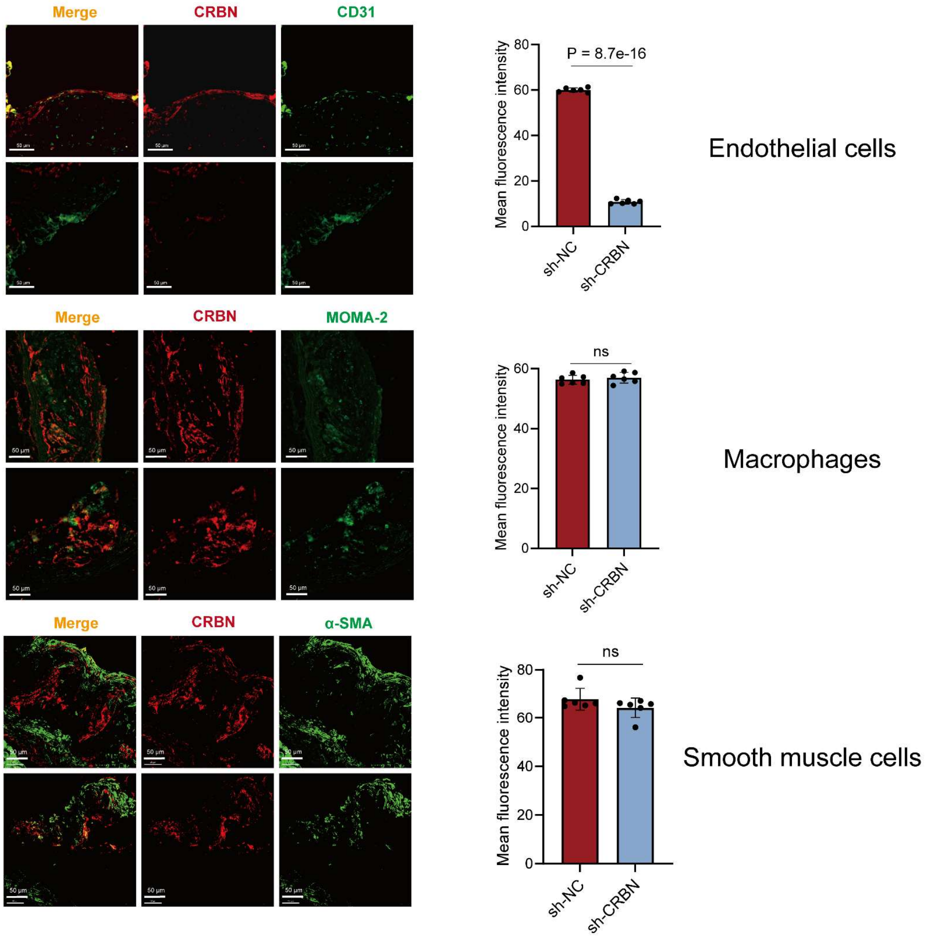

Figure S17

Endothelial *CRBN* knockout suppresses endothelial inflammation and apoptosis, and mitigates atherosclerosis in ApoE$^{-/-}$ mice. Endothelial-specific *CRBN* knockout was achieved via tail vein injection of AAV9-ICAM2-sh*CRBN*, while control mice received an equivalent dose of AAV9-ICAM2-shNC. All groups were subsequently pair-fed a high-fat diet for 8 weeks. **A,** Representative en face Oil Red O staining of aortas from ApoE$^{-/-}$ + sh-NC and ApoE$^{-/-}$ + sh-*CRBN* mice. Quantification of aortic lesion areas is shown. Scale bar = 5 mm. n = 8. 2-tailed unpaired Student's t-test. **B-F,** Representative Oil Red O staining **(B)**, H&E staining **(C)**, immunohistochemical staining for *MOMA-2* **(D)** and *α-SMA* **(E)**, and Masson's trichrome staining **(F)** of aortic root sections from ApoE$^{-/-}$ + sh-NC and ApoE$^{-/-}$ + sh-*CRBN* mice. Quantification of aortic lesion areas is shown. Scale bar = 400 μm. n = 8. Statistical analysis was performed using 2-tailed unpaired Student's t-test, except for *MOMA-2* staining results, which were analyzed using the 2-tailed unpaired Mann–Whitney U test due to non-normal distribution. **G,** Immunohistochemical staining of VCAM-1, *TNF-α* in the aortic roots of ApoE$^{-/-}$ + sh-NC, ApoE$^{-/-}$ + sh-*CRBN* mice. Scale bar = 200 μm. n = 8, 2-tailed unpaired Student's t-test. **H,** RT-qPCR analysis of VCAM-1 and *TNF-α* mRNA expression in aortas from ApoE$^{-/-}$ + sh-NC， ApoE$^{-/-}$ + sh-*CRBN* mice**.** n = 6, 2-tailed unpaired Student's t-test. **I,** Representative en face TUNEL staining of aortic endothelium from ApoE$^{-/-}$ + sh-NC， ApoE$^{-/-}$ + sh-*CRBN* mice. TUNEL-positive cells are shown in red, *CD31* in green, and nuclei (DAPI) in blue. Scale bar = 20 μm. n = 6, 2-tailed unpaired Student's t-test. **J,** Western blot analysis of *AMPKα*1, cleaved caspase-3, Bcl-2, and *Bax* protein levels in aortic tissues from ApoE$^{-/-}$ + sh-NC, ApoE$^{-/-}$ + sh-*CRBN* mice. n = 6, statistical analysis was performed using 2-tailed unpaired Student's t-test, except for *Bax*/Bcl-2 ratio, which was analyzed using the 2-tailed unpaired Mann–Whitney U test due to non-normal distribution.

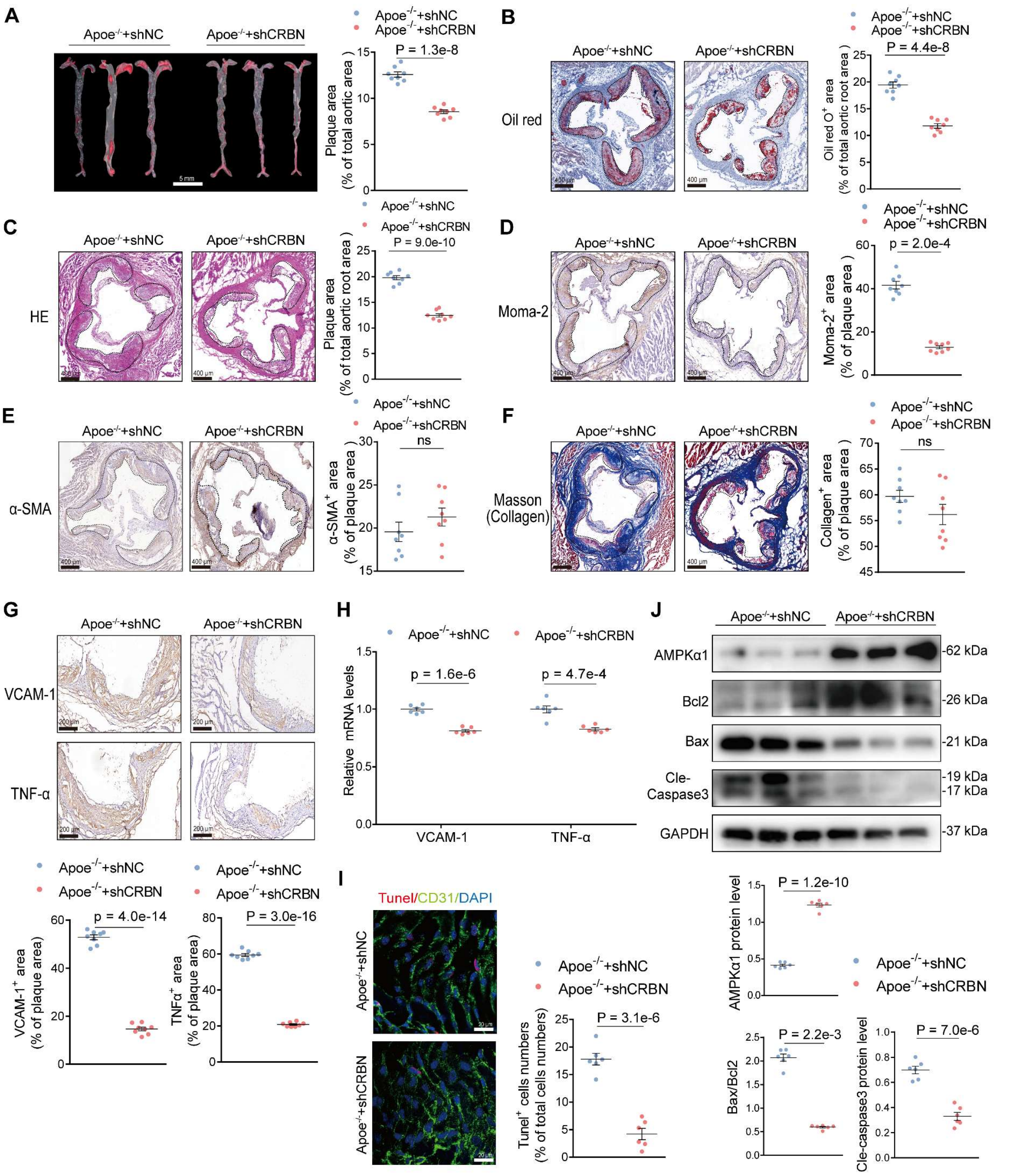

Figure S18

Endothelial *KSR2* Attenuates Atherosclerosis Progression via *CRBN* in Apoe$^{-/-}$ Mice. A, Representative en face Oil Red O-stained aortas from ApoE$^{-/-}$ + AAV9-mock, ApoE$^{-/-}$+ AAV9-*KSR2*, ApoE$^{-/-}$ + AAV9-*KSR2*+ AAV9-*CRBN* mice. Quantification of aortic lesion areas is shown. Scale bar = 5 mm. n = 8, ordinary one-way ANOVA. **B,** Representative Oil Red O and H&E staining of aortic root cryosections from ApoE$^{-/-}$ + AAV9-mock, ApoE$^{-/-}$+ AAV9-*KSR2*, ApoE$^{-/-}$+ AAV9-*KSR2*+ AAV9-*CRBN* mice. Quantification of aortic lesion areas is shown. Scale bar = 400 μm. n = 8, ordinary one-way ANOVA.

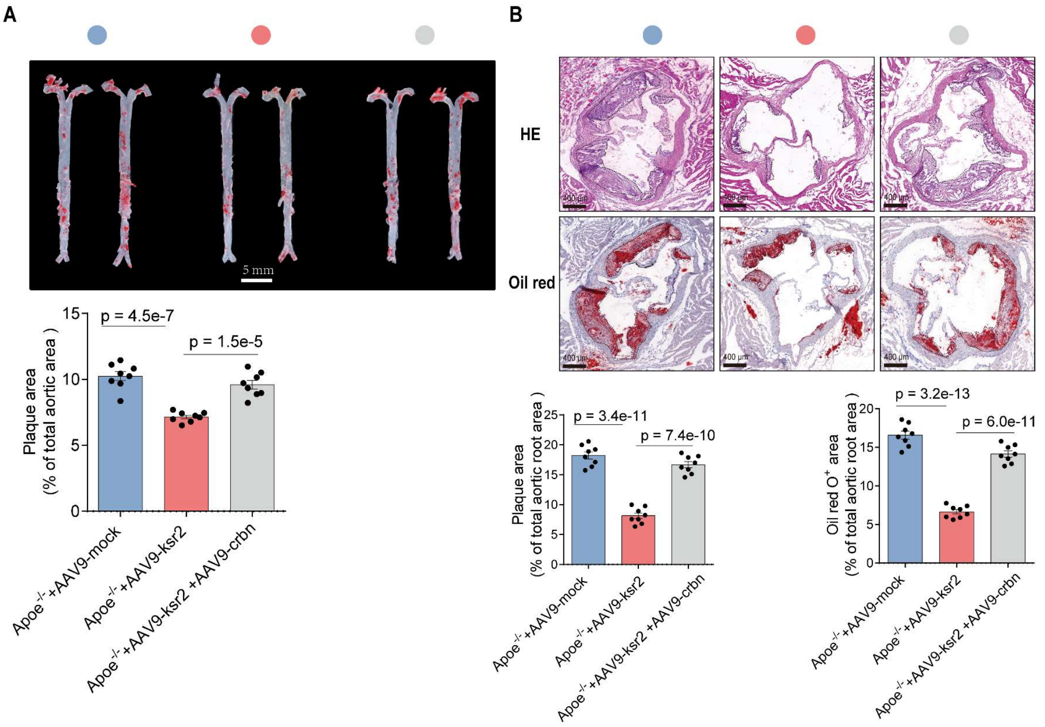

Figure S19

Endothelial *KSR2* maintains glycolytic homeostasis via *AMPKα*1. Lentivirus-stably transfected lenti-*KSR2* HUVECs and lenti-NC HUVECs were transfected with si-NC or si-*AMPKα*1 (#1 + 2) for 48 h, followed by treatment with or without oxLDL (100 μg/mL) for 24 h. The cellular ATP/ADP ratio was determined to evaluate energy metabolic shifts in HUVECs. n = 6, ordinary one-way ANOVA.

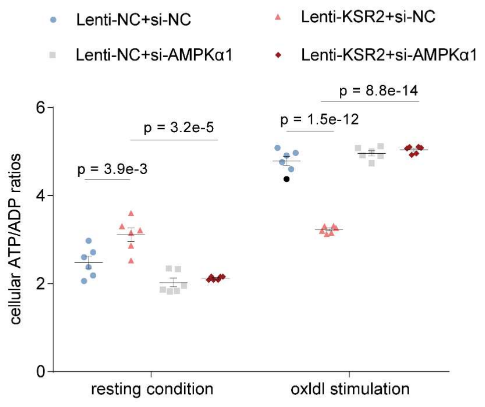

Supplement: Supplementary file 1 — Supplementary methods, figures and tables. [file thnov16p2598s1.pdf]
